# Supplementary material for: Tagged IDS causes efficient and engraftment-independent prevention of brain pathology during lentiviral gene therapy for Mucopolysaccharidosis type II
Source: Mol Ther Methods Clin Dev. 2023 Nov 2;31:101149. doi: 10.1016/j.omtm.2023.101149 (PMC10684800; doi:10.1016/j.omtm.2023.101149)
Supplement: Document S1. Figures S1–S10 and Tables S1 and S2 [file mmc1.pdf]

OMTM, Volume *31*

Supplemental information

Tagged IDS causes efficient and engraftment-independent prevention of brain pathology during lentiviral gene therapy for Mucopolysaccharidosis type II

Fabio Catalano, Eva C. Vlaar, Drosos Katsavelis, Zina Dammou, Tessa F. Huizer, Jeroen C. van den Bosch, Marianne Hoogeveen-Westerveld, Hannerieke J.M.P. van den Hout, Esmeralda Oussoren, George J.G. Ruijter, Gerben Schaaf, Karin Pike-Overzet, Frank J.T. Staal, Ans T. van der Ploeg, and W.W.M. Pim Pijnappel

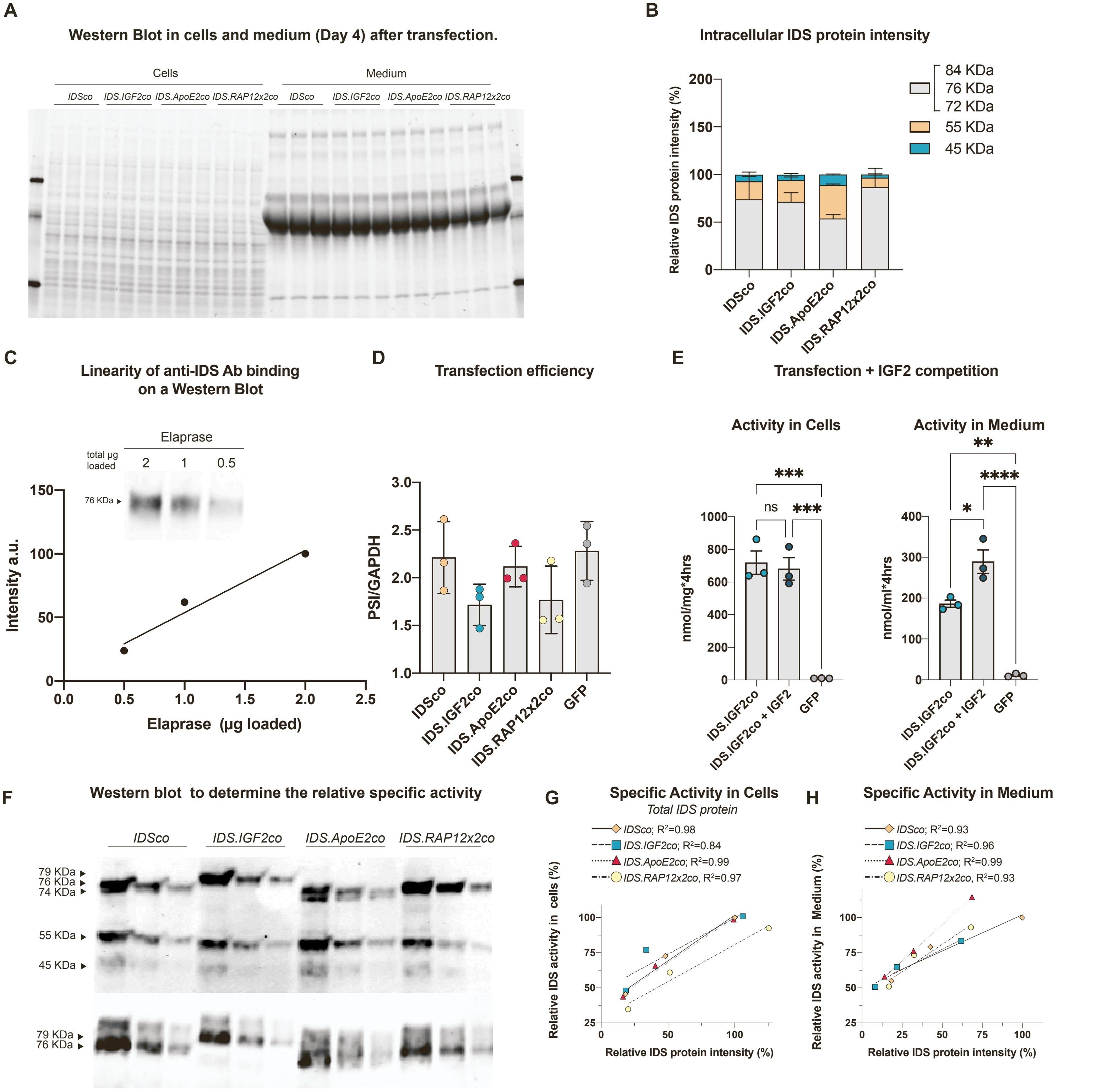

Figure S1. Control experiments for *in vitro* characterization of tagged IDS proteins.
(A) 20 µg total protein load for the western blot in Figure 1B. Equal loading was determined by quantification of the stain free signal of the same gel used for immunoblot analysis. Quantification of Figure 1B is shown in (B), where each processing form is expressed as relative of the total amount of protein. (C) Linearity of anti-IDS antibody binding on a western blot. (D) Transfection efficiency based on mRNA expression of *PSI* present in the lentiviral vector. (E) Intracellular and secreted IDS activity levels after transient transfection with *IDS.IGF2co* and addition or not of 1.5 µM IGF2 peptide in medium. (F) IDS protein levels measured in 3 2-fold dilutions of cell lysate and medium supernatant of transfected HEK 293T cells. Samples from Figure 1B were pooled and diluted to equal IDS activity levels before loading on gel. (G-H) IDS protein levels on western blot (Figure S1F) and IDS enzyme activity were measured in 3 2-fold dilutions of cell lysate and medium supernatant of transfected HEK 293T cells to determine the specific activity in cells (G) and medium (H). X-axes in G and H show the total IDS protein quantified from C. Slope and specific activity values are shown in Table S1. Data represent means ± SEM and were analyzed by one-way ANOVA followed by Bonferroni's multiple testing correction. *n = 3* biological replicates/condition. *p ≤ 0.05, **p ≤ 0.01, ***p ≤ 0.001, ****p ≤ 0.0001."

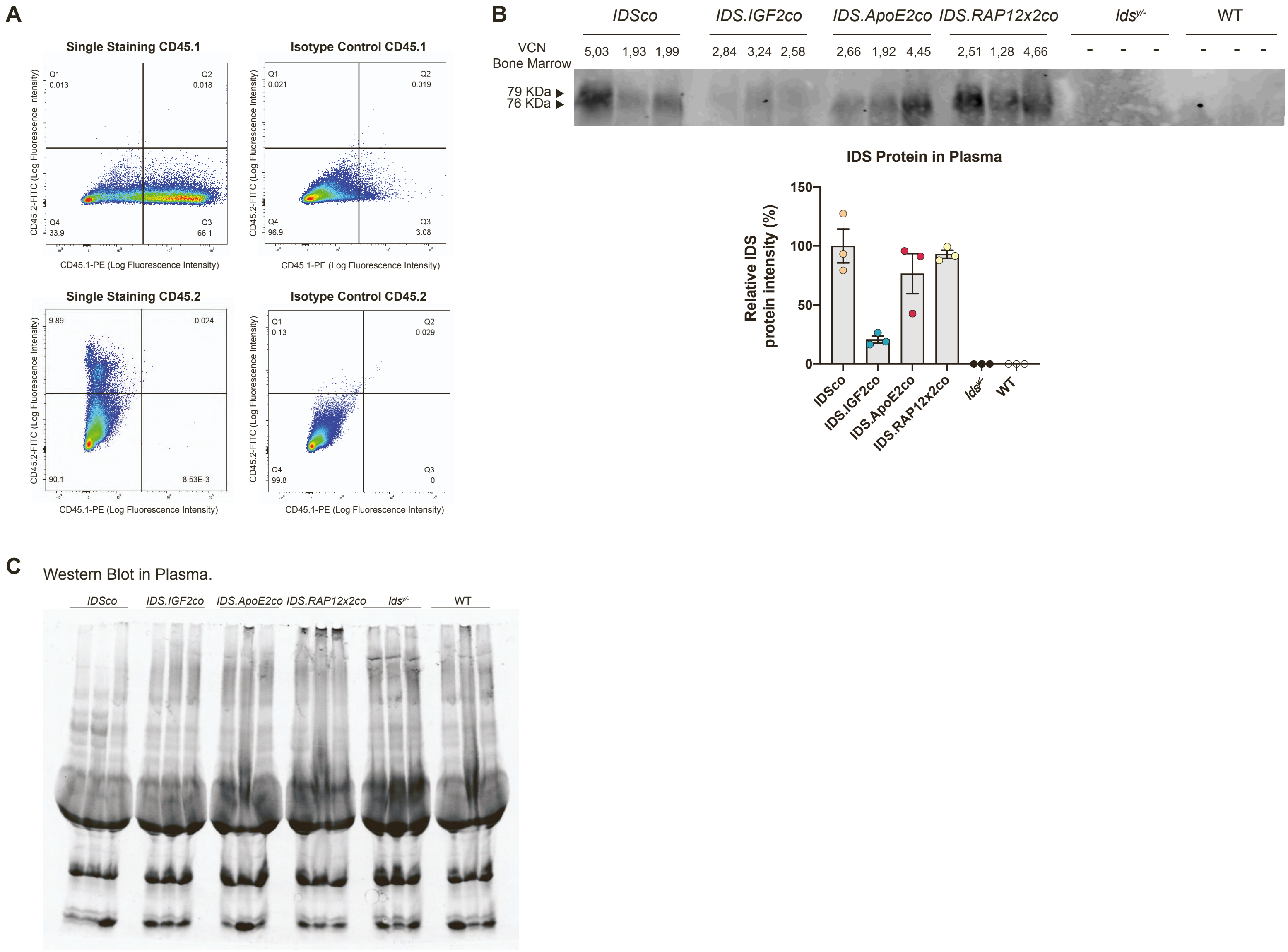

Figure S2. Chimerism control experiment and immunoblot of IDS protein in plasma.
(A) Single staining and isotype control staining of CD45.1 for measuring chimerism in bone marrow. (B) Immunoblot analysis of IDS protein in plasma after gene therapy. VCN in bone marrow for each sample is indicated. Quantification of IDS protein immunoblot in plasma is shown in the graph below. Equal loading was determined by quantification of the stain-free signal in (C), after loading of 276 µg of total protein. Data represent means ± SEM.

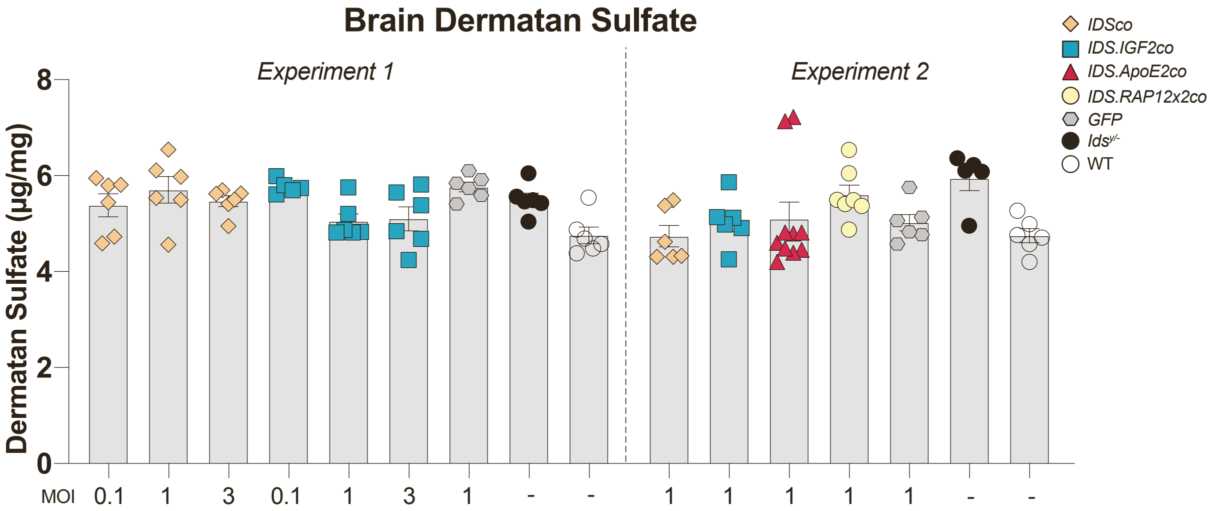

Figure S3. Quantification of brain dermatan Sulfate after gene therapy.
Mass spectrometry quantification of dermatan sulfate in brain homogenates of *Ids*$^{y/-}$ mice after gene therapy. Data are presented as means ± SEM and were analyzed by one-way ANOVA with Bonferroni's correction.

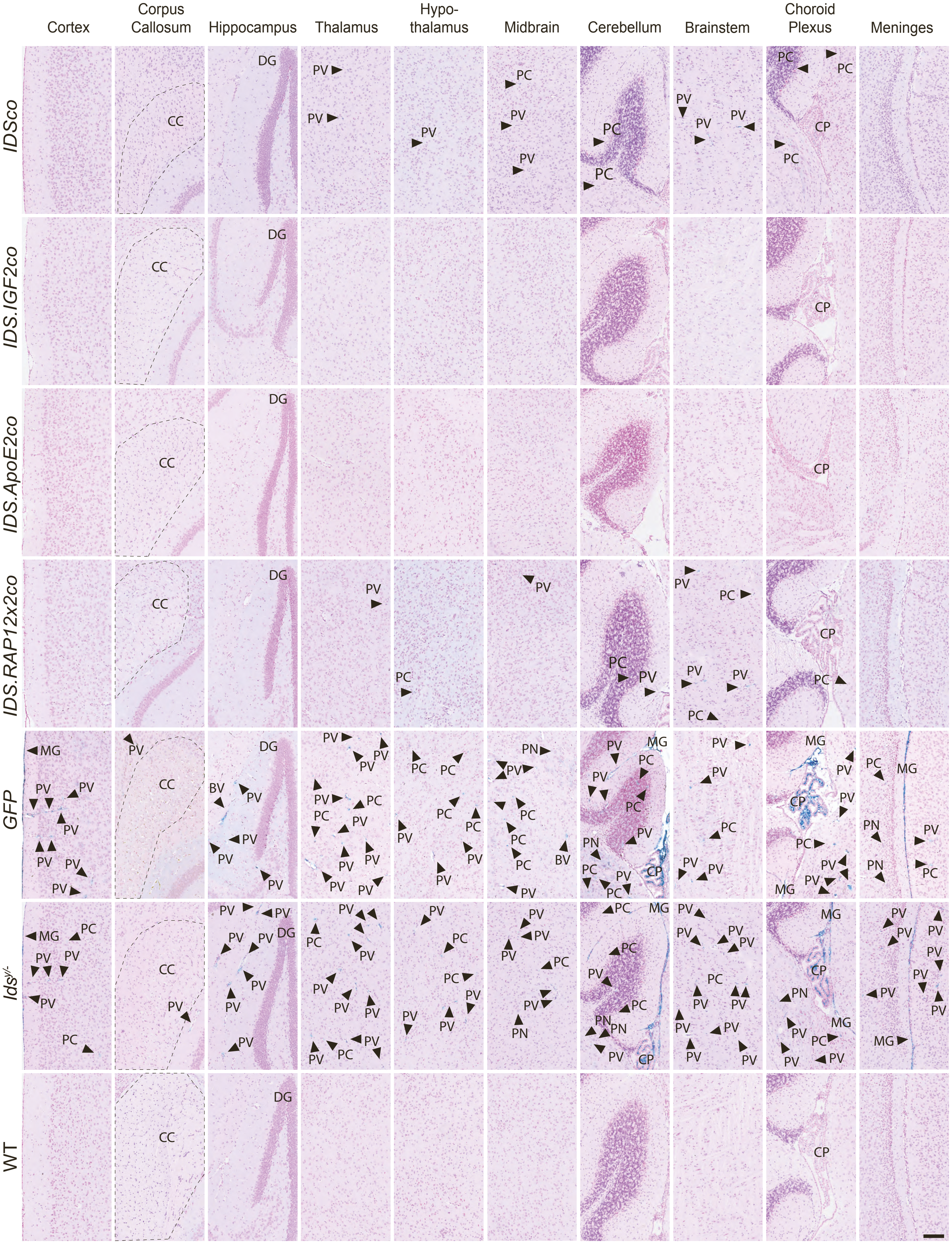

Figure S4. Alcian blue staining of *Ids*$^{y/-}$ brain after gene therapy

Alcian blue staining of sagittal sections of cortex, corpus callosum, hippocampus, thalamus, hypothalamus, midbrain, cerebellum, brainstem, choroid plexus and meninges of gene therapy treated *Ids*$^{y/-}$ mice. CC: corpus callosum; DG: dentate gyrus; BV: blood vessel; PC: parenchymal cell; CP: choroid plexus; Mg: meninges; PN: perineuronal net. *n = 3*. Scale bar = 100 µm.

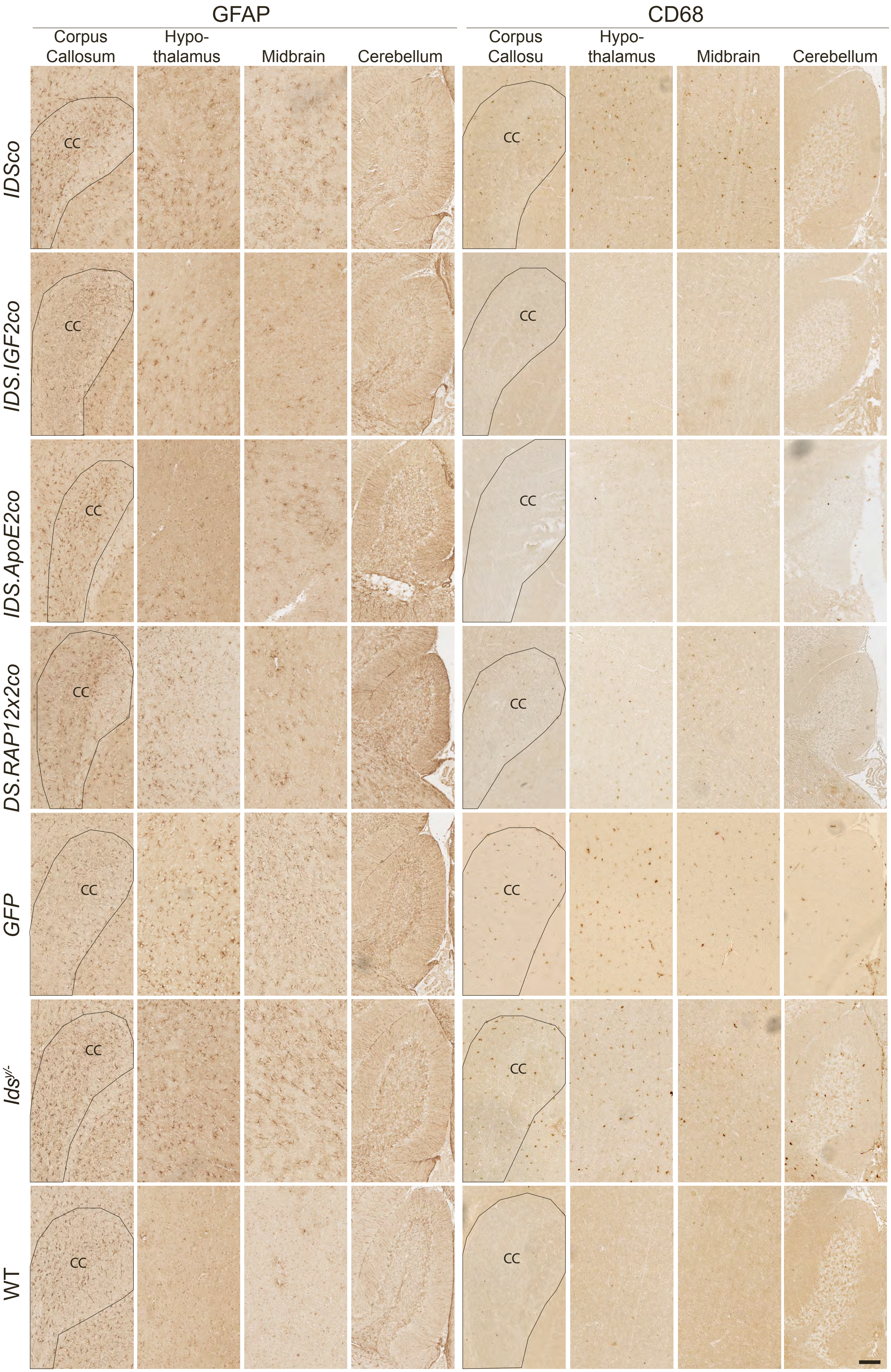

Figure S5. Gene therapy relieves neuroinflammation in corpus callosum, hypothalamus, midbrain and cerebellum. Sagittal sections of corpus callosum, hypothalamus, midbrain and cerebellum of gene therapy treated mice and controls stained for GFAP and CD68. CC: corpus callosum. *n = 3.* Scale bar = 100 µm.

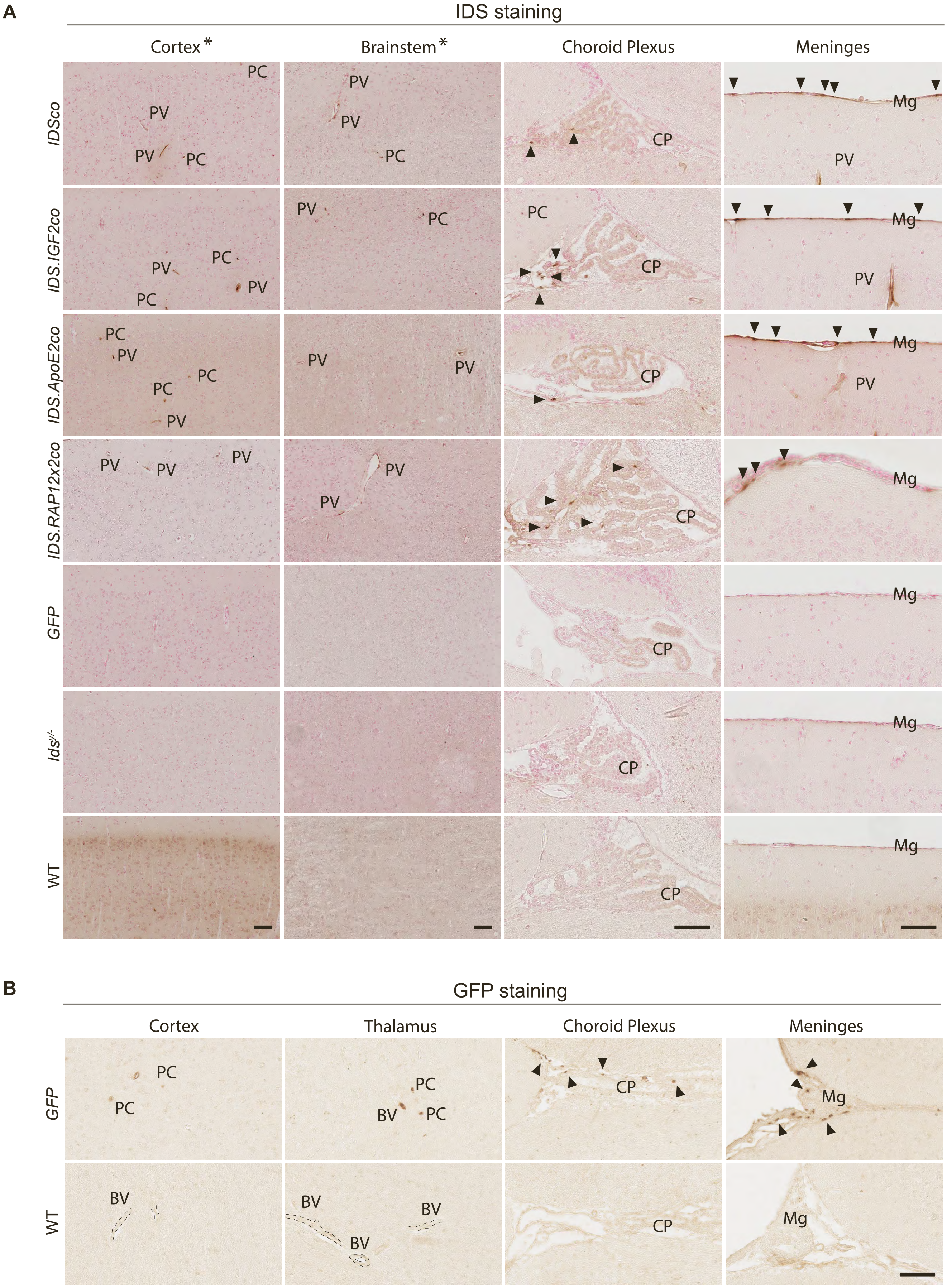

Figure S6. Engraftment of donor-derived cells in brain after gene therapy.
(A) Sagittal sections of cortex, brainstem, choroid plexus and meninges of gene therapy-treated and untreated *Ids*$^{y/-}$ mice and WT controls stained for IDS. Scale bar = 50 µm. (B) Sagittal sections of cortex, thalamus, choroid plexus and meninges stained for GFP. *n = 3*. Scale bar = 50 µm. BV: blood vessel; PC: parenchymal cell; PV: perivascular cells CP: choroid plexus; Mg: meninges. *Contrast was enhanced within the linear range in cortex and brainstem to highlight positive staining.

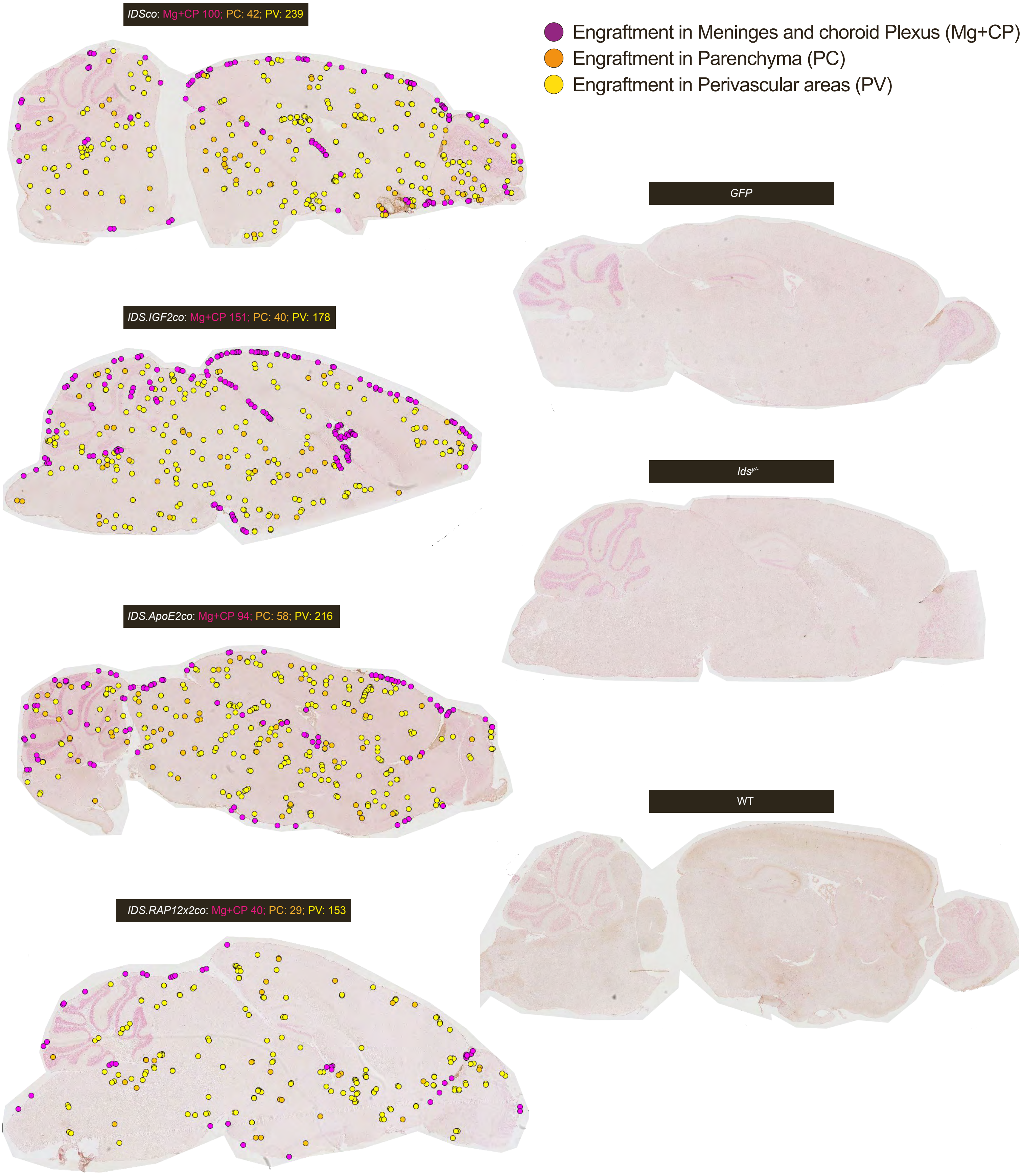

Figure S7. Distribution of IDS-positive cells in brain after gene therapy.
Examples of sagittal sections of gene therapy-treated and untreated *Ids*$^{y/-}$ mice and WT controls stained for IDS. IDS-positive cells are indicated by coloured dots based on the area of engraftment. Number of IDS-positive cells per brain area is indicated above each section.

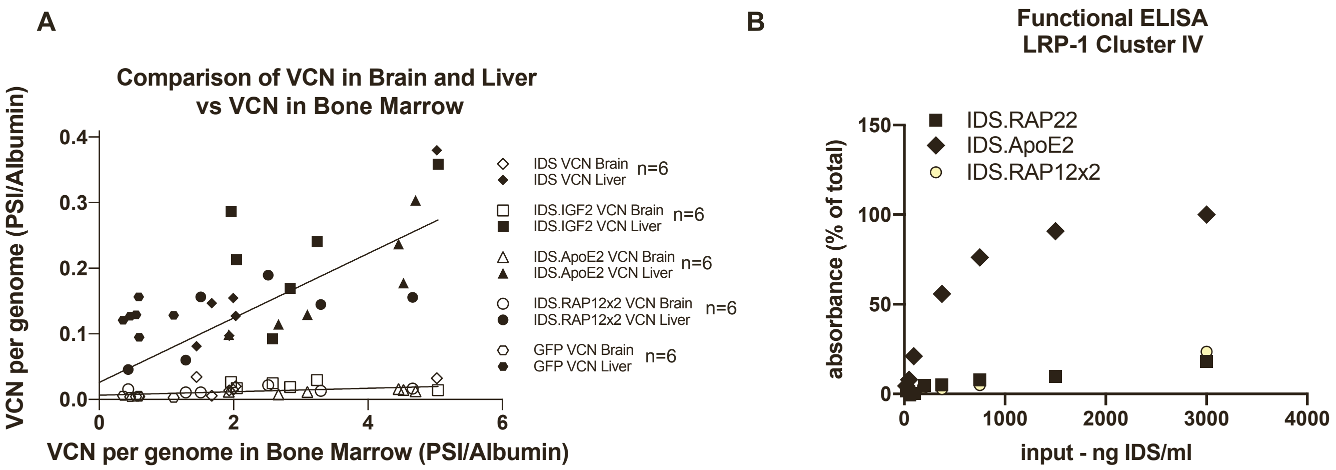

Figure S8. Comparison of VCN in liver and brain after gene therapy and LRP-1 ELISA for IDS.RAP22.
(A) Linear regression analysis between VCN in brain, VCN in liver and VCN in bone marrow. (B) Functional ELISA analysis of IDS.RAP22, IDS.ApoE2 and IDS.RAP12x2 proteins using the LRP-1 receptor (cluster IV).

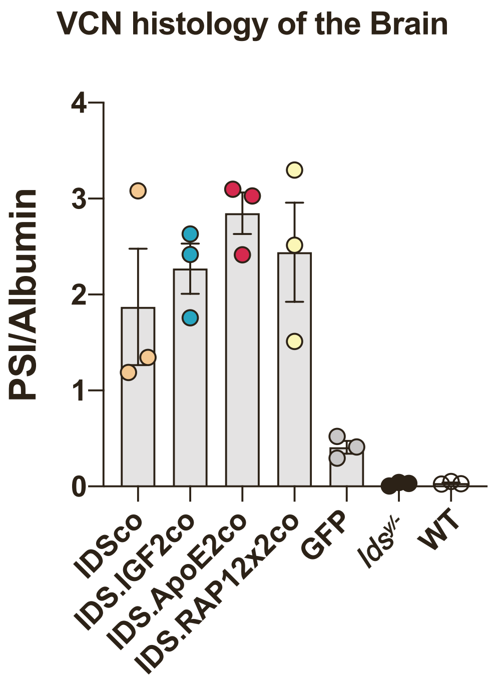

Figure S9. VCN in bone marrow of mice used for histology of the brain.
VCN in bone marrow of gene therapy-treated *Ids*$^{y/-}$ mice and control mice used for histology of the brain (Alcian Blue, LAMP1, GFAP, CD68 stainings). VCN per genome was measured by qPCR on *PSI* and *Albumin* loci. Data are presented as means ± SEM and were analyzed by one-way ANOVA with Bonferroni's correction.

A

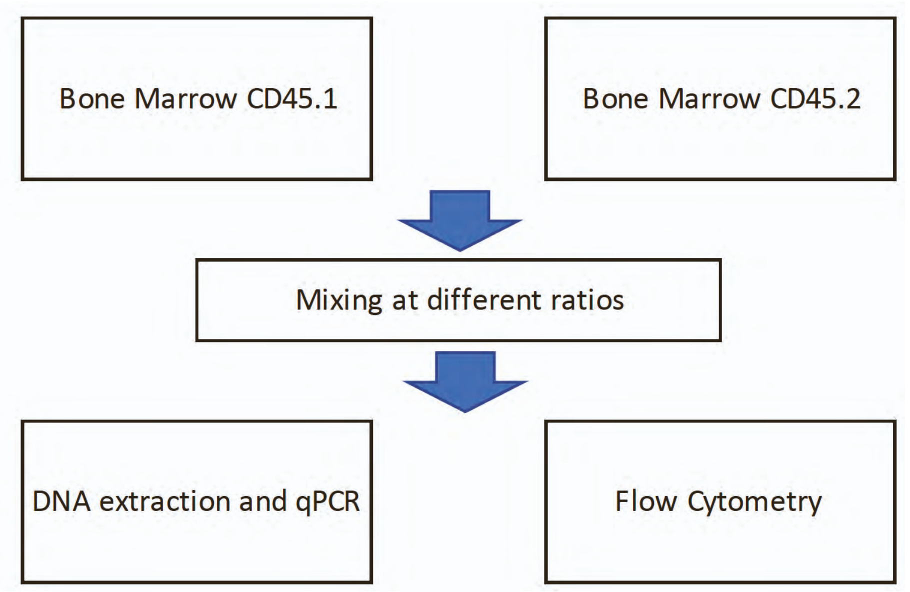

B

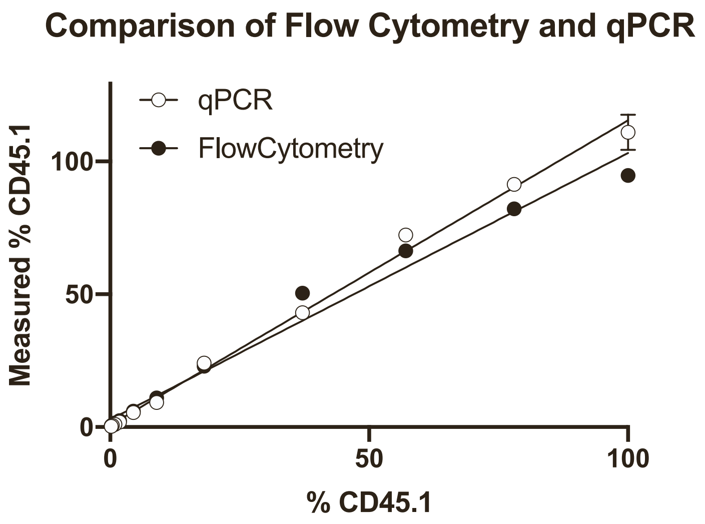

	qPCR	FlowCytometry
Goodness of Fit		
R squared	0.9958	0.9810

	qPCR	FlowCytometry
95% Confidence Intervals		
Slope	1.091 to 1.204	0.8984 to 1.109
Y-intercept	-1.744 to 3.201	-1.824 to 7.415
X-intercept	-2.882 to 1.475	-7.959 to 1.705

Figure S10. Validation of qPCR for the measurement of chimerism in brain and liver
(A) Whole bone marrows from CD45.1 and CD45.2 mice were resuspended at the same cellular density and mixed at the following ratios: 100 % (CD45.1 only); 80 % CD45.1 + 20 % CD45.2; 60 % CD45.1 + 40 % CD45.2; 40 % CD45.1 + 60 % CD45.2; 20 % CD45.1 + 80 % CD45.2; 10 % CD45.1 + 90 % CD45.2; 5 % CD45.1 + 95 % CD45.2; 2 % CD45.1 + 98 % CD45.2; 1 % CD45.1 + 99 % CD45.2; 0.5 % CD45.1 + 99.5 % CD45.2; 0.25 % CD45.1 + 99.75 % CD45.2. Each dilution was divided in 2 for flow cytometry and qPCR analysis. (B) Chimerism analysis of different CD45.1/CD45.2 bone marrow ratios samples measured by flow cytometry or allele specific qPCR on the Cd45.1 locus. 20 000 events were collected for flow cytometry analysis, while qPCR analysis was performed in technical triplicates. Data are presented as means ± SEM and were analyzed by one-way ANOVA with Bonferroni's correction.

Table S1 - Best fit & Confidence Intervals Values

Figure	Description of the experiment	Parameter	Description of the Parameter	Condition				
S1 G	Intracellular Specific Activity	Slope (nmol/(mg*a.u.)*4hrs)		***IDSco***	***IDS.IGF2co***	***IDS.ApoE2co***	***IDS.RAP12x2co***	***GFP***
			Best-fit values	0.6508	0.5176	0.6439	0.5255	N/A
			95% Confidence Intervals	-0.6291 to 1.931	-2.376 to 3.412	-0.3614 to 1.649	-0.6748 to 1.726	N/A
S1 H	Specific Activity in Medium	Slope (nmol/(ml*a.u.)*4hrs)		***IDSco***	***IDS.IGF2co***	***IDS.ApoE2***	***IDS.RAP12x2***	***GFP***
			Best-fit values	0.5165	0.5758	1.04	0.7757	N/A
			95% Confidence Intervals	-1.303 to 2.336	-0.8851 to 2.037	0.9373 to 1.143	-1.825 to 3.376	N/A
1 G	uptake into bEND.3 cells	EC50 Ratio IDS/Tag (a.u.)		***IDSco***	***IDS.IGF2co***	***IDS.ApoE2co***	***IDS.RAP12x2co***	***GFP***
			Best-fit values	1	0.2225	0.6666	0.983	5.168
			95% Confidence Intervals	N/A	0.1914 to 0.2537	0.5807 to 0.7545	0.8430 to 1.145	2.722 to ???
1 H	uptake into MPS II fibroblasts	EC50 Ratio IDS/Tag (a.u.)		***IDSco***	***IDS.IGF2co***	***IDS.ApoE2***	***IDS.RAP12x2***	***GFP***
			Best-fit values	1	0.2037	0.675	1.355	Unstable
			95% Confidence Intervals	N/A	0.1809 to 0.2269	0.6124 to 0.7384	1.205 to 1.537	(Very wide)
2 D	Activity in Bone Marrow vs VCN in Bone Marrow	Max IDS Activity (nmol/mg*4hrs)		***IDSco***	***IDS.IGF2co***	***IDS.ApoE2co***	***IDS.RAP12x2co***	***GFP***
			Best-fit values	4779	5064	6505	7090	N/A
			95% Confidence Intervals	3739 to 6420	4058 to 6977	4232 to 19028	4363 to 29549	N/A
		VCN 50% Max IDS Activity (VCN)		***IDSco***	***IDS.IGF2co***	***IDS.ApoE2co***	***IDS.RAP12x2co***	***GFP***
			Best-fit values	0.2772	0.8033	1.524	1.363	N/A
			95% Confidence Intervals	??? to 0.9174	0.2670 to 2.218	0.3340 to 11.42	0.2476 to 15.60	N/A
2 F	Activity in Plasma vs VCN in Bone Marrow	Slope (nmol/(mg*VCN)*4hrs)		***IDSco***	***IDS.IGF2co***	***IDS.ApoE2***	***IDS.RAP12x2***	***GFP***
			Best-fit values	29.15	11.88	29.75	30.08	N/A
			95% Confidence Intervals	13.51 to 44.80	8.127 to 15.63	19.60 to 39.90	13.87 to 46.29	N/A
3 C	Brain Heparan Sulfate vs VCN in Bone Marrow	Plateau (µg/mg HS)		***IDSco***	***IDS.IGF2co***	***IDS.ApoE2co***	***IDS.RAP12x2co***	***GFP***
			Best-fit values	1.161	0.4629	0.2153	1.253	N/A
			95% Confidence Intervals	1.060 to 1.257	0.3128 to 0.6098	0.1333 to 0.2971	0.8928 to 1.603	N/A
		λ, Exponential decay constant (1/VCN)		***IDSco***	***IDS.IGF2co***	***IDS.ApoE2co***	***IDS.RAP12x2co***	***GFP***
			Best-fit values	4.387	6.264	11.61	6.432	N/A
			95% Confidence Intervals	3.018 to ???	4.013 to 11.36	8.286 to ???	3.054 to 25.43	N/A
3 D	Brain Heparan Sulfate vs IDS activity in brain	Plateau (µg/mg HS)		***IDSco***	***IDS.IGF2co***	***IDS.ApoE2***	***IDS.RAP12x2***	***GFP***
			Best-fit values	1.078	0.4228	0.211	1.106	N/A
			95% Confidence Intervals	0.9645 to 1.177	0.3190 to 0.5262	0.1225 to 0.2949	0.5507 to 1.476	N/A
		λ, Exponential decay constant (1/(nmol/mg*4hrs))		***IDSco***	***IDS.IGF2co***	***IDS.ApoE2***	***IDS.RAP12x2***	***GFP***
			Best-fit values	0.3263	0.5828	1.157	0.6017	N/A
			95% Confidence Intervals	0.2601 to 0.4380	0.4762 to 0.7351	0.8118 to ???	0.3183 to 1.430	N/A
7 I	Functional ELISA Cluster IV LRP-1	Calculated Vmax (a.u)		***IDSco***	***IDS.IGF2co***	***IDS.ApoE2co***	***IDS.RAP12x2co***	***GFP***
			Best-fit values	-11.08	N/A	115.6	185.2	N/A
			95% Confidence Intervals	-infinity to ???	N/A	100.4 to 133.9	-45.25 to +infinity	N/A
		Calculated kd (ng/ml)		***IDSco***	***IDS.IGF2co***	***IDS.ApoE2co***	***IDS.RAP12x2co***	***GFP***
			Best-fit values	18155	N/A	319.2	23930	N/A
			95% Confidence Intervals	-infinity to +infinity	N/A	196.7 to 509.2	-9236 to +infinity	N/A
7 J	Functional ELISA Domain 11 CI-M6P/IGF2R	Calculated Vmax (a.u)		***IDSco***	***IDS.IGF2co***	***IDS.ApoE2***	***IDS.RAP12x2***	***GFP***
			Best-fit values	9.075	122	N/A	N/A	N/A
			95% Confidence Intervals	??? to 12.14	104.8 to 144.5	N/A	N/A	N/A
		Calculated kd (ng/ml)		***IDSco***	***IDS.IGF2co***	***IDS.ApoE2***	***IDS.RAP12x2***	***GFP***
			Best-fit values	1.879	225.7	N/A	N/A	N/A
			95% Confidence Intervals	??? to 9.096	145.0 to 376.4	N/A	N/A	N/A
7 K	Brain Heparan Sulfate vs IDS activity in plasma	Plateau (µg/mg HS)		***IDSco***	***IDS.IGF2co***	***IDS.ApoE2co***	***IDS.RAP12x2co***	***GFP***
			Best-fit values	1.073	0.3452	0.1975	1.149	N/A
			95% Confidence Intervals	0.9062 to 1.203	0.3052 to 0.3849	0.1095 to 0.2829	0.4975 to 1.549	N/A
		λ, Exponential decay constant (1/(nmol/mg*4hrs))		***IDSco***	***IDS.IGF2co***	***IDS.ApoE2co***	***IDS.RAP12x2co***	***GFP***
			Best-fit values	0.03096	0.0869	0.0525	0.03132	N/A
			95% Confidence Intervals	0.02293 to 0.04954	0.08168 to 0.09262	0.03942 to ???	0.01457 to 0.09530	N/A
S8 A	VCN in bone marrow vs VCN in Brain/VCN in Liver	Slope (a.u.)		**Brain**		**Liver**		
			Best-fit values	0.00264		0.04911		
			95% Confidence Intervals	0.0008020 to 0.004478		0.03599 to 0.06223		

Table S2 - Alcian Blue Scoring Rules for Meninges and Choroid Plexus

Scoring of alcian blue staining in choroid plexus and meninges	
Score	Rules
1	Weak alcian blue staining
2	Mild alcian blue staining
3	Strong alcian blue staining
4	Very strong alcian blue staining
