# Supplementary material for: Tagged IDS causes efficient and engraftment-independent prevention of brain pathology during lentiviral gene therapy for Mucopolysaccharidosis type II
Source: Mol Ther Methods Clin Dev. 2023 Nov 2;31:101149. doi: 10.1016/j.omtm.2023.101149 (PMC10684800; doi:10.1016/j.omtm.2023.101149)
Supplement: Document S2. Article plus supplemental information [file mmc2.pdf]

Molecular Therapy
Methods & Clinical Development
Original Article

Tagged IDS causes efficient and engraftment-independent prevention of brain pathology during lentiviral gene therapy for Mucopolysaccharidosis type II

Fabio Catalano,[1,2,3] Eva C. Vlaar,[1,2,3] Drosos Katsavelis,[1,2,3] Zina Dammou,[1,2,3] Tessa F. Huizer,[1,2,3] Jeroen C. van den Bosch,[1,3] Marianne Hoogeveen-Westerveld,[1,3] Hannerieke J.M.P. van den Hout,[2,3] Esmeralda Oussoren,[2,3] George J.G. Ruijter,[1,3] Gerben Schaaf,[1,2,3] Karin Pike-Overzet,[4] Frank J.T. Staal,[4,5] Ans T. van der Ploeg,[2,3] and W.W.M. Pim Pijnappel[1,2,3]

[1]Department of Clinical Genetics, Erasmus MC University Medical Center, Rotterdam 3015GE, the Netherlands; [2]Department of Pediatrics, Erasmus MC University Medical Center, Rotterdam 3015GE, the Netherlands; [3]Center for Lysosomal and Metabolic Diseases, Erasmus MC University Medical Center, Rotterdam 3015GE, the Netherlands; [4]Department of Immunology, Leiden University Medical Center, Leiden 2333ZA, the Netherlands; [5]Department of Pediatrics, Leiden University Medical Center, Leiden 2333ZA, the Netherlands

Mucopolysaccharidosis type II (OMIM 309900) is a lysosomal storage disorder caused by iduronate 2-sulfatase (IDS) deficiency and accumulation of glycosaminoglycans, leading to progressive neurodegeneration. As intravenously infused enzyme replacement therapy cannot cross the blood-brain barrier (BBB), it fails to treat brain pathology, highlighting the unmet medical need to develop alternative therapies. Here, we test modified versions of hematopoietic stem and progenitor cell (HSPC)-mediated lentiviral gene therapy (LVGT) using IDS tagging in combination with the ubiquitous MND promoter to optimize efficacy in brain and to investigate its mechanism of action. We find that IDS tagging with IGF2 or ApoE2, but not RAP12x2, improves correction of brain heparan sulfate and neuroinflammation at clinically relevant vector copy numbers. HSPC-derived cells engrafted in brain show efficiencies highest in perivascular areas, lower in choroid plexus and meninges, and lowest in parenchyma. Importantly, the efficacy of correction was independent of the number of brain-engrafted cells. These results indicate that tagged versions of IDS can outperform untagged IDS in HSPC-LVGT for the correction of brain pathology in MPS II, and they imply both cell-mediated and tag-mediated correction mechanisms, including passage across the BBB and increased uptake, highlighting their potential for clinical translation.

INTRODUCTION

When first reported in 1917 by Charles Hunter, Mucopolysaccharidosis type II (MPS II) or Hunter syndrome (OMIM 309900) was hypothesized to result from congenital defects of development.[1] It was not until 1972 that the causing factor was identified as a deficiency of iduronate 2-sulfatase (IDS), a lysosomal enzyme involved in the stepwise degradation of the glycosaminoglycans (GAG) heparan sulfate (HS) and dermatan sulfate (DS).[2–4] Disease-associated variants of the *IDS* gene (HGNC ID: 5389) are inherited in an X-linked manner, causing MPS II in males and in rare cases in females depending on the pattern of X-inactivation (global incidence of ~0.7 per 100,000 living newborns).[5] Progressive neuronal abnormalities, gliosis, and demyelination are among the pathological manifestations of the neuronopathic form of MPS II, which affects approximately two-thirds to ~three-fourths of the total MPS II population. These patients experience a more severe disease progression and a shortened life expectancy of only up to 20 years of age compared with non-neuronopathic patients, who can survive until late adulthood.

As large molecules cannot penetrate the blood-brain barrier (BBB), intravenous enzyme replacement therapy (ERT) with recombinant human idursulfase (Elaprase) was found not to improve CNS symptoms in long-term studies with more than 9 years follow-up.[6,7] Since then, efforts focused on the development of alternative therapies able to treat the neurological symptoms of MPS II patients, including modified versions of ERT[8,9] and gene therapy. Among these, gene therapy has the potential to provide long-term treatment following a single intervention.[10–12] An example is *ex vivo* lentiviral transduction of autologous hematopoietic stem and progenitor cells (HSPCs), followed by a standard HSPC transplantation. This approach, referred to as HSPC-mediated lentiviral gene therapy (LVGT), combines an *ex vivo* transduction of stem cells with the ability of lentiviral vectors to stably integrate into the host genome. A number of clinical trials with more than 10 years

Received 28 June 2023; accepted 31 October 2023;
https://doi.org/10.1016/j.omtm.2023.101149.

Correspondence: W.W.M. Pim Pijnappel, Erasmus University Medical Center, 3015GE Rotterdam, the Netherlands.
E-mail: w.pijnappel@erasmusmc.nl

This is an open access article under the CC BY license (http://creativecommons.org/licenses/by/4.0/).

follow-up proved the safety of this approach[13–17] and, importantly, its potential in the treatment of neurological disorders.[13,16] The latter is classically explained by the well-documented brain engraftment of HSPC-derived cells following the preconditioning procedures.[18,19] Once engrafted, HSPC-derived cells differentiate into microglia-like cells, and serve as an *in loco* source of missing enzyme, aiding in the prevention of pathology.

HSPC-LVGT has been tested for MPS II in few preclinical studies. In most cases, codon-optimized human *IDS* was placed under the control of the MND (myeloproliferative sarcoma virus enhancer, negative control region deleted, dl587rev primer-binding site substituted)-derived MCU3 promoter, a strong ubiquitous promoter that resulted in an increase of IDS activity in bone marrow and plasma of 30- to 296-fold above activity in wild-type (WT) animals.[20–22] Nevertheless, the use of unmodified IDS in these studies caused only a partial reduction of GAG levels and pathology in the CNS. To improve the efficacy in brain, Gleitz and co-workers used human IDS-tagged C-terminally with ApoE2, an epitope tag derived from apolipoprotein E and able to undergo transcytosis across the BBB.[23] This resulted in near-complete normalization of cerebral GAG levels and neuroinflammation after gene therapy with IDS.ApoE2, while gene therapy with untagged IDS only partially reduced CNS pathology. The transgenes were expressed under the myeloid-specific promoter CD11b, which resulted in IDS overexpression in bone marrow and plasma at levels 3- to 10-fold above healthy mice, well below the levels reached by others with the MCU3 promoter.

Here, we combined high transgene expression driven by the strong ubiquitous MND[17,24] promoter with fusion of IDS to different peptides. In a first experiment, we compared lentiviral vectors encoding IDS or insulin-like growth factor 2 (IGF2)-tagged IDS in a dose-response analysis. We previously showed that IGF2 tagging of acid alpha-glucosidase (GAA) strongly improved the efficacy of HSPC-LVGT in a mouse model for Pompe disease. Among the corrected tissues, brain was the best responding one, showing correction of pathological glycogen accumulation already at a low dose of lentiviral gene therapy with IGF2.GAA, while HSPC-LVGT with untagged GAA failed to correct the brain even at high doses.[25] In a second experiment of the present study, we compared lentiviral vectors encoding the previously reported IDS.ApoE2,[23] as well as IDS tagged with a tandem repeat of the receptor-associated protein (RAP) minimal peptide (RAP12x2), an epitope able to undergo transcytosis across the BBB.[26]

We found that, upon HSPC-LVGT with untagged IDS, correction of brain pathology was partial and restricted to those areas where engraftment of donor-derived cells was observed. IDS-tagging with IGF2 and ApoE2, but not with RAP12x2, improved correction of cerebral HS accumulation and pathology via mechanisms that were independent of the extent of engraftment of donor-derived cells. This may involve tag-mediated mechanisms—such as blood-to-brain transport and/or cross-correction via enhanced uptake—that are not necessarily the same for the IGF2 and ApoE2 tags. These results highlight the potential of tagging lysosomal proteins to enhance the efficacy of HSPC-LVGT in the treatment of neuronopathic lysosomal storage disorders (LSDs), and identify *IDS.IGF2co*, in addition to *IDS.ApoE2co* in combination with the MND promoter, as promising new vectors for the correction of brain pathology during HSPC-LVGT for Hunter disease.

RESULTS

Tag-dependent effects of C-terminal tagging of IDS on processing, secretion, specific activity, and uptake

Codon-optimized tag sequences encoding either IGF2[25] (human insulin-like growth factor 2; amino acid [aa]: 1, 8–67), ApoE2[27] (human apolipoprotein E; aa: 141–149 in a double tandem repeat), or RAP12x2[26] (human receptor-associated protein; aa: 151–262 in a double repeat separated by a Gly residue) were fused C-terminally to a codon-optimized sequence encoding human IDS using a linker sequence consisting of Leu(Gly-Gly-Gly-Gly-Ser) x 4. The resulting sequences were cloned into third-generation self-inactivating (SIN) lentiviral vectors (Figure 1A) under the control of the MND (myeloproliferative sarcoma virus enhancer, negative control region deleted, dl587rev primer-binding site substituted) promoter.[28]

To assess the effect of tagging on IDS protein expression and processing, we transiently transfected HEK293T cells with the lentiviral vectors shown in Figure 1A. Human IDS is synthesized as precursor protein with an apparent molecular weight (MW) of 76 kDa and it is further processed by proteolytic cleavage into mature forms with apparent MWs of 55 and 45 kDa.[29] SDS-PAGE followed by immunoblot analysis at day 4 after transfection revealed the presence of a precursor protein at an apparent MW of 76 kDa after transfection with *IDSco* and *IDS.ApoE2co*, while slightly higher MW precursors were visible after transfection with *IDS.IGF2co* (∼84 kDa) and *IDS.RAP12x2co* (∼81 kDa) (Figure 1B, total protein load is shown in Figure S1A) (theoretical MWs of precursor proteins: IDS = 61.87 kDa; IDS.IGF2 = 69.97 kDa; IDS.ApoE2 = 65.60 kDa; IDS.RAP12x2 = 66.32 kDa). All tagged versions of IDS were successfully processed into the 55 and 45 kDa mature forms, suggesting that the tag is cleaved off upon lysosomal sorting (Figures 1B, 1C, and S1B; values in Figures 1C and S1B are quantified within the linear range of detection shown in Figure S1C and corrected for total protein and transfection efficiency shown in Figures S1A and S1D, respectively). IDS and IDS.IGF2 had a similar processing pattern (Figures 1C and S1B), while IDS.ApoE2 showed a higher amount of the 55 and 45 kDa mature forms compared with the *IDSco* (Figures 1C and S1B). IDS.RAP12x2 was mainly present as the 76 kDa form and showed reduced levels of the 45 and 55 kDa mature forms compared with the other tagged IDS versions (Figures 1C and S1B). Relative IDS enzyme activity in cells for IDS, IDS.IGF2, and IDS.ApoE2 matched intracellular IDS protein levels, except for IDS.RAP12x2, which showed lower enzyme activity levels despite higher protein levels relative to the other tagged IDS proteins (compare Figures 1C and 1D; values in Figure 1D are normalized for transfection efficiency shown in Figure S1D).

To evaluate differences in secretion, we measured IDS levels in medium over 4 days after transient transfection of HEK293T cells

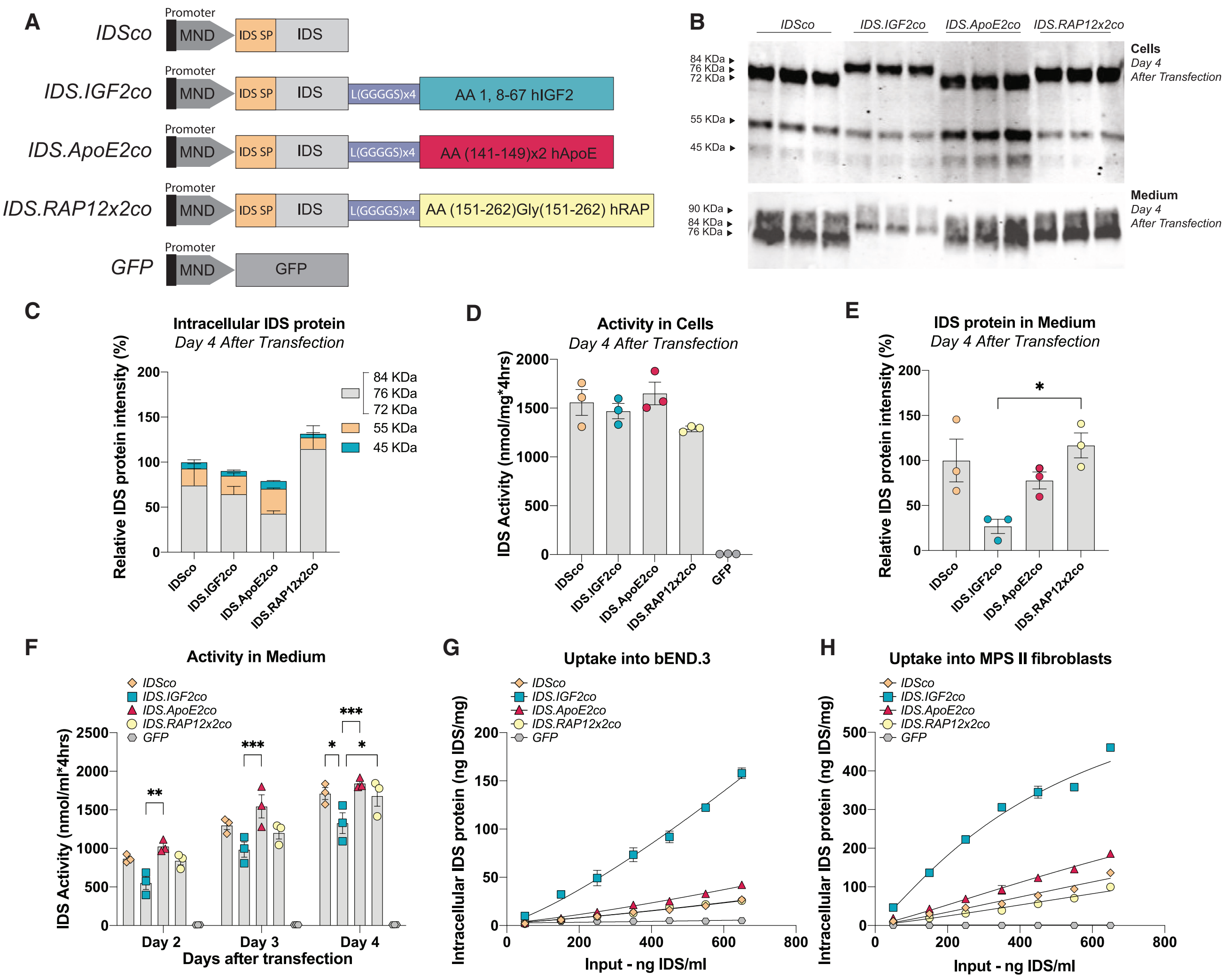

Figure 1. *In vitro* characterization of tagged IDS proteins

(A) Cartoons of pCCL third-generation lentiviral vectors encoding codon optimized (co) human IDS proteins under the control of the MND promoter. Human IDS was either not tagged or tagged C-terminally with IGF2 (*IDS.IGF2co*), ApoE2 (*IDS.ApoE2*), or RAP12x2 (*IDS.RAP12x2co*). An identical lentiviral vector encoding GFP served as control. IDS SP, IDS signal peptide; hIGF2, human insulin-like growth factor 2; hApoE, human apolipoprotein E; hRAP, human receptor-associated protein. (B–F) HEK293T cells were transfected with lentiviral vectors shown in (A). (B) Immunoblot analysis using an anti-IDS antibody of cell lysate and medium at day 4 after transfection. Three biological replicas are shown. Quantification of (B) is shown in (C and E). Protein loading was determined by quantification of the stain-free signal (Figure S1A) and was used for normalization. (D) Intracellular IDS enzyme activity at day 4 after transfection. (F) IDS enzyme activity in medium at days 2, 3, and 4 after transfection. Data in (C)–(F) were normalized for transfection efficiency (Figure S1D). Captured IDS protein using IDS sandwich ELISA after 24 h uptake into bEND.3 cells (G) and MPS II fibroblasts (H). Data in (C), (E), (G), and (H) were quantified within the IDS antibody linear range (Figure S1C). Data represent means ± SEM and were analyzed by one-way ANOVA followed by Bonferroni's multiple testing correction. n = 3 biological replicates/condition. $^{*}p \leq 0.05$, $^{**}p \leq 0.01$, $^{***}p \leq 0.001$. Significant comparisons are indicated by brackets.

(Figures 1B, 1E, and 1F; values in Figure 1E are normalized for total protein shown in Figure S1A and for transfection efficiency shown in Figure S1D, while values in Figure 1F are normalized for transfection efficiency shown in Figure S1D). At day 4, immunoblot analysis using an IDS antibody revealed that IDS and tagged IDS versions were secreted as a precursor protein of the same apparent MW as the intracellular precursor protein, in addition to other precursor forms ranging from 72 to 90 kDa (Figure 1B).[29] Immunoblot quantification showed comparable IDS protein levels in medium for IDS, IDS.ApoE2, and IDS.RAP12x2, while IDS.IGF2 levels were lower (Figures 1B and 1E). In agreement, medium IDS enzyme activity levels of IDS.IGF2 were lower compared with the other IDS proteins already at day 2 after transfection, and continued to be lower until day 4 (Figure 1F). Addition of excess recombinant IGF2 peptide (1.5 μM) in the medium after transient transfection of HEK293T cells with *IDS.IGF2co* resulted in higher IDS activity levels in the medium—but not in cells—compared with medium from cells transfected with *IDS.IGF2co* without IGF2 peptide addition (Figure S1E), suggesting that

IGF2-mediated reuptake of secreted IDS.IGF2 contributes to the reduced levels of IDS.IGF2 protein and activity in medium.

To assess the effect of tagging on IDS enzyme activity, IDS protein levels and enzyme activity levels were measured in three 2-fold dilutions of cell lysate and medium of transfected HEK293T cells (Figures S1F–S1H). By plotting protein levels vs. IDS enzyme activity levels in cell lysates and medium, we observed similar slopes for all versions of IDS (Figures S1G and S1H), with small differences that were not significant (Table S1). The calculated specific activity in cells relative to the untagged IDS protein (set to 100%) for IDS.IGF2, IDS.ApoE2, and IDS.RAP12x2 was as follows: 114.72% (SD 25.47%), 103.09% (SD 3.28%), and 74.92% (SD 4.52%), respectively. In medium, the calculated relative specific activities were 131.40% (SD 11.21%), 135.96% (SD 17.45%), and 114.24% (SD 13.77%) for IDS.IGF2, IDS.ApoE2, and IDS.RAP12x2 relative to the untagged IDS protein (100%), respectively.

Next, conditioned medium from transfected HEK293T cells was applied to the mouse brain endothelial cell line bEND.3 and to primary human MPS II fibroblasts to compare uptake of IDS proteins. Conditioned medium from *GFP*-transfected HEK293T cells was used as negative control (Figures 1G and 1H). In both bEND.3 cells and MPS II fibroblasts, IDS.IGF2 showed a ~5-fold higher uptake compared with untagged IDS across the dilution range tested, with EC_{50} values that were 5 times lower compared with IDS ($EC_{50ratio}$ bEND.3 cell: IDS = 1 a.u.; IDS:IGF2: 0.223 a.u.; $EC_{50ratio}$ MPS II fibroblasts: IDS = 1 a.u.; IDS:IGF2: 0.204 a.u.; Table S1). The ApoE2 tag provided a modest enhancement of uptake in both cell types, with EC_{50} values that were 1.5 times smaller compared with untagged IDS ($EC_{50ratio}$ bEND.3 cells: IDS = 1 a.u.; IDS:ApoE2: 0.666 a.u.; $EC_{50ratio}$ MPS II fibroblasts: IDS = 1 a.u.; IDS:ApoE2: 0.675 a.u.; Table S1). In bEND.3 cells, uptake of IDS and IDS.RAP12x2 were indistinguishable, while the RAP12x2 tag caused an increase of 1.35-fold of the EC_{50} values in MPS II fibroblasts, resulting in reduced uptake compared with untagged IDS ($EC_{50ratio}$ bEND.3 cells: IDS = 1 a.u.; IDS:RAP12x2: 0.983 a.u.; $EC_{50ratio}$ MPS II fibroblasts: IDS = 1 a.u.; IDS:RAP12x2: 1.355 a.u.; Table S1).

These results show that the tagging strategy of IDS adopted here has a differential effect on uptake depending on the tag used, and results in minor effects on specific activity that were not significant. The fusion proteins tested were functional to different extents: IGF2 and ApoE2 tags caused an improvement of 5 and 1.5 times of the uptake in the cell lines tested, respectively, without causing detrimental effects on specific activity and processing, while tagging with RAP12x2 had a negative effect on intracellular specific activity and showed no improvement of cellular uptake *in vitro*.

Gene therapy results in supraphysiological IDS activity levels in bone marrow and plasma

Gene therapy was performed in two rounds of transplantation using an established *Ids*$^{y/-}$ mouse model for MPS II.[30,31] In the first round, we tested *IDSco* and *IDS.IGF2co* in a dose-response analysis by varying the lentiviral vector dose (MOI = 0.1, 1, and 3). In the second round, we compared *IDSco*, *IDS.IGF2co*, *IDS.ApoE2co*, and *IDS.RAP12x2co* at a single dose of lentiviral gene therapy (MOI = 1; Figure 2). We transplanted lentiviral-transduced HSPCs into 2-month-old irradiated *Ids*$^{y/-}$ mice and analyzed correction of brain pathology 6 months after transplantation. Vector copy number (VCN) in bone marrow ranged between 0.5 and 4 depending on the lentiviral dose applied (Figure 2A). Chimerism in bone marrow was ~85% for all the conditions, indicating efficient engraftment of HSPCs (Figures 2B and S2A).

Gene therapy resulted in supraphysiological levels of IDS enzyme activity in bone marrow (~150-fold over WT) for all the treatment groups compared with the *GFP* groups and untreated *Ids*$^{y/-}$ mice (Figure 2C). IDS activity levels in bone marrow varied according to VCN in bone marrow and followed a hyperbolic function with a maximal IDS activity at a VCN between 2 and 4 for all treatment groups (Figure 2D; Table S1). In agreement, supraphysiological levels of IDS activity were detected in plasma after lentiviral gene therapy (*IDSco*, *IDS.ApoE2co*, *IDS.RAP12x2co* > 60-fold over WT; *IDS.IGF2co* > 30-fold over WT; Figure 2E). Plasma IDS activity levels increased linearly with the bone marrow VCN, with the *IDS.IGF2co* treatment showing a more modest slope compared with the other treatments, which may be explained by reduced secretion and/or stability of IDS.IGF2 protein, or increased elimination rate from plasma compared with the other IDS proteins (Figure 2F; Table S1). Plasma IDS activity levels correlated with IDS protein levels in plasma as shown by immunoblot analysis (Figure S2B; levels in Figure S2B were normalized for total protein shown in Figure S2C). These results demonstrate high expression of all the vectors tested, resulting in supraphysiological IDS activity levels in bone marrow and plasma at low VCNs.

IGF2 and ApoE2 tagging of IDS result in superior correction of brain pathology

Gene therapy resulted in partial restoration of IDS enzyme activity in brains of *Ids*$^{y/-}$ mice (Figure 3A). In general, IDS enzyme activity levels were ~10- to 30-fold lower compared with untreated WT mice and varied according to the lentiviral vector dose administrated, with similar levels for *IDSco*, *IDS.IGF2co*, *IDS.ApoE2co*, and *IDS.RAP12x2co*.

Untreated and *GFP*-treated *Ids*$^{y/-}$ mice showed ~70-fold higher HS levels in brain lysates compared with untreated WT mice (Figure 3B), but not significantly higher levels of DS (Figure S3).[32] Gene therapy with *IDSco* resulted in a ~3-fold reduction of brain HS levels, but failed to show a dose-dependent effect (Figure 3B, experiment 1). In contrast, *IDS.IGF2co* caused a dose-dependent reduction of brain HS levels (Figure 3B, experiment 1): low dose gene therapy with *IDS.IGF2co* (MOI = 0.1) was able to reduce brain HS to levels that were comparable with *IDSco* MOI 3 treatment, while higher doses of *IDS.IGF2co* resulted in a further reduction of brain HS to levels that were ~3-fold lower than *IDSco* MOI 3 and ~11-fold lower than untreated *Ids*$^{y/-}$ animals. The second set of experiments confirmed the previous finding for *IDSco* and *IDS.IGF2co* vectors, with *IDS.IGF2co* being ~3 times more effective than *IDSco* in reducing HS accumulation in brain at an MOI of 1 (Figure 3B, experiment 2). After gene therapy with *IDS.ApoE2co*, the extent of HS reduction was comparable with gene therapy with *IDS.IGF2co*, although *IDS.ApoE2co*

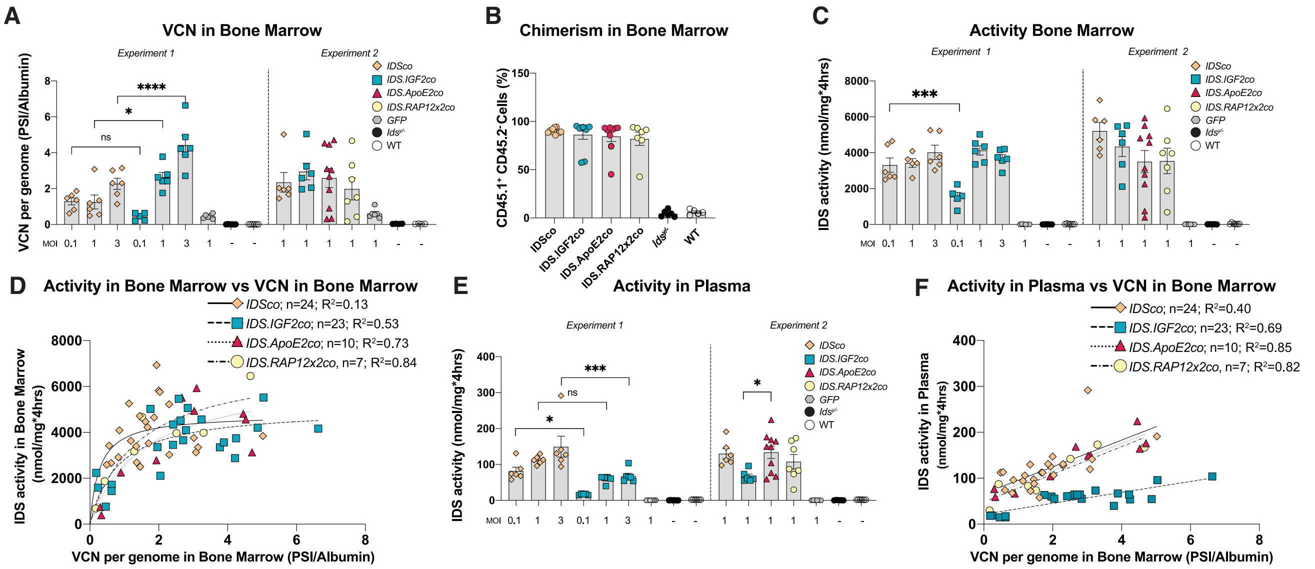

Figure 2. Supraphysiological IDS activity levels after gene therapy

Two-month-old *Ids*$^{y/-}$ mice were treated with gene therapy using 9 Gy total body irradiation (TBI) and the indicated multiplicity of infection (MOI). Gene therapy was performed in two separate experiments, as indicated. (A) VCN per genome measured in bone marrow by qPCR on *PSI* and *Albumin* loci. (B) Chimerism in bone marrow measured by flow cytometry and expressed as percentage of CD45.1$^+$/CD45.2$^-$ cells. For *IDSco* and *IDS.IGF2co*, MOI 1 groups from experiments 1 and 2 were combined in the same graph. (C) IDS enzyme activity in bone marrow. (D) Non-linear regression analysis between vector copy number and IDS enzyme activity in bone marrow. (E) IDS enzyme activity in plasma. (F) Linear regression analysis between vector copy number in bone marrow and IDS enzyme activity in plasma. Regression analysis of (D and F) is shown in Table S1. Data represent means ± SEM and were analyzed by one-way ANOVA followed by Bonferroni's multiple testing correction. Experiment 1: n = 5 or 6 per group. Experiment 2: *IDSco*, *IDS.IGF2co*, *GFP*, *Ids*$^{y/-}$ and WT n = 6; *IDS.ApoE2co* n = 10; *IDS.RAP12x2co* n = 7. *p ≤ 0.05, ***p ≤ 0.001, ****p ≤ 0.0001. Significant results are indicated by brackets.

resulted in marginally lower HS levels (~11- and ~15-fold reduction vs. *Ids*$^{y/-}$ mice, respectively). HS levels in brain homogenates of mice treated with *IDS.RAP12x2co* gene therapy were similar to the levels detected after gene therapy with the *IDSco* vector. Importantly, none of the vectors resulted in complete normalization of brain HS levels under the conditions employed. The best outcomes were achieved by the *IDS.IGF2co* and *IDS.ApoE2co* vectors with 8- and 5-fold higher HS levels than WT animals, respectively (WT: 0.06 ± 0.004 μg/mg; *Ids*$^{y/-}$ 3.80 ± 0.28 μg/mg; *GFP*: 3.78 ± 0.16 μg/mg; *IDSco* MOI 1: 1.20 ± 0.23 μg/mg; *IDS.IGF2co* MOI 1: 0.39 ± 0.08 μg/mg; *IDS.ApoE2co* MOI 1: 0.24 ± 0.10 μg/mg; *IDS.RAP12x2co* MOI 1: 1.41 ± 0.45 μg/mg). Brain HS was reduced with the VCN in bone marrow following a negative exponential curve that reached a plateau for all vectors (Figure 3C; Table S1). Treatment with *IDSco* and *IDS.RAP12x2co* resulted in comparable plateau values of ~1.20 μg/mg cerebral HS, while *IDS.IGF2co* and *IDS.ApoE2co* plateaued at 0.46 μg/mg and 0.22 μg/mg brain HS, respectively. The exponential decay constant (λ) was not significantly different after gene therapy with the vectors tested (Table S1), although *IDS.ApoE2co* caused a steeper decrease of brain HS with VCN compared with the other vectors, reaching plateau values at VCN as low as 0.3 (Figure 3C). Correction of brain HS also followed an exponential decay curve when plotted against IDS activity in brain (Figure 3D). Plateau values after *IDSco* and *IDS.RAP12x2co* treatments were comparable at ~1.10 μg/mg HS. *IDS.IGF2co* plateaued at around 0.43 μg/mg HS, while *IDS.ApoE2co* showed a plateau value of 0.21 μg/mg HS (Table S1).

Alcian blue staining of *Ids*$^{y/-}$ brains showed a widespread accumulation of sulfated mucins in perivascular cells, parenchymal cells, meningeal cells, and in the choroid plexus (Figures 3E and S4), as described previously.[31] Quantification of Alcian blue-positive cells in brain after gene therapy at MOI of 1 showed complete normalization at all brain regions analyzed for *IDS.IGF2co* and *IDS.ApoE2co* (Figures 3F and S4). *IDSco*- and *IDS.RAP12x2co*-treated animals showed marginally higher levels of Alcian blue-positive perivascular cells and parenchymal cells in midbrain, brainstem, and cerebellum compared with WT mice (Figures 3F and S4). Scoring of Alcian blue staining in choroid plexus and meninges showed full correction without differences between the vectors tested (Figures 3G and S4; Table S2).[12]

These data demonstrate that gene therapy reduced HS accumulation and Alcian blue staining in all the cerebral areas analyzed and that tagging of IDS with IGF2 or ApoE2, but not RAP12x2, improved the efficacy of gene therapy. Low VCN values in bone marrow (<2 VCN per genome) were sufficient to reach plateau HS levels, which varied depending on the vector used and resulted in the best outcome with *IDS.IGF2co* and *IDS.ApoE2co* vectors.

Alleviation of lysosomal pathology and neuroinflammation after gene therapy

Ids$^{y/-}$ mice showed a widespread upregulation of LAMP1-positive cells in brain to levels ranging from 16-fold (hypothalamus) to 4-fold (corpus callosum) over WT depending on the brain area

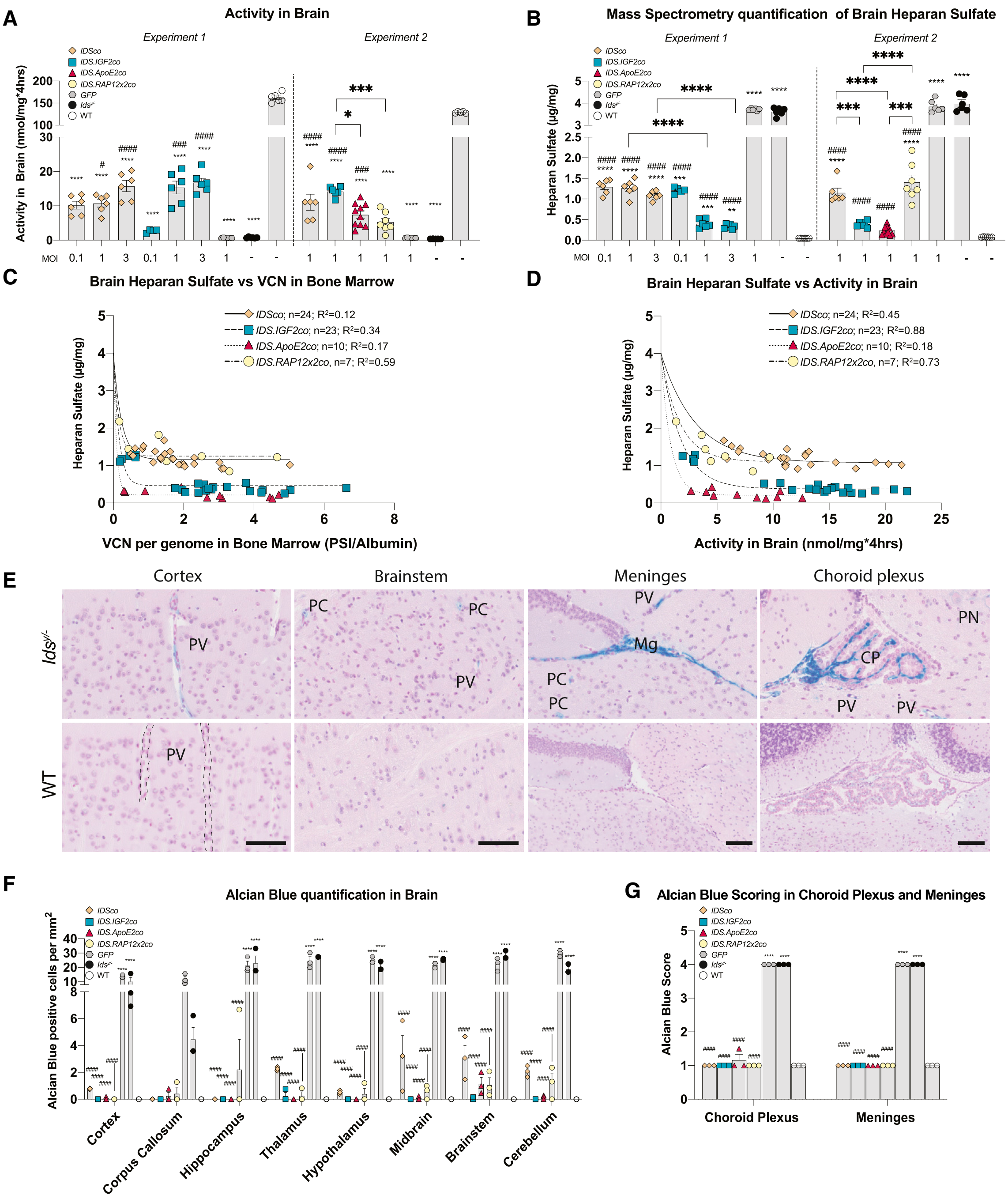

Figure 3. Correction of brain after gene therapy

(A) IDS enzyme activity in brain. (B) Mass spectrometry quantification of total brain heparan sulfate. (C) Relationship between brain heparan sulfate content and vector copy number in bone marrow. (D) Non-linear regression between brain heparan sulfate content and IDS enzyme activity in brain. (E) Representative examples of Alcian blue-positive areas in brain areas of untreated *Ids*$^{y/-}$ mice and matched WT controls. Scale bar, 50 μm. (F) Quantification of Alcian blue staining in different regions of the brain after gene therapy by counting positive cells. Representative examples of Alcian blue staining after gene therapy are shown in Figure S4. (G) Quantification of Alcian blue-positive staining using a scoring system (see Table S2) in choroid plexus and meninges after gene therapy. Data are presented as means ± SEM. In (A) and (B) data were analyzed by one-way ANOVA with Bonferroni's correction. In (F) data were analyzed by two-way ANOVA with Bonferroni's correction, using treatment (*IDSco*, *IDS.IGF2co*, *IDS.ApoE2co*, or *IDS.RAP12x2co*) and brain areas as categorical variables. Regression analysis of (C and D) is shown in Table S1. Asterisks represent significance vs. WT; hashmarks represent significance vs. *Ids*$^{y/-}$. Other significant comparisons are identified by brackets. PV, perivascular cells; PC, parenchymal cell; Mg, meninges; CP, choroid plexus; PN, perineuronal net. (A–D) Experiment 1: n = 5 or 6 per group. Experiment 2: *IDSco*, *IDS.IGF2co*, *GFP*, *Ids*$^{y/-}$ and WT n = 6; *IDS.ApoE2co* n = 10; *IDS.RAP12x2co* n = 7. (F and G) n = 3. *p ≤ 0.05, **p ≤ 0.01, ***p ≤ 0.001, ****p ≤ 0.0001. #p ≤ 0.05, ###p ≤ 0.001, ####p ≤ 0.0001.

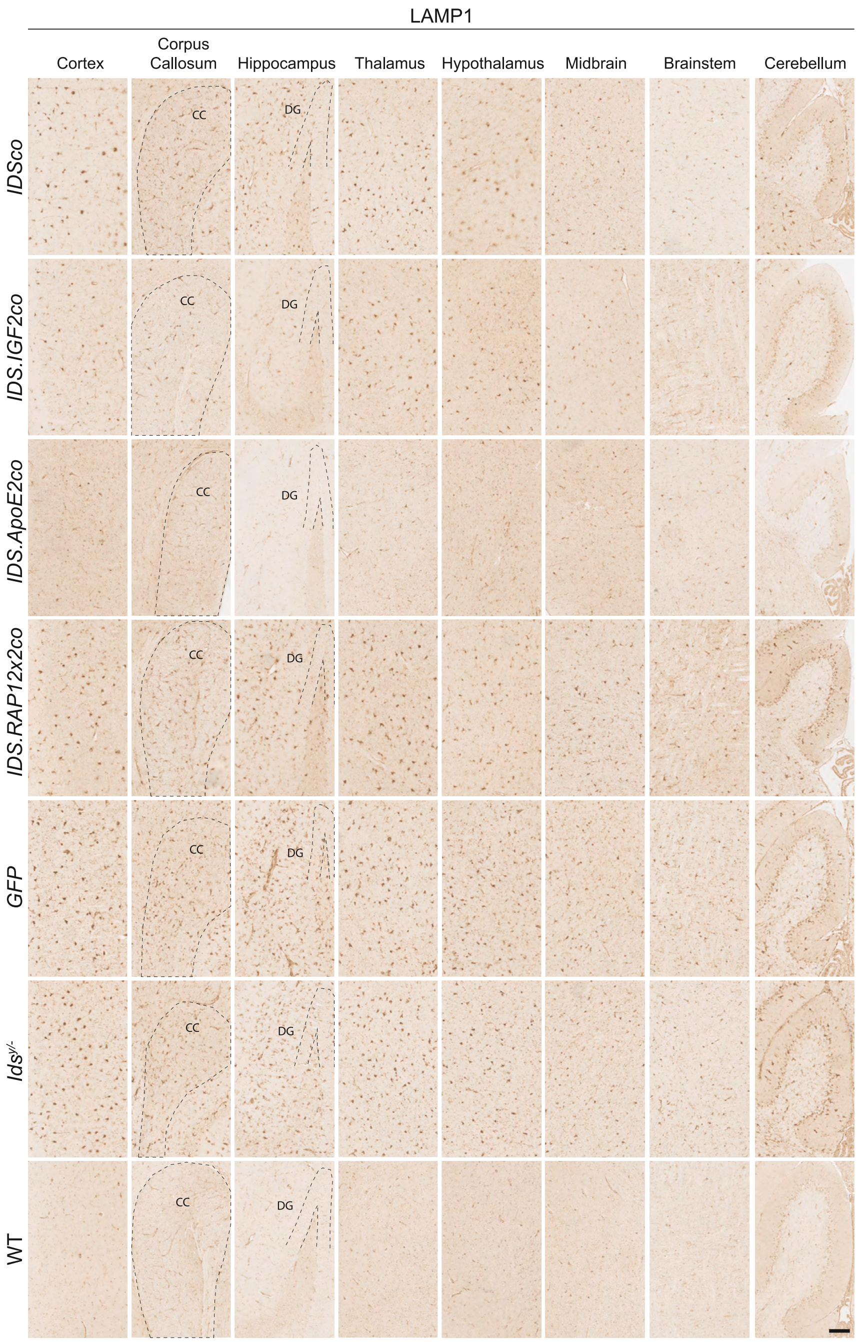

Figure 4. Gene therapy relieves LAMP1 pathology in brain

Representative examples of LAMP1 staining of sagittal sections of cortex, corpus callosum, hippocampus, thalamus, hypothalamus, midbrain, brainstem, and cerebellum of gene therapy-treated *Ids*$^{y/-}$ mice and controls. CC, corpus callosum; DG, dentate gyrus. Scale bar, 100 μm.

analyzed (average increase of 8-fold compared with healthy mice; Figures 4, 6A, and 6B). Gene therapy at an MOI of 1 (see Figure S9 for VCNs of mice used for histology) reduced LAMP1 immunoreactivity for all vectors tested to different extents. *IDSco* and *IDS.RAP12x2co* resulted in an average reduction of LAMP1 levels of 25% and 34% compared with untreated *Ids*$^{y/-}$ mice, respectively. *IDS.RAP12x2co* was marginally more effective than *IDSco* in all the brain areas under analysis, with the highest difference registered in hippocampus (*IDSco*: 13% reduction vs. *Ids*$^{y/-}$; *IDS.RAP12x2co*: 45% reduction vs. *Ids*$^{y/-}$) (Figures 4 and 6B). Gene therapy with *IDS.IGF2co* resulted in a further reduction of LAMP1-positive staining to levels 44% and 36% lower compared with *IDSco* and *IDS.RAP12x2co* treatments, respectively, and 60% lower than untreated *Ids*$^{y/-}$ mice. *IDS.ApoE2co* was slightly better than *IDS.IGF2co*, reducing LAMP1 immunoreactivity to levels 80% lower than *Ids*$^{y/-}$ levels. *IDS.ApoE2co* showed a significantly higher efficacy in specific brain areas, namely in the cortex (*IDS.IGF2co*: 60% reduction vs. *Ids*$^{y/-}$; *IDS.ApoE2co*: 90% reduction vs. *Ids*$^{y/-}$), thalamus (*IDS.IGF2co*: 60% reduction vs. *Ids*$^{y/-}$; *IDS.ApoE2co*: 85% reduction vs. *Ids*$^{y/-}$), and hypothalamus (*IDS.IGF2co*: 52% reduction vs. *Ids*$^{y/-}$; *IDS.ApoE2co*: 84% reduction vs. *Ids*$^{y/-}$). *IDS.ApoE2co* and *IDS.IGF2co* showed similarly high efficacy in corpus callosum, hippocampus, midbrain, brainstem, and cerebellum (Figure 6B).

Next, we analyzed correction of neuroinflammation after gene therapy in *Ids*$^{y/-}$ mice (Figures 5, 6C–6F, and S6). By staining astrocytes for glial fibrillary acid protein (GFAP), we observed an increased number of GFAP-positive cells in *Ids*$^{y/-}$ mice in the cortex (13-fold over WT), thalamus (13-fold over WT), hypothalamus (5-fold over WT), midbrain (13-fold over WT), and brainstem (18-fold over WT), but not in the corpus callosum, hippocampus, and cerebellum, where GFAP immunoreactivity levels ranged from comparable with to marginally lower than WT mice (Figures 6C and 6D). Gene therapy caused changes in GFAP levels depending on the brain region (Figures 5, 6C, 6D, and S5). In cortex, *IDSco* and *IDS.RAP12x2co* vectors reduced GFAP-positive cells by 30% compared with *Ids*$^{y/-}$. *IDS.IGF2co* caused a further reduction in GFAP-positive cells of ∼30% over *IDSco* and *IDS.RAP12x2co* and 60% compared with untreated *Ids*$^{y/-}$ animals. In this area, *IDS.ApoE2co* resulted in 90% reduction of GFAP immunoreactivity, reaching levels comparable with WT animals (*IDS.ApoE2co*: 20 GFAP$^+$ cells/mm^2; WT: 15 GFAP$^+$ cells/mm^2). In thalamus, all the vectors reduced GFAP

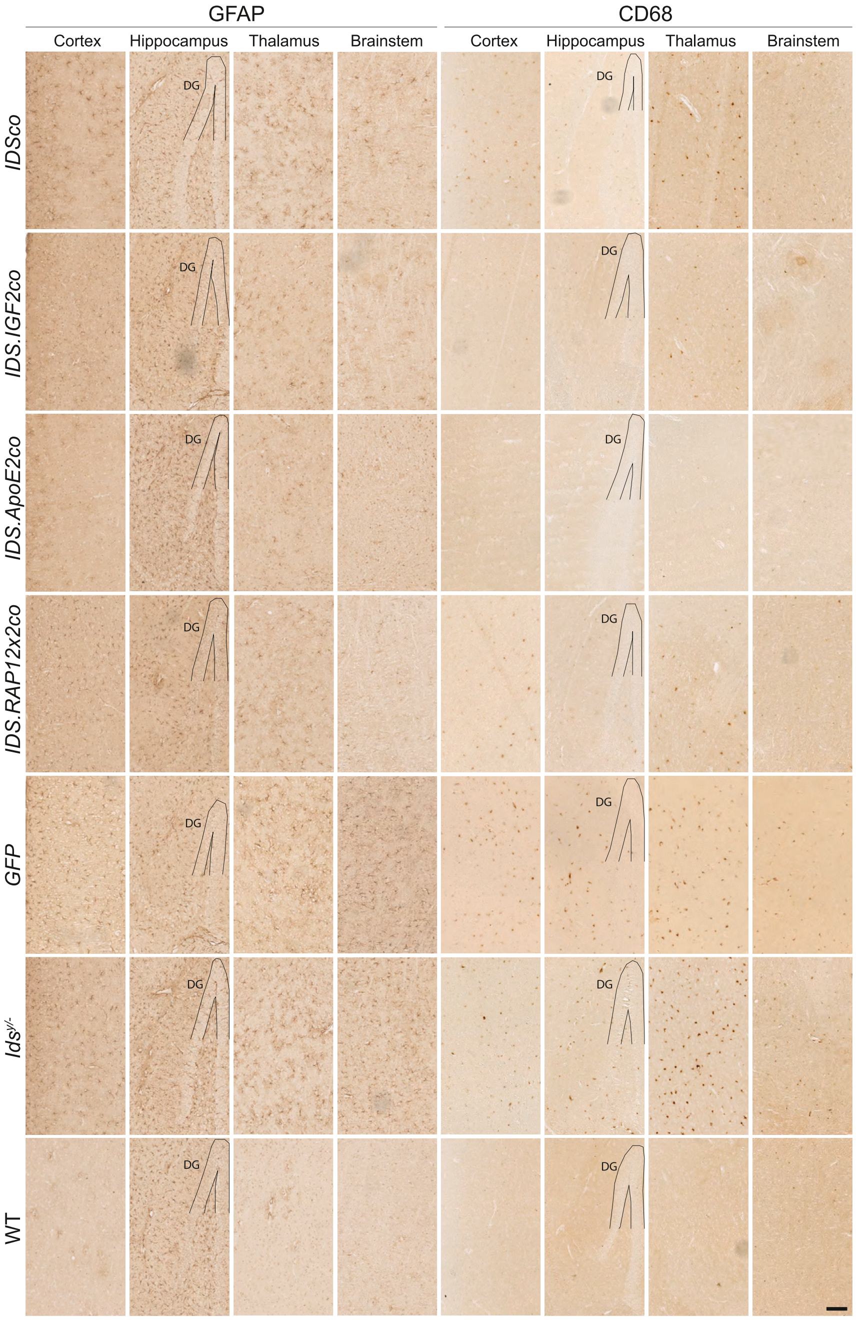

Figure 5. Gene therapy relieves neuroinflammation in brain

Representative examples of cortex, hippocampus, thalamus, and brainstem stained for GFAP and CD68 of gene therapy-treated mice and controls. DG, dentate gyrus. Scale bar, 100 μm. GFAP and CD68 stainings of corpus callosum, hypothalamus, midbrain, and cerebellum are shown in Figure S6.

immunoreactivity to similar levels (~60% reduction vs. *Ids*$^{y/-}$), with the *IDSco* vector resulting in slightly higher and the *IDS.ApoE2co* vector resulting in slightly lower immunoreactivity. GFAP immunoreactivity in the hypothalamus was marginally reduced by treatment with *IDS.RAP12x2co* and was best corrected by the *IDSco*, *IDS.IGF2co*, and *IDS.ApoE2co* vectors to levels 4, 3, and 1.6 times higher than WT, respectively. In the midbrain, treatment with *IDSco*, *IDS.RAP12x2co*, and *IDS.ApoE2co* resulted in 60% reduction of *Ids*$^{y/-}$ GFAP levels. *IDS.IGF2co* was the best performing vector in this area, causing 85% reduction of GFAP pathology to levels 2.4 times of WT. All the vectors showed comparable correction of GFAP immunoreactivity in brainstem and had no effect in the corpus callosum and hippocampus (which did not display elevated GFAP levels in *Ids*$^{y/-}$ mice), except for *IDS.ApoE2co*, which resulted in marginally increased numbers of GFAP-positive cells in the corpus callosum (Figures 5, 6C, 6D, and S5).

We also investigated clusters of differentiation 68 (CD68), a lysosomal protein expressed in the soma of microglial cells that is upregulated in functionally activated microglia.[33,34] We observed a widespread increase of CD68 immunoreactivity in brains of *Ids*$^{y/-}$ mice (Figures 5, 6E, 6F, and S5). Gene therapy with *IDSco* and *IDS.RAP12x2co* resulted in an average reduction of ~60% of CD68 immunoreactivity in all the areas analyzed. In the hippocampus, brainstem, and cerebellum, *IDSco* and *IDS.RAP12x2co* reduced the number of CD68-positive cells by ~ 75%. In the rest of the areas, *IDSco* and RAP12x2co treatments resulted in an average reduction of 50% compared with *Ids*$^{y/-}$ CD68-positive cells. *IDS.IGF2co* vector had an overall better outcome on CD68 pathology compared with both *IDSco* and *IDS.RAP12x2co*, particularly in the cortex (65% reduction vs. *IDSco* and *IDS.RAP12x2co*), hypothalamus (70% reduction vs. *IDSco* and *IDS.RAP12x2co*), and midbrain (75% reduction vs. *IDSco* and *IDS.RAP12x2co*), while *IDS.ApoE2co* further reduced CD68 levels mainly in the corpus callosum and thalamus to near WT levels.

In conclusion, we demonstrated that gene therapy in which IDS protein was fused with IGF2 and ApoE2 resulted in better correction of LAMP1, GFAP, and CD68 pathology in brain compared with untagged or RAP12x2-tagged IDS.

Low engraftment of HSC-derived cells in brain does not explain correction of brain pathology

Gene therapy caused alleviation of brain HS accumulation, lysosomal pathology, and neuroinflammation, with no (RAP12x2) to beneficial (IGF2 and ApoE2) impact of the tag used on the efficacy of the

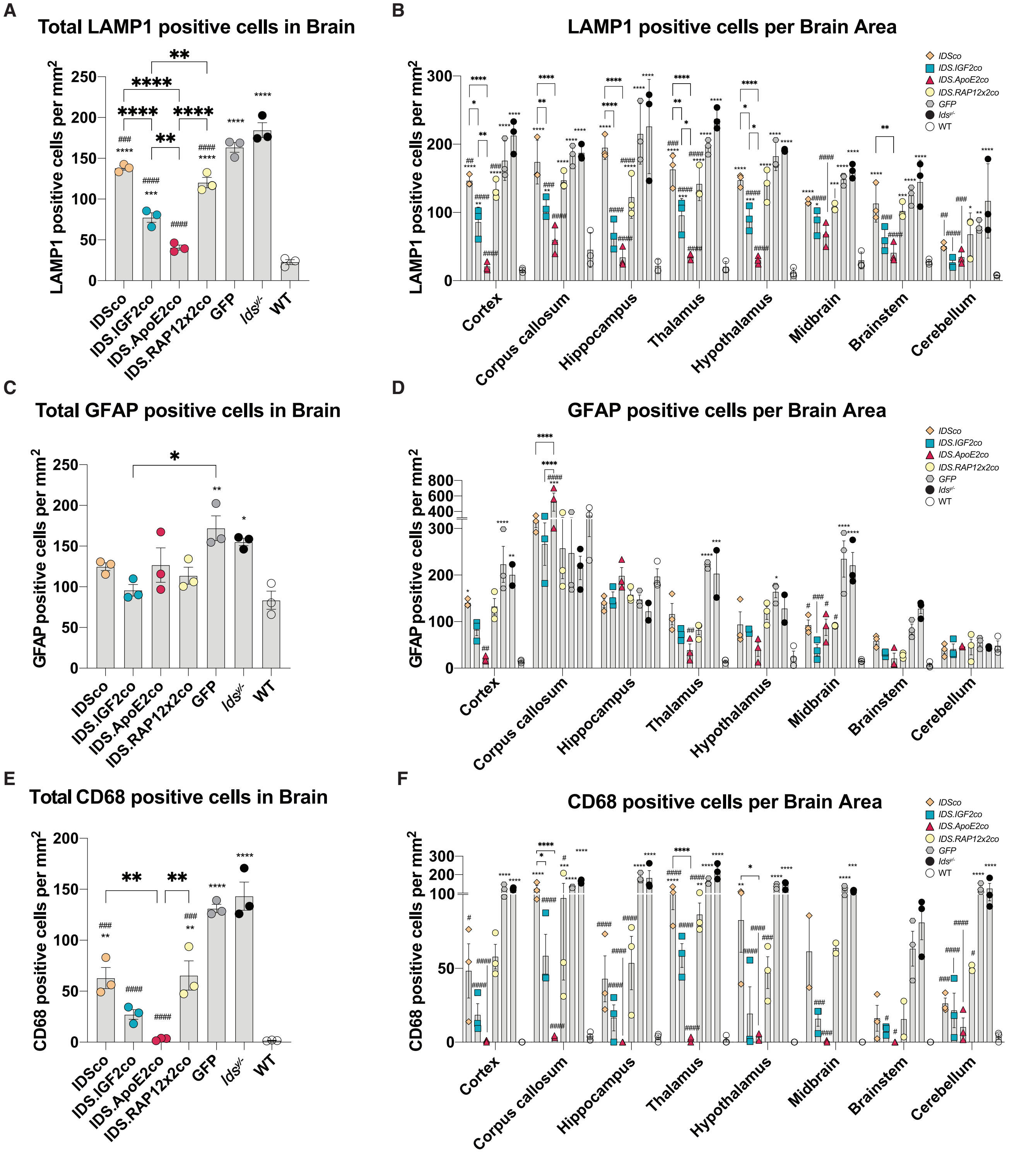

Figure 6. Quantification of LAMP1-, GFAP-, and CD68-positive cells in brain

Quantification of LAMP1 (A and B), GFAP (C and D), and CD68 (E and F) in total brain (A, C, and E) and in different brain areas (B, D, and F). Data represent means ± SEM. In (A), (C), and (E) data were analyzed by one-way ANOVA with Bonferroni's correction. In (B), (D), and (F) data were analyzed by two-way ANOVA with Bonferroni's correction, using treatment (*IDSco*, *IDS.IGF2co*, *IDS.ApoE2co*, or *IDS.RAP12x2co*) and brain areas as categorical variables. Asterisks represent significance vs. WT; hashs represent significance vs. *Ids*$^{y/-}$. Other significant comparisons are indicated by brackets. n = 3. *p ≤ 0.05, **p ≤ 0.01, ***p ≤ 0.001, ****p ≤ 0.0001. #p ≤ 0.05, ##p ≤ 0.01, ###p ≤ 0.001, ####p ≤ 0.0001.

treatment. As previous reports pointed at brain engraftment of HSPC-derived cells and *in loco* cell-mediated secretion of the therapeutic protein as the driving mechanism for brain correction after HSPC-mediated lentiviral gene therapy,[16,35–40] we performed staining for human IDS and GFP in sagittal sections of gene therapy-treated mice. We observed IDS-positive cells in the brain at levels independent of the vector used (Figures 7A–7C and S6A). IDS-positive cells were mainly detected in meninges, choroid plexus, and perivascular areas, in addition to a widespread and rare engraftment in parenchymal areas (Figures 7A–7C, S6A, and S7). No IDS staining was detected in *GFP*-treated and untreated $Ids^{y/-}$ brain sections, while immunoreactivity was observed in cortical layer II, hippocampal CA, and perivascular areas of WT mice (Figures 7, S6A, and S7). In agreement, we observed low engraftment of GFP-positive cells in brains of *GFP*-treated $Ids^{y/-}$ animals, with a similar distribution pattern compared with IDS, suggesting that IDS-positive cells are likely to be donor-derived cells (Figures 7D and S6B). Next, we assessed VCN in brain homogenates of gene therapy-treated mice. VCN in brain was between 0.005 and 0.2 vector copies per genome (average of 0.025 vector copies per genome). Similar as for VCN in bone marrow, there was a dose-dependent increase in VCN in brain after *IDS.IGF2co* treatment, which was less prominent for *IDSco* treatment (*IDS.IGF2co* MOI 3 = 0.074 VCN per genome; average of the other conditions except *GFP* = 0.02 VCN per genome; Figure 7E), while the *GFP*-treated groups showed very low brain VCN at values marginally higher than those of the untreated groups. Plotting VCN in brain vs. bone marrow or liver, an organ without physical barriers such as the BBB, revealed a weak but significant correlation between VCN in bone marrow and brain ($p = 0.0062$, $R^2 = 0.2$) and a stronger correlation between VCN in bone marrow and liver ($p < 0.0001$, $R^2 = 0.63$; Figure S8A; Table S1). Brain VCN values were ∼20-fold lower compared with liver VCN (Figure S8A; Table S1), suggesting a much lower cerebral engraftment of donor-derived cells in brain than in liver. In agreement, analysis of chimerism in brain showed low levels of around 1 donor cell every 100 resident cells, with similar levels in the different treatment groups (Figure 7F). Of note, marginally lower chimerism values were detected in experiment 2 compared with experiment 1 (experiment 1 = average 1.42% brain chimerism; experiment 2 = average 0.79% chimerism; Figure 7F). We observed no correlation between brain HS levels and VCN or chimerism in brain for all the vectors (Figures 7G and 7H). This suggests that the improved efficacy observed after *IDS.IGF2co* and *IDS.ApoE2co* treatments is not caused by an enhanced brain engraftment of donor-derived cells.

Improved correction of brain pathology could be explained by the levels of affinity of the fusion proteins for their target receptors. For this reason, we tested the binding affinity of IDS, IDS.IGF2, IDS.ApoE2, and IDS.RAP12x2 proteins for the LRP-1 receptor (ApoE2 and RAP12x2) and domain 11 of the IGF2R (IGF2) by performing receptor ELISAs. LRP-1 receptor is known to mediate transcytosis across the BBB and its cluster IV is targeted by ApoE2 and RAP12 tags. Domain 11 of the CI-M6P/IGF2R binds with high affinity to IGF2 peptide, but does not bind M6P moieties that are targeted by repeats 3 and 9 of the same receptor. As expected, IDS protein did not bind to LRP-1 cluster IV, while IDS.ApoE2 efficiently bound this receptor resulting in a K_d of 319.2 ng/mL (Figure 7I). Binding of IDS.RAP12x2 to LRP-1 cluster IV was very weak and resulted in a K_d value that was ∼75-fold higher than the K_d of IDS.ApoE2 (Figure 7I; Table S1). This may indicate that the RAP12x2 epitope when fused to IDS is not available for binding to the LRP-1 receptor. In addition, IDS tagged with RAP22 (IDS.RAP22), a tag comprising a longer sequence of the LRP-1 binding domain of the RAP protein and with higher affinity for the LRP-1 (RAP22: aa: 251–272 of the RAP protein; RAP12: aa: 251–262 of the RAP protein),[26] did not efficiently bind to LRP-1 cluster IV (Figure S8B). This suggests that the RAP22/12 sequences are incompatible as epitope tag for IDS under the conditions employed. Functional ELISA for the IGF2R domain 11 showed that IDS did not efficiently bind, as expected, while binding of IDS.IGF2 had high affinity, resulting in a K_d of 225.7 ng/mL (Figure 7J; Table S1). IDS.IGF2 did not bind to LRP-1 cluster IV (data not shown).

We analyzed the correlation between IDS plasma activity levels and correction of brain HS (Figure 7K). Non-linear regression analysis showed correlation between brain HS and IDS activity levels in plasma, resulting in an exponential decay curve for all vectors (Figure 7K; Table S1). *IDSco* and *IDS.RAP12x2co* treatments showed similar plateaus and λ values of around 1.07 and 1.15 μg/mg (Table S1). *IDS.IGF2co* and *IDS.ApoE2co* treatments plateaued at lower values that were similar (*IDS.IGF2co*: 0.35 μg/mg; *IDS.ApoE2co*: 0.20 μg/mg) and also showed similar λ values (*IDS.IGF2co*: 0.09 $[\text{nmol/mg} \times 4\text{ h}]^{-1}$; *IDS.ApoE2co*: 0.05 $[\text{nmol/mg} \times 4\text{ h})^{-1}$) (Table S1). IDS activity in plasma correlated more with HS levels compared with VCN and chimerism in brain for all the vectors (compare Figure 7K with Figures 7G and 7H).

DISCUSSION

As we learnt about the limitations of ERT in the treatment of the neurological manifestations of MPS II, HSPC-LVGT emerged as a candidate approach for addressing this challenge. Using a highly sensitive GAG detection method (HPLC-MS/MS), which revealed a broader detection range compared with previous studies (7.5-fold,[23] 1.2-fold[20,22]), we demonstrated that fusion of IDS with IGF2, as well as the previously published IDS.ApoE2[23] in combination with the MND promoter, provided a near complete prevention of neurological pathology in $Ids^{y/-}$ mice during HSPC-LVGT. These results identify the MND-driven *IDS.IGF2co* and *IDS.ApoE2co* as candidate transgenes for future clinical developments of HSPC-LVGT for Hunter disease, and provide insight into their mechanisms of action.

Untagged IDS was only moderately effective after HSPC-LVGT, reducing brain HS levels and slightly ameliorating pathology, as shown previously.[20–22] As we found engraftment of small numbers of donor-derived cells in the same cerebral areas where most of the Alcian blue staining—and thus sulfated GAG—was detected (e.g., in choroid plexus, meninges and some perivascular areas), we hypothesize that brain-engrafted donor-derived cells were responsible for the correction of HS levels in these areas via cross-correction

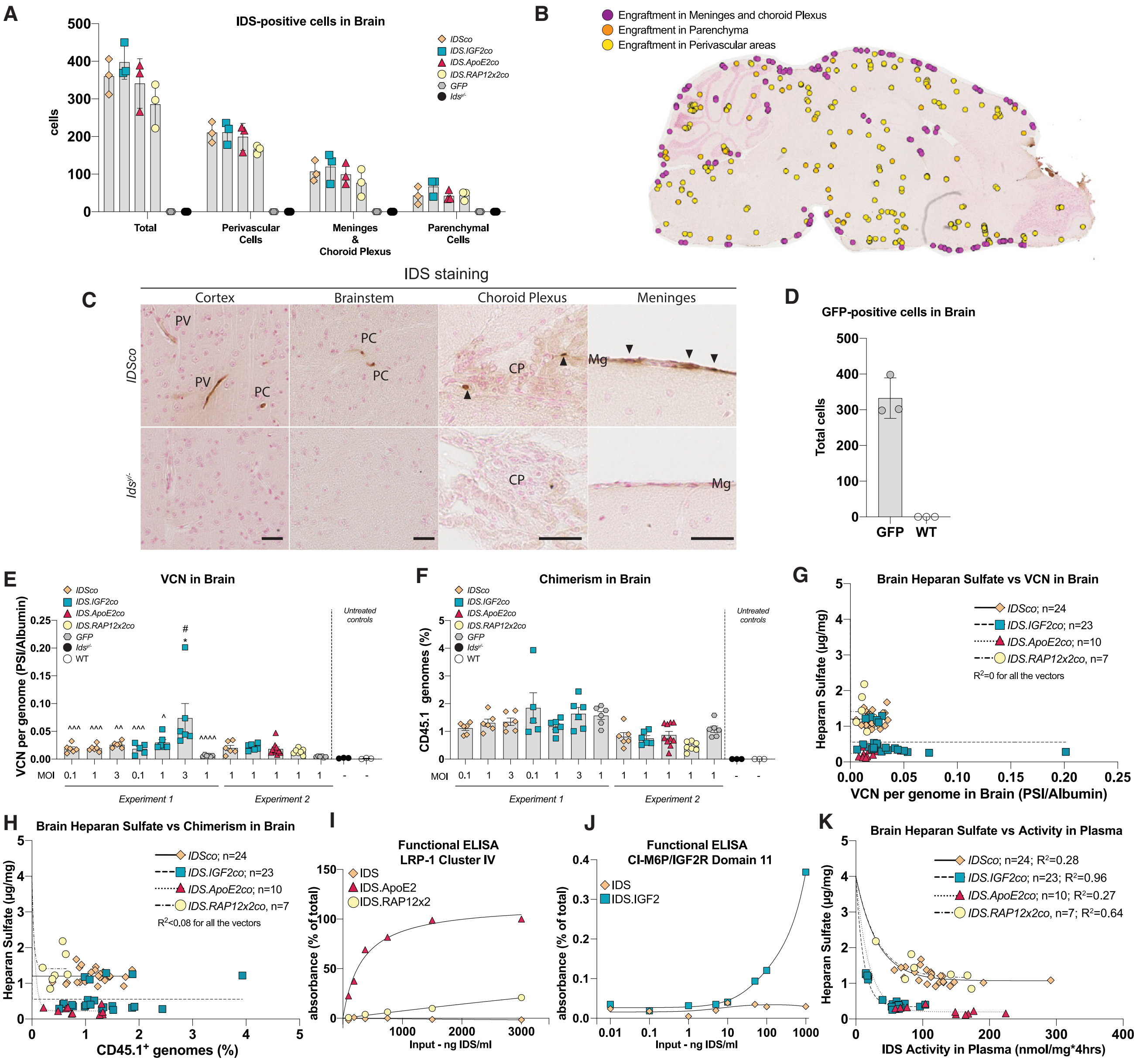

Figure 7. Mechanisms of brain correction after gene therapy

(A) Quantification of IDS-positive cells in brains of gene therapy-treated mice and untreated *Ids*$^{y/-}$ and WT mice. (B) Example of distribution of IDS-positive cells in a *IDSco*-treated mouse. Examples of other treatment conditions are shown in Figure S7. (C) IDS staining in sagittal sections of cortex, brainstem, choroid plexus, and meninges. Representative pictures of other treatment conditions are shown in Figure S6. Scale bar, 25 μm. (D) Quantification of GFP-positive cells in brain of *GFP*-treated *Ids*$^{y/-}$ mice and WT. Representative pictures are shown in Figure S6. (E) VCN per genome measured in brain by qPCR on *PSI* and *Albumin* loci. (F) Chimerism analysis of brain measured by allele-specific qPCR on the *Cd45.1* locus. Validation data are shown in Figure S10. (G) Non-linear regression analysis between VCN in brain and cerebral heparan sulfate. (H) Non-linear regression analysis between chimerism in brain and heparan sulfate in brain. (I) Functional ELISA analysis of IDS, IDS.ApoE2, and IDS.RAP12x2 proteins using the LRP-1 receptor (cluster IV). (J) IDS and IDS.IGF2 proteins binding to the CI-M6P/IGF2 receptor (domain 11) in a functional ELISA. (K) Non-linear regression analysis between brain heparan sulfate content and IDS enzyme activity in plasma. Data are presented as means ± SEM. In (E) and (F) data were analyzed by one-way ANOVA with Bonferroni's correction. Regression analysis of (G), (H), and (K) is shown in Table S1. Asterisks represent significance vs. WT; hashs represent significance vs. *Ids*$^{y/-}$. In (E) ^ represent significance vs. the *IDS.IGF2co* MOI 3 group. Other significant comparisons are indicated by brackets. PV, perivascular cells; PC, parenchymal cell; Mg, meninges; CP, choroid plexus. (A–C) n = 3. (E and F) Experiment 1: n = 5 or 6 per group. Experiment 2: *IDSco*, *IDS.IGF2co*, *GFP*, *Ids*$^{y/-}$ and WT n = 6; *IDS.ApoE2co* n = 10; *IDS.-RAP12x2co* n = 7. (G and H) n = 6. (I and J) n = 3. *p ≤ 0.05. #p ≤ 0.05. ^p ≤ 0.05, ^^p ≤ 0.01, ^^^p ≤ 0.001, ^^^^p ≤ 0.0001.

that was not largely dependent on the protein tag. Alcian blue staining was, however, still detected in some perivascular areas and rare parenchymal cells after *IDSco* gene therapy, along with a widespread increase of GFAP-positive and CD68-positive cells, suggesting that untagged IDS, when produced locally by engrafted donor-derived cells, is unable to reach all brain cells that are affected in *Ids*$^{y/-}$ mice. A further correction of brain pathology was only achieved upon IGF2 and ApoE2 tagging of IDS, but not RAP12x2 tagging. The observed enhanced correction mediated by the IGF2 and ApoE2 tags was independent of the extent of HSPC-derived cells engraftment in brain, which represents a well-established correction mechanism for the brain during HSPC-LVGT for LSD.[19,40] Below we elaborate upon these findings.

IGF2-mediated correction of brain pathology

The improved correction of brain pathology observed after *IDS.IGF2co* gene therapy is in line with our previous report showing improved correction of the CNS after gene therapy with IGF2-tagged *GAA* in murine Pompe disease,[25] and provides evidence that IGF2-tagging might be a general strategy to increase the therapeutic efficacy of HSPC-LVGT for brain manifestations of LSDs. The rationale of IGF2 tagging of lysosomal proteins is based on the ability of IGF2 to bind the CI-M6P/IGF2R with an affinity higher than the affinity of mannose 6-phosphate for the same receptor.[25,41] In agreement, IDS.IGF2 protein showed high affinity binding to the CI-M6P/IGF2R during a functional ELISA assay, as well as 5-fold higher cellular uptake levels over IDS (Figures 1G, 1H, 7I, and 7J). These results, together with previous reports showing enhanced uptake into astrocytes and neurons upon IGF2 tagging of lysosomal proteins,[42,43] point at enhanced cross-correction of affected cells as a prominent candidate mechanism driving the IGF2-mediated correction of brain pathology.

IGF2 protein was also shown to accumulate in the brain parenchyma upon intravenous infusion,[44–47] suggesting an active transport of IGF2 from the blood to the brain, although the mechanisms responsible remain to be elucidated. The CI-M6P/IGF2R might be involved in a blood-to-brain transport of IGF2 as this receptor was shown to mediate transcytosis across the BBB, but only in the perinatal period and up to age 7 weeks in mice.[48–50] In addition, IGF2 can also efficiently bind IR-A and IGF1R—both shown to mediate transcytosis across the BBB of IGF1 and insulin—with an affinity of 1 order of magnitude within the affinity of insulin and IGF1 for these receptors, respectively.[46,51] Future work is required to determine to what extent and how IDS-IGF2 may undergo transcytosis during HSPC-LVGT in *Ids*$^{y/-}$ mice.

Tagged IDS protein might also reach the brain via CSF-to-brain transport, a mechanism that could take place following engraftment of donor-derived cells in meninges or choroid plexus—as documented in Figure 7—and secretion into the CSF, or upon transport of tagged IDS from blood to the CSF at the choroid plexus. Such a transport has been suggested for IGF1,[46,52] and, given the high structural and sequence similarity between IGF1 and IGF2,[53–55] it is possible that a similar transport mechanism plays a role in the IGF2-mediated correction of pathology in brain. In line with this hypothesis, ICV injection of IGF2-tagged NAGLU has been reported to normalize brain pathology in an MPS IIIB mouse model,[56] although therapeutic efficacy upon ICV injection has also been reported for untagged IDS.[57]

ApoE2-mediated correction of brain pathology

The generation of ApoE2-tagged IDS was described previously as a strategy to combine a brain-shuttle peptide with gene therapy for the correction of brain pathology in an MPS II mouse model.[23] The design for tagged IDS described by these authors was left unchanged in the experiment reported here and only the promoter driving the transgene expression was changed (MND instead of CD11b). IDS.ApoE2 protein has been shown to undergo transcytosis *in vitro* in a transwell experiment with bEND.3 cells.[23] In agreement, we showed that IDS.ApoE2, but not IDS.RAP12x2 or untagged IDS, efficiently bound LRP-1, a receptor capable of transcytosis across the BBB.[58,59] This is consistent with the enhanced efficacy in brain of IDS.ApoE2 and the lack of an effect on brain pathology of RAP12x2 tagging of IDS, and strongly implies an active blood-to-brain transport, particularly transcytosis at the BBB, for the IDS.ApoE2 fusion protein. However, we cannot conclusively rule out other potential ApoE2-mediated correction mechanisms such as increased uptake into affected cells via cross-correction, which is in line with our findings in MPS II fibroblasts and bEND.3 cells and with previous studies documenting an augmented uptake into neurons and glia of lysosomal proteins after ApoE2 tagging.[23,60–62]

Comparison of IGF2- and ApoE2-mediated correction of brain pathology

Additional clues for the mechanism underlining IGF2- and ApoE2-mediated correction of brain pathology come from the comparison of the therapeutic efficacy of *IDS.IGF2co* and *IDS.ApoE2co* gene therapy. We found that residual brain HS levels after *IDS.IGF2co* (plateau 0.46 μg/mg HS) and *IDS.ApoE2co* (plateau 0.21 μg/mg HS) gene therapy correlated with IDS enzyme activity in plasma, with activity levels being lower in plasma for *IDS.IGF2co*-treated mice compared with the *IDS.ApoE2co* treatment group. Although the causality of this relationship needs to be further investigated, it is consistent with the presence of transport from blood to brain, which is expected to be limited by the plasma availability of tagged IDS. In addition, plasma concentration appears to be predictive for the correction of brain pathology after gene therapy with *IDS.ApoE2co* or *IDS.IGF2co*, as it correlated more with the HS levels after *IDS.IGF2co* and *IDS.ApoE2co* treatments compared with VCN and brain activity (compare Figure 7K vs. Figures 3C and 3D). This observation is relevant for a future clinical implementation of HSPC-LVGT, as measurement of plasma activity is minimally invasive for patients and broadly available as a diagnostic tool. However, the presence of a blood-to-brain transport mechanism for IDS.IGF2 and IDS.ApoE2—and thus the delivery of additional IDS protein copies to the brain from the bloodstream—is in disagreement with the observed comparable levels of IDS activity in brain homogenates after treatment with tagged or untagged IDS. Such a discrepancy was previously shown after HSCP-LVGT experiments in Pompe[25] and Hunter[23] murine models, where tagged and

untagged enzymes were compared. We hypothesize that most of the activity detected in total brain homogenates originate mainly from brain-engrafted donor-derived cells which were present at comparable levels in the different treatment conditions, as shown by immunohistological staining of the IDS protein, as well as by VCN and chimerism analysis of brain homogenates. On the other hand, tag-mediated enhanced correction of brain pathology is likely to arise from small differences of IDS protein levels, which could not be distinguished by the IDS enzyme activity assay and could not be detected by the immunohistological protocol employed. For example, intravenous injection of IDS fused to a Tfr-binding transport vehicle was shown to result in only ~0.3% transport to the brain 2 h after injection,[8] while BBB transport models suggested that the transport is limited by exocytosis to the abluminal side, estimated to have a $t_{1/2}$ of ~14 h,[63] which is comparable with the cellular half-life of the IDS protein (~1.5 days in MPS II fibroblasts, data not shown). Future work is required to determine the possible contribution of transport from blood to brain to the correction or brain pathology in $Ids^{y/-}$ mice after HSPC-LVGT with *IDS.IGF2co* and *IDS.ApoE2co*.

In conclusion, the enhanced correction of brain pathology during HSPC-LVGT with *IDS.IGF2co* and *IDS.ApoE2co* gene therapy is likely to arise from multiple mechanisms—which may include engraftment of HSPC-derived cells into the brain, increased uptake into affected cells, and active transport from blood to brain—that are not necessarily the same for these two tags and that might act concomitantly for the correction of brain pathology.

Concluding remarks

In the experiments here reported, we used the MND promoter to drive transgene expression. This allowed supraphysiological expression for all the vectors tested, resulting in levels ~30- to 150-fold higher than WT mice at VCN lower than 2. This result expands upon previous reports of HSPC-LVGT in $Ids^{y/-}$ mice with MND or MND-derived promoters.[20–22] The MND promoter was shown to be safe in a number of clinical trials and pre-clinical works, without evidence of genotoxicity in long-term reports.[17,24] Despite this, ongoing trials for cerebral adrenoleukodystrophy (CALD) (NCT01896102; NCT03852498) reported 3 patients out of 67 with myelodysplastic syndrome. These patients were transplanted with HSPCs treated with Lenti-D, a lentiviral vector encoding *ABCS1* under the control of the MND promoter, which was suspected to cause the transactivation of neighboring genes.[64,65] In this regard, the Food and Drug Administration (FDA)'s Cellular, Tissue, and Gene Therapies Advisory Committee (CTGTAC) decided that the benefits of the therapy outweigh the risks for any sub-population of children with CALD.[64–67] For this reason, it remains important to evaluate the genotoxic potential of the lentiviral vectors here presented before and during clinical implementation.

During HSPC-LVGT, the preconditioning regimen applied is one of the main factors influencing the engraftment of HSPC-derived cells in the brain.[18,19,38,40,68–71] Here, we used a myeloablative dose of total body irradiation (TBI) as preconditioning prior to HSPC-LVGT, which resulted in low brain engraftment of ~1%, as previously reported.[20–22] Preconditioning with a myeloablative dosage of busulfan was previously tested during HSPC-LVGT in $Ids^{y/-}$ mice and resulted in VCN in bone marrow and brain[23] at levels comparable with the ones reported by us and by others[21] using TBI in $Ids^{y/-}$ mice, suggesting similar cerebral engraftment of donor cells in the two procedures. In addition, the distribution of donor-derived cells documented by us is comparable with the distribution observed in an MPS II patient receiving cord-blood transplantation upon busulfan, cyclophosphamide, and fludarabine conditioning.[72] In this case report, the authors showed that brain engraftment of donor-derived cells was low—at levels lower than liver, as shown by us—and was insufficient for improvement of brain pathology. In line with this, we showed that HSPC-LVGT resulted in low engraftment of donor cells and, using untagged IDS, the efficacy of the treatment plateaued at partial correction of pathology which could not be increased by increasing the dose of cell engraftment to the brain or the MOI (this study; similar results were obtained for Pompe disease).[25] The incorporation of protein tagging in the design of HSPC-LVGT enabled a more potent correction of brain pathology and could therefore be the key to a more efficacious clinical application of HSPC-LVGT for the treatment of severe neurological conditions like MPS II.

MATERIALS AND METHODS

Animals and procedures

Heterozygous female B6N.Cg-Ids^{tm1Muen}/J mice were purchased from the Jackson Laboratory (JAX stock no. 024744)[73] and bred with WT C57BL/6J males (JAX stock no. 024744) to generate male hemizygous $Ids^{y/-}$ B6N.Cg-Ids^{tm1Muen}/J mice (hereafter $Ids^{y/-}$) and male wild-type B6N.Cg-Ids^{tm1Muen}/J littermates (hereafter WT). Heterozygous female B6N.Cg-Ids^{tm1Muen}/J mice were backcrossed with B6.SJL-*Ptprc*^{a} *Pepc*^{b}/BoyJ (JAX stock no. 002014) and selected for *Ptprc*^{a} (via quantitative polymerase chain reaction [qPCR], see below) and $Ids^{+/-}$ to generate heterozygous female $Ids^{+/-}$ on the B6.SJL-*Ptprc*^{a} *Pepc*^{b}/BoyJ congenic background (hereafter $Ids^{+/-}$ CD45.1). Heterozygous female $Ids^{+/-}$ CD45.1 were bred with wild-type male B6.SJL-*Ptprc*^{a} *Pepc*^{b}/BoyJ to generate male hemizygous $Ids^{y/-}$ B6N.Cg-Ids^{tm1Muen}/J mice (hereafter $Ids^{y/-}$ CD45.1), which were used as donor mice to distinguish donor and recipient cells via the *Ptprc*^{a} pan leukocytes marker, as previously described.[23] Mice were bred according to standard procedures of the Laboratory Animal Science Center (EDC) at the Erasmus MC, which included a 12-h light-dark cycle and *ad libitum* diet. Genotyping for the *Ids-KO* locus was performed as described by Jackson Laboratory. Genotyping of the CD45.1/2 locus was performed via qPCR as described below. All mice used in this study were grouped in two to four animals at weaning and housed under specific pathogen free conditions at the EDC. At the end of the experiment, mice were anesthetized by ketamine (10%, Alfasan, Woerden, the Netherlands) and medetomidine (1 mg/mL, Eurovet, Bladel, the Netherlands) and sacrificed by intracardiac perfusion with 50 mL of phosphate-buffered saline (PBS). Relevant tissues were harvested and processed according to the follow-up analysis. All animal experiments in this study were approved by the Animal Experiments Committee (DEC) in the Netherlands and these complied with the Dutch legislature to use animals for scientific procedures.

Lentiviral vector construction, production, and titration

Codon-optimized human *IDS* and *IDS.ApoE2* (kindly provided by Prof. Dr. Brian Bigger) were cloned into the third-generation self-inactivating lentiviral pCCL vector pCCL-MND-c.o.RAG1[74] (kindly provided by Prof. Dr. Frank Staal) by replacing the *RAG* gene using BamHI and SalI restriction sites to generate pCCL-MND-*IDSco* (hereafter *IDSco*) and pCCL-MND-*IDS.ApoE2co* (hereafter *IDS.ApoE2co*). *IDSco* encodes IDS aa: 1–550, while *IDS.ApoE2co* encodes IDS aa: 1–550 fused C-terminally to ApoE2 (LRK LRK RLL LRK LRK RLL)[75] via the Leu(Gly-Gly-Gly-Gly-Ser) × 4 linker sequence. Codon-optimized cassettes encoding IDS aa: 530–550 fused to either IGF2 (ALC GGE LVD TLQ FVC GDR GFY FSR PAS RVS RRS RGI VEE CCF RSC DLA LLE TYC ATP AKS E)[25,41] or to a minimal peptide of the human receptor-associated protein (RAP12x2; AKI EKH NHY QK G AKI EKH NHY QK)[41] were subcloned into the *IDSco* vector to generate pCCL-MND-*IDS.IGF2co* (hereafter *IDS.IGF2co*) and pCCL-MND-*IDS.RAP12x2co* (hereafter *IDS.RAP12x2co*). In some experiments (Figure S8B), pCCL-MND-IDS.RAP22co (hereafter IDS.RAP22co) was used. IDS.RAP22co was cloned as described for *IDS.IGF2co* and *IDS.-RAP12x2co* by subcloning into *IDSco* a cassette comprising IDS aa: 530–550 fused to RAP22 (EAK IEK HNH YQK QLE IAH EK).[41] *GFP* sequence from the pRRL.PPT.SF.GFP.bPRE4*.SIN[25] vector was cloned into the pCCL-MND-c.o.RAG1[74] using BamHI and SalI restriction sites. N-terminal tagging of IDS with IGF2 was attempted by fusing IGF2 to IDS aa: 26–550 via the Gly-Ala-Pro linker sequence. The resulting fusion protein was not functional as the precursor protein failed to be processed to the mature form and showed no intracellular or secreted enzymatic activity (data not shown). Lentiviral particles were generated by calcium-phosphate transfection of HEK293T cells. In brief, HEK293T cells were plated in complete medium (Dulbecco's modified Eagle's medium [DMEM]) (10% FCS, 1% penicillin/streptomycin [PS]; see transfection and uptake of IDS for further details on the medium) at a confluence of 120,000 cells/cm^2 in a T175 flask (Sarstedt) 24 h before transfection. The following day, complete medium was replaced with 20 mL of PS-free medium. The transfection solution was then prepared using the following procedures: 24 μg of transfer vector were mixed with 15.6 μg of pMDL-g/pRRE, 8.4 μg pMD2-VSVg, 6 μg of pRSV-Rev, 1080 μL of double-distilled water, and 120 μL of 2.5 M calcium chloride (Sigma).[76] After 5 min incubation, 1,200 μL of HEPES buffer (pH 7.1) (Sigma) were added dropwise while bubbling the solution. The resulting solution represents the transfection solution that was immediately added to the T175 flak. Twenty-four hours after transfection, the medium was replaced with 20 mL of complete medium. The day after, lentiviral particles were concentrated by ultracentrifugation (Beckman, SW32Ti rotor) of the medium supernatant at 20,000 rpm for 2 h at 4°C. Concentrated viral particles were resuspended in 100 μL of PBS. This was achieved by pipetting 40 times, with intervals of 20 min, over a span of 1 h, while on ice. Aliquots (10 μL) were stored at −80°C until use. Functional viral titers were measured by transduction of HeLa cells as shown previously.[25] Specifically, 200,000 HeLa cells were seeded in each well of a 6-well plate about 8 h prior to transduction. To the viral stock (10 μL), we added 990 μL of complete medium (1 mL in total). Thereafter, 500, 50, or 5 μL of the diluted virus was added to the HeLa cells incubated in 2,500, 2,950, or 2,995 μL of complete medium, respectively. This ensured that each viral titer was assessed in three-log dilutions. The subsequent day, the medium was refreshed, as well as on day 3 after transduction, and cells were harvested on day 5 after transduction. Genomic DNA was purified as described below. VCN per genome was determined as described below. Viral titers were calculated using the formula: {VCN × 200,000(number of HeLa cells plated) × dilution factor}/mL, where the dilution factors are 1:2 for 500 μL of virus, 1:20 for 50 μL, and 1:200 for 5 μL. In transduction experiments, an MOI of 1 is defined as the amount of virus capable of transducing a given number of cells (in this case, HeLa cells), resulting in VCN per genome of 1 (e.g., if the viral titer of a given viral batch is 10^9/mL, 1 μL of this batch is the amount of virus needed to infect 10^6 HeLa cells at MOI of 1, resulting in a VCN per genome of 1).

Transfection and uptake of IDS versions

For analysis of secretion, HEK293T cells were transfected as shown previously.[25,77] In short, *IDSco*, *IDS.IGF2co*, *IDS.ApoE2co*, *IDS.-RAP12x2co*, or *GFP* lentiviral transfer plasmids were transfected into HEK293T cells via calcium phosphate transfection in complete medium (1% PS, Gibco 15070) and 10% fetal bovine serum (FBS-12A) (Capricorn Scientific) in high-glucose DMEM (Gibco). Twenty-four hours after transfection, the medium was replaced. In transfection + competition experiments (Figure S1E), the medium was replaced with complete medium containing 1.5 μM of recombinant IGF2 (Cell Sciences MU100). In secretion experiments (Figures 1D and 1F) 200 μL of medium was sampled every 24 h starting from 48 h and up until 96 h post-transfection for IDS enzyme activity analysis. At 96 h, cells were washed with PBS and lysed as described below. IDS activity was measured in media samples and cell lysate using 4-methylumbelliferyl-α-L-idopyranosiduronic acid 2-sulphate disodium salt analysis as described below. Western blot analysis was performed on cell lysate and media samples at 96 h post-transfection as described below. Transfection efficiency was measured by qRT-PCR analysis of the expression of *PSI* on the lentiviral vector (FW primer: 5′-CAG GAC TCG GCT TGC TGA AG; RV primer: 5′-TCC CCC GCT TAA TAC TGA CG) and normalized by GAPDH expression (FW primer: 5′-CGG TTT CTA TAA ATT GAG CCC G; RV primer: 5′-GCG ACG CAA AAG ATG C) using qRT-PCR, as described previously.[77] For measuring of the relative specific activity, biological triplicates were combined and IDS activity levels were measured in media and cell lysates. RNA was extracted using the RNeasy Micro Kit (QIAGEN, 74004) and cDNA was obtained using the iScript cDNA Synthesis Kit (Bio-Rad, 1708891). Activity levels were equalized by dilution in water (cell lysates) or complete medium (media) and three 2-fold dilutions were performed (1, 1:2, and 1:4). IDS enzyme activity and western blot analysis was performed in each dilution as described below. For uptake experiments, HEK293T cells were transfected as described above. Twenty-four hours after transfection, transfection medium was replaced with complete medium which was harvested after 24 h and used as conditioned medium. Conditioned medium containing secreted IDS proteins or from *GFP*-mock transfection was centrifuged at 300 × *g* for 5 min and filtered (0.45 μm filter, Millipore). Media were aliquoted and stored at −80°C. Protein concentration was measured by IDS

sandwich ELISA in one of the aliquots. Uptake experiments were performed on primary MPS II fibroblasts or on bEND.3 cells (seeding density 48 h before start of uptake: bEND.3 cells 75,000 cells/cm^2, MPS II fibroblasts 50,000 cells/cm^2) via incubation in conditioned medium (frozen aliquots of conditioned medium were thawed and warmed up to 37°C before incubation) at the indicated IDS protein concentrations for 18–24 h. Captured IDS protein was measured by IDS sandwich ELISA as described below.

Western blotting

Protein extracts from HEK293T cells and medium supernatant, bone marrow, and plasma were obtained as described below (IDS activity section). Protein concentration was determined using a Pierce BCA Protein Assay Kit (Thermo Fisher Scientific) according to the manufacturer's instructions. For SDS-PAGE analysis of IDS protein, a total of 20 μg (transfection, cells), 12 μL (transfection, medium) or 267 μg (plasma) of total protein were used. Sample preparation and SDS-PAGE analysis were performed as described previously.[78] In brief, samples were denatured by dilution with 5× Laemmli sample buffer (62.5 mM Tris-HCL [pH 6.8], 2% SDS, 25% glycerol, 0.01% bromophenol blue, 5% β-mercaptoethanol) and heating at 95°C for 5 min. SDS-PAGE was performed on a 4%–15% polyacrylamide gel (Criterion TGX, Bio-Rad) and total protein load was measured using Gel-doc XR+ (Bio-Rad). Proteins were transferred to nitrocellulose blotting membranes (GE Healthcare) and blocked with 5% non-fat milk powder in PBS and probed by overnight incubation at 4°C with goat anti-human IDS (1:1,000, R&D Systems) in 5% non-fat milk powder in PBS supplemented with 0.1% Tween. Proteins were detected with IRDye 800 CW or IRDye 680 RD secondary antibodies (1:10,000; LI-COR Biosciences, Lincoln, NE) and were imaged using the Odyssey Infrared Imaging System (LI-COR Biosciences). Protein content was quantified using Fiji; equal loading was determined by quantification of the total bands using the stain-free signal on the same gel used for immunoblotting. Quantification was performed within the linear range of detection (S1C).

IDS enzyme activity

Brain samples (right hemisphere) were disrupted with metal beads (stainless steel beads 5 mm, QIAGEN) in 1 mL of double-distilled water by TissueLyser II (QIAGEN, Venlo, the Netherlands) at 30 Hz for 10 min. Disrupted brain samples (200 μL) were diluted with 200 μL of 0.4% Triton X-100 (Sigma) and further disrupted with a Disruptor Genie (Scientific Industries) for 2 min. Debris was pelleted at 12,000 × *g* for 10 min at 4°C. Supernatant was diluted 4 times in double-distilled water for measurement of IDS enzyme activity and 12 times for measurement of total protein. Extracts from transfected HEK293T cells and bone marrow were obtained in 100 μL of water by snap-freezing on dry-ice and mechanical disruption. Debris was pelleted by centrifugation at 10,000 rpm for 5 min. Medium from HEK293T cell transfection was centrifuged at 10,000 rpm for 5 min to remove debris. To obtain plasma, blood samples were mixed 3:1 with 4% citrate buffer (Sigma, 6132-04-3) and plasma was separated by centrifugation at 2,000 × *g* for 10 min at 4°C. Lysate from HEK293T cells was diluted 17 times in 0.2% bovine serum albumin (BSA) in water (BSA, Sigma) for measurement of IDS enzyme activity, and 9 times in water for measurement total protein. Medium from transfected 293T cells was diluted 5 times in complete medium for measurement of IDS activity. Lysate from bone marrow was diluted 51 times in 0.2% BSA in water for measurement of IDS enzyme activity and 6 times in water for measurement of total protein. Plasma was diluted 21 times in 0.2% BSA in water for measurement of IDS enzyme activity and 31 times in water for measurement of total protein. IDS activity was measured by incubation of 5 μL samples with 5 μL of 4-methylumbelliferyl-α-L-idopyranosiduronic acid 2-sulphate disodium salt (Biosinth, Carbosynth; 2.5 mM in 0.2 M Na-acetate buffer [pH 5]) and 5 μL of recombinant human α-L-iduronidase (5 μg/mL in 0.1% BSA; R&D Systems) for 4 h at 37°C.[79] During each IDS activity measurement, dilutions of Elaprase at 1, 0.1, 0.01, and 0.003 ng/μL were measured as a control for the linear range of detection of the assay.

Lentiviral hematopoietic stem cell transduction and transplantation procedures

HSPC-LVGT was conducted in two experiments as described in the results section. Each set of experiment had *GFP*, $Ids^{y/-}$ and WT controls. The experiments were performed by the same investigators (F.C. and E.V.) using identical procedures. Bone marrow cells were harvested from 8-week- to 4-month-old male $Ids^{y/-}$ CD45.1 mice. Hematopoietic stem and progenitor cells were enriched by lineage depletion using the Lineage Cell Depletion Kit - mouse (Miltenyi Biotec). Lin^- cells were seeded at a density of 10^6 cells/mL in StemSpan SF expansion medium (STEMCELL Technologies) supplemented with recombinant murine thrombopoietin (10 ng/mL, R&D Systems), recombinant murine stem cell factor (100 ng/mL, R&D Systems), and recombinant murine FMS-like tyrosine kinase 3 murine ligand (50 ng/mL, R&D Systems). After 24 h of expansion, cells were transduced without transduction enhancers over 24 h at the indicated MOI with concentrated lentiviral particles and incubated at 37°C with 5% CO_2. The day after, 1×10^6 transduced Lin^- cells (200 μL of cells suspension in PBS) were transplanted intravenously into 8-/11-week-old male $Ids^{y/-}$ CD45.2 recipients, previously subjected to 9 Gy TBI using the Gammacell 40 irradiator (Atomic Energy of Canada, Ontario, Canada). No normalization for body weight was applied to the number of cells transplanted.

Mass spectrometry analysis of HS and DS in brain

Brain samples were prepared as described in the enzyme activity assay section (samples were not further diluted for measurement of HS and DS). Quantification of HS and DS was performed as described previously using highly sensitive liquid chromatography-tandem mass spectrometry (LC-MS/MS).[32] Internal standards for the analysis of HS and DS were obtained by deuterio-methanolysis of HS and DS. In brief, 2 M HCL-methanol-d4 was prepared by dropwise addition of 96 μL of HCl to 600 μL of methanol-d4 (Sigma, 441348) in an ice bath; 0.58 mL of 2 M HCL-methanol-d4 was used to methanolyse 600 μg of HS and DS in the presence of 25 μL of 2,2-dimethoxypropane for 75 min at 65°C. Deuterio-methanolysed HS and DS were dried in borosilicate tubes under nitrogen and resuspended in 1 mL of 10 mM ammonium acetate in 90:10% (v/v) acetonitrile/water.

An internal standard working solution was prepared by 25-fold dilution of HS and DS internal standards in 10 mM ammonium acetate in 90:10% (v/v) acetonitrile/water. Brain homogenates (125 μL) were transferred into borosilicate tubes and dried under nitrogen. After addition of 25 μL of 2,2-dimethoxypropane and 300 μL of 3 M HCl-methanol (Merck, 90964), samples were incubated for 75 min at 65°C and dried under nitrogen. Samples were resuspended in 150 μL of internal standard working solution. For measurement of HS and DS, 13 μL of sample preparation was mixed with 187 μL 10 mM ammonium acetate in 90:10% (v/v) acetonitrile/water. LC-MS/MS was performed on a Sciex 5500 QTrap (tandem) mass spectrometer coupled to a Waters Acquity UPLC system. The linear range of the calibration curves were 0, 7.8125, 15.625, 31.25, 62.5, 125, 250, and 500 μg/mL of HS and DS dissolved in ultrapure water.

Histopathology and immunohistochemistry

After perfusion, brain was excised and the left hemisphere was fixed in methacarn (v/v – 60% absolute methanol, 30% chloroform, 10% glacial acetic acid), dehydrated in 50% and 70% ethanol for 24 h and processed in paraffin (histokinette). Brain was sectioned at 8 μm and either stained with Alcian blue or processed for immunohistological staining. For Alcian blue staining, brain sections were rehydrated and equilibrated in 0.1 N hydrochloric acid (Sigma) for 30 s, followed by staining in 1% Alcian blue 8GX (Sigma) pH 1 for 90 min. Sections were differentiated in 0.1 N hydrochloric acid (Sigma) for 30 s and stained in 0.1% nuclear fast red (Sigma) in 0.06 M aluminum sulfate hexadecahydrate (Sigma) for 5 min. Sections were rinsed in 95% ethanol, dehydrated, and mounted in Entellan mounting medium (Sigma). Alcian blue pathology in brain was either quantified (brain parenchyma) by counting the number of Alcian blue-positive cells or by scoring (choroid plexus and meninges) based on a scale from 1 to 4 by two independent operators blinded to the experimental and control groups. The scoring rules are described in Table S2. Immunohistochemical staining was performed on brains fixed in methacarn as described above. Sections (8 μm) were rehydrated and blocked for endogenous peroxidase in 3% hydrogen peroxidase (dilution 1:2 in dH_2O of a 6% v/v solution BMS-2110-1E, PHC Corporation). Endogenous avidin and biotin were blocked for 15 min at room temperature (RT) according to the manufacturer's instructions using an Avidin/Biotin blocking kit (Vector Laboratories, SP-2001), followed by blocking for 30 min in staining buffer (3% BSA, 3% goat serum, 0.3% Triton X-100 in PBS) at RT. Sections were stained with primary antibodies detecting astrocytes (rabbit anti-GFAP IgG, 1:500, Sigma-Aldrich, G9269), activated microglia (rat anti-CD68 IgG, 1:300 Bio-Rad, , MCA1957T), LAMP1 (rat anti-LAMP1,1:500, Abcam, ab25245), IDS (goat anti-human IDS biotinylated, 0.5 μg/mL, R&D Systems, BAF2449), or GFP (chicken anti-GFP, 1:1,000, Abcam, ab13970) in staining buffer O/N at 4°C. The day after, sections were incubated with goat anti-rat antibody biotinylated (to detect CD68 and LAMP1, 1:200, BD Pharmingen, 554014), anti-rabbit (to detect GFAP, 1:200, from the ABC kit PK6101 Vectastain), or goat anti-chicken (to detect GFP, 1:200, Invitrogen, A16058) in staining buffer for 60 min at RT. Sections were then incubated with streptavidin-HRP (1:50, R&D Systems, DY998) in staining buffer for 60 min at RT. Sections were finally incubated in impact DAB (SK-4105) for either 2 min (GFAP, CD68, LAMP1, and GFP) or 10 min (IDS). For IDS staining, sections were counterstained in 0.1% nuclear fast red (Sigma) in 0.06 M aluminum sulfate hexadecahydrate (Sigma) for 5 s. Sections were mounted in Entellan (Sigma) and scanned by a NanoZoomer 2.0 (Hamamatsu Photonics, Japan).

Flow cytometry analysis of chimerism

Flow cytometry analysis of chimerism in bone marrow was performed as described previously.[23] In brief, tibia and femur of the right hindlimb, as well as humerus bones, were isolated and flushed with 2% FCS in PBS to obtain the bone marrow. Bone marrow was then filtered with cell-strainer capped polystyrene tubes (Corning, 352235), centrifuged at 300 × *g* for 10 min at 4°C and lysed in RBC lysing buffer (BD Pharm Lyse, 555899) for 10 min at RT. Bone marrow cells were washed with PBS and frozen in 10% DMSO in FCS. When needed, cells were thawed at 37°C and washed in 2% FCS in PBS, resuspended in 45 μL of 4% FCS in PBS, and incubated at 4°C for 15 min. Staining was performed in 4% FCS in PBS with FITC-mouse anti-mouse CD45.2 (BD Bioscience, 553772) and PE-mouse anti-mouse CD45.1 (BD Bioscience, 553776). Every experiment was performed with single-staining controls, unstained controls, and isotype-stained controls (mouse IgG2a-FITC, BD Bioscience, 349051; mouse IgG2a-PE, 349053). Measurement of chimerism was performed using a BD LSRFortessa and FACS DIVA software recording 20,000 or more events per sample, while analysis was performed using FlowJo v.10.

qPCR of VCN and chimerism

VCN and chimerism in bone marrow, brain, and liver were determined by qPCR. Genomic DNA was extracted either with the NucleoSpin Tissue kit (bone marrow; Macherey-Nagel, Düren, Germany) or by EtOH precipitation (brain and liver). Genomic DNA (50 ng) were used to measure both chimerism and VCN using iTaq Universal SYBR Green Supermix (Bio-Rad, Hercules, CA). VCN was determined using primers specific for *PSI* (FW: 5′-CAGGAC TCGGCTTGCTGAAG; RV: 5′-TCCCCCGCTTAATACTGACG) and mouse *Albumin* (FW: 5′-ACTTTGAGTGTAGCA GAGAGGA ACC; RV: 5′-CTCTTCACTGACCTAAGCTACTCCC) and using a standard curve with logarithmic dilutions (10^9–10^2 copies in the reaction volume) of pCCL-MND-*IDSco* and pGEM-mAlbumin. The pGEM-mAlbumin plasmid was generated via PCR amplification (Phusion High-Fidelity DNA Polymerase, M0530L) of a fragment of the mouse *Albumin* gene (NC_000071.7: 90609165–90609760 Mus Musculus strain C57BL/6J chromosome 5, GRCm39) from genomic DNA of an FVB mouse using the primers FW: 5′-*T*TTAT TACGGTCTCATAGGGCCTGC and RV: 5′-GCACACATTTCTAC TGGACAGCAC. PCR product was then purified (QIAquick, 28106) and dephosphorylated (CIP NEB, M0290S) and cloned into a pGEM plasmid restricted with SacII (NEB) and SpeI (NEB), and blunted with Klenow (NEB, M0210). Chimerism was determined using primers specific for the *Cd45.1* allele (FW: 5′-CTGAGCCTGCATC TAAACCTGATC; RV: 5′-TCACCTTCATAAAAGCCTTGT AGCT C). This allele differs from the *Cd45.2* allele for the presence of T>C (NC_000067.7: 138058514 Mus Musculus strain C57BL/6J

chromosome 1, GRCm39) and a GT>TC (NC_000067.7: 138058527–138058528 Mus Musculus strain C57BL/6J chromosome 1, GRCm39) point mutations. The annealing temperature for analysis of chimerism was 67.5°C, which resulted in specific amplification of the *Cd45.1* allele and no amplification of the *Cd45.2* allele. Standard for chimerism analysis was prepared by mixing CD45.1 genomes with CD45.2 genomes at different ratios (Figure S10). Reactions were performed and measured in a CFX96 real-time PCR detection system and analyzed by CFX Manager 3.0 (Bio-Rad, Hercules, CA).

Sandwich and functional ELISA

IDS sandwich ELISA was performed using the DuoSet kit according to the manufacturer's instructions (R&D Systems, DY449-05). For LRP-1 and IGF2R functional ELISA, a 96-well microplate (Nunc Sigma) was coated with 100 μL of goat anti-human IgG H + L (for LRP-1 functional ELISA; Invitrogen) or mouse anti-HIS tag antibody (R&D Systems, MAB050) at a working concentration of 8 μg/mL (anti-human IgG H + L) or 0.5 μg/mL (anti-HIS) in PBS at RT O/N. Each well was then washed 3 times with 0.05% Tween (Sigma) in PBS and blocked for 2 h at RT with 1% BSA (Sigma) in PBS. LRP-1 (LRP-1 Cluster IV Chimera Protein; R&D Systems, 5395-L4-050) or IGF2R (domain 11; R&D Systems, 2447-GR-050) were diluted in 1% BSA in PBS at a concentration of 1 μg/mL (LRP-1) or 0.8 μg/mL (IGF2R) and applied to coated wells for 2 h at RT. After washing, 100 μL of conditioned medium containing IDS-tagged versions at the concentration indicated in Figures 7I and 7J were applied to the coated wells for 2 h at RT. Two-fold or log-dilutions of conditioned medium were performed in complete medium as described above. IDS protein was detected by incubation with 100 μL of biotinylated goat anti-IDS antibody (IDS biotinylated, R&D Systems, BAF2449) at a concentration of 75 ng/mL for 2 h at RT and incubation with 100 μL of streptavidin-HRP (R&D Systems, DY998). After incubation in 100 μL of substrate solution (R&D Systems, DY999) for 20 min at RT, reaction was stopped with 2N HCl and signal was measured using a microplate reader.

Statistics

Statistical analysis was performed using GraphPad Prism (v.9.0.0. for Windows, San Diego, CA, www.graphpad.com). All results are presented as mean ± SEM and each data point is shown. Normality tests were performed by Shapiro-Wilk test. Multiple comparison analysis was performed by one-way ANOVA with Bonferroni's correction. Lysosomal pathology and neuroinflammation quantification in brain was analyzed by two-way ANOVA with Bonferroni's correction using brain area and viral vector as categorical variables. Non-linear regression models were used to describe the relationship between HS and other variables, such as VCN, IDS activity in brain, and IDS activity in plasma, as well as the relationship between VCN and activity in bone marrow, and signal and input concentration during functional ELISA. This analysis was performed using GraphPad Prism built-in models, such as one-phase decay and Michaelis-Menten. Linear regression analysis was performed using GraphPad Prism.

DATA AND CODE AVAILABILITY

Data are available on request.

SUPPLEMENTAL INFORMATION

Supplemental information can be found online at https://doi.org/10.1016/j.omtm.2023.101149.

ACKNOWLEDGMENTS

This work was supported by the "Finding a cure for Hunter Disease" Foundation, the Sophia Foundation (grant S18-59), Metakids (grants 2018-083 and 2021-094), the Prinses Beatrix Spierfonds (grant W.OP20-04), the Hersenstichting (grant DR-2021-00386), Dioraphte (project 20210367), and the Dutch Research Agenda (NWA) grant "Cure4Life" from the Dutch Research Council (NWO). We thank Prof. Dr. Brian Bigger for kindly providing the transgenes *IDSco* and *IDS.ApoE2co*, and Hélène Gleitz for the technical advice. Fabio Catalano has been granted a license to use BioRender for the making of the graphical abstract (agreement no. KY25WFNHYP; created with biorender.com).

AUTHOR CONTRIBUTIONS

W.W.M.P.P. conceived the project. F.C. and W.W.M.P.P. designed the experiments. F.C., E.C.V., D.K., Z.D., and T.F.H. performed the experiments. All authors analyzed and interpreted the data. W.W.M.P.P. and A.T.v.d.P. obtained funding. F.C. and W.W.M.P.P. wrote the manuscript. All authors read and approved the final manuscript.

DECLARATION OF INTERESTS

A.T.v.d.P. has received consulting fees from Sanofi Genzyme and has provided consulting services, participated in advisory board meetings and received grants for premarketing studies and research from industries via agreements between Erasmus MC and the industry. W.W.M.P.P. and A.T.v.d,P. are advisors of LentiCure B.V., a company for the development of lentiviral gene therapy. F.C., E.C.V., A.T.v.d.P., and W.W.M.P.P. are inventors on patents in the field of lentiviral gene therapy.

REFERENCES

1. Hunter, C. (1917). A Rare Disease in Two Brothers. Proc. Roy. Soc. Med. *10*, 104–116. https://doi.org/10.1177/003591571701001833.
2. Bach, G., Eisenberg, F., Cantz, M., and Neufeld, E.F. (1973). The defect in the Hunter syndrome: deficiency of sulfoiduronate sulfatase. Proc. Natl. Acad. Sci. USA *70*, 2134–2138. https://doi.org/10.1073/PNAS.70.7.2134.
3. Wilson, P.J., Morris, C.P., Anson, D.S., Occhiodoro, T., Bielicki, J., Clements, P.R., and Hopwood, J.J. (1990). Hunter syndrome: isolation of an iduronate-2-sulfatase cDNA clone and analysis of patient DNA. Proc. Natl. Acad. Sci. USA *87*, 8531–8535. https://doi.org/10.1073/PNAS.87.21.8531.
4. Wilson, P.J., Meaney, C.A., Hopwood, J.J., and Morris, C.P. (1993). Sequence of the Human Iduronate 2-Sulfatase (IDS) Gene. Genomics *17*, 773–775. https://doi.org/10.1006/GENO.1993.1406.
5. D'avanzo, F., Rigon, L., Zanetti, A., and Tomanin, R. (2020). Mucopolysaccharidosis Type II: One Hundred Years of Research, Diagnosis, and Treatment. Int. J. Mol. Sci. *21*, 1258. https://doi.org/10.3390/IJMS21041258.
6. Tomita, K., Okamoto, S., Seto, T., and Hamazaki, T. (2021). Real world long-term outcomes in patients with mucopolysaccharidosis type II: A retrospective cohort study. Mol. Genet. Metab. Rep. *29*, 100816. https://doi.org/10.1016/J.YMGMR.2021.100816.
7. Muenzer, J., Botha, J., Harmatz, P., Giugliani, R., Kampmann, C., and Burton, B.K. (2021). Evaluation of the long-term treatment effects of intravenous idursulfase in patients with mucopolysaccharidosis II (MPS II) using statistical modeling: data from

the Hunter Outcome Survey (HOS). Orphanet J. Rare Dis. *16*, 456. https://doi.org/10.1186/S13023-021-02052-4.

8. Ullman, J.C., Arguello, A., Getz, J.A., Bhalla, A., Mahon, C.S., Wang, J., Giese, T., Bedard, C., Kim, D.J., Blumenfeld, J.R., et al. (2020). Brain delivery and activity of a lysosomal enzyme using a blood-brain barrier transport vehicle in mice. Sci. Transl. Med. *12*, eaay1163. https://doi.org/10.1126/SCITRANSLMED.AAY1163.

9. Arguello, A., Mahon, C.S., Calvert, M.E.K., Chan, D., Dugas, J.C., Pizzo, M.E., Thomsen, E.R., Chau, R., Damo, L.A., Duque, J., et al. (2022). Molecular architecture determines brain delivery of a transferrin receptor-targeted lysosomal enzyme. J. Exp. Med. *219*, e20211057. https://doi.org/10.1084/JEM.20211057.

10. Laoharawee, K., Podetz-Pedersen, K.M., Nguyen, T.T., Evenstar, L.B., Kitto, K.F., Nan, Z., Fairbanks, C.A., Low, W.C., Kozarsky, K.F., and Mcivor, R.S. (2017). Prevention of Neurocognitive Deficiency in Mucopolysaccharidosis Type II Mice by Central Nervous System-Directed, AAV9-Mediated Iduronate Sulfatase Gene Transfer. Hum. Gene Ther. *28*, 626–638. https://doi.org/10.1089/HUM.2016.184.

11. Motas, S., Haurigot, V., Garcia, M., Marcó, S., Ribera, A., Roca, C., Sánchez, X., Sánchez, V., Molas, M., Bertolin, J., et al. (2016). CNS-directed gene therapy for the treatment of neurologic and somatic mucopolysaccharidosis type II (Hunter syndrome). JCI Insight *1*, e86696. https://doi.org/10.1172/JCI.INSIGHT.86696.

12. Hordeaux, J., Hinderer, C., Goode, T., Buza, E.L., Bell, P., Calcedo, R., Richman, L.K., and Wilson, J.M. (2018). Toxicology Study of Intra-Cisterna Magna Adeno-Associated Virus 9 Expressing Iduronate-2-Sulfatase in Rhesus Macaques. Mol. Ther. Methods Clin. Dev. *10*, 68–78. https://doi.org/10.1016/J.OMTM.2018.06.004.

13. Bougnères, P., Hacein-Bey-Abina, S., Labik, I., Adamsbaum, C., Castaignède, C., Bellesme, C., and Schmidt, M. (2021). Long-Term Follow-Up of Hematopoietic Stem-Cell Gene Therapy for Cerebral Adrenoleukodystrophy. Hum. Gene Ther. *32*, 1260–1269. https://doi.org/10.1089/HUM.2021.053.

14. Kohn, D.B., Booth, C., Shaw, K.L., Xu-Bayford, J., Garabedian, E., Trevisan, V., Carbonaro-Sarracino, D.A., Soni, K., Terrazas, D., Snell, K., et al. (2021). Autologous Ex Vivo Lentiviral Gene Therapy for Adenosine Deaminase Deficiency. N. Engl. J. Med. *384*, 2002–2013. https://doi.org/10.1056/NEJMOA2027675.

15. Tucci, F., Scaramuzza, S., Aiuti, A., and Mortellaro, A. (2021). Update on Clinical Ex Vivo Hematopoietic Stem Cell Gene Therapy for Inherited Monogenic Diseases. Mol. Ther. *29*, 489–504. https://doi.org/10.1016/J.YMTHE.2020.11.020.

16. Fumagalli, F., Calbi, V., Natali Sora, M.G., Sessa, M., Baldoli, C., Rancoita, P.M.V., Ciotti, F., Sarzana, M., Fraschini, M., Zambon, A.A., et al. (2022). Lentiviral haematopoietic stem-cell gene therapy for early-onset metachromatic leukodystrophy: long-term results from a non-randomised, open-label, phase 1/2 trial and expanded access. Lancet *399*, 372–383. https://doi.org/10.1016/S0140-6736(21)02017-1.

17. Reinhardt, B., Habib, O., Shaw, K.L., Garabedian, E., Carbonaro-Sarracino, D.A., Terrazas, D., Fernandez, B.C., De Oliveira, S., Moore, T.B., Ikeda, A.K., et al. (2021). Long-term outcomes after gene therapy for adenosine deaminase severe combined immune deficiency. Blood *138*, 1304–1316. https://doi.org/10.1182/BLOOD.2020010260.

18. Capotondo, A., Milazzo, R., Garcia-Manteiga, J.M., Cavalca, E., Montepeloso, A., Garrison, B.S., Peviani, M., Rossi, D.J., and Biffi, A. (2017). Intracerebroventricular delivery of hematopoietic progenitors results in rapid and robust engraftment of microglia-like cells. Sci. Adv. *3*, e1701211. https://doi.org/10.1126/SCIADV.1701211.

19. Plasschaert, R.N., DeAndrade, M.P., Hull, F., Nguyen, Q., Peterson, T., Yan, A., Loperfido, M., Baricordi, C., Barbarossa, L., Yoon, J.K., et al. (2022). High-throughput analysis of hematopoietic stem cell engraftment after intravenous and intracerebroventricular dosing. Mol. Ther. *30*, 3209–3225. https://doi.org/10.1016/J.YMTHE.2022.05.022.

20. Wakabayashi, T., Shimada, Y., Akiyama, K., Higuchi, T., Fukuda, T., Kobayashi, H., Eto, Y., Ida, H., and Ohashi, T. (2015). Hematopoietic Stem Cell Gene Therapy Corrects Neuropathic Phenotype in Murine Model of Mucopolysaccharidosis Type II. Hum. Gene Ther. *26*, 357–366. https://doi.org/10.1089/HUM.2014.158.

21. Miwa, S., Watabe, A.M., Shimada, Y., Higuchi, T., Kobayashi, H., Fukuda, T., Kato, F., Ida, H., and Ohashi, T. (2020). Efficient engraftment of genetically modified cells is necessary to ameliorate central nervous system involvement of murine model of mucopolysaccharidosis type II by hematopoietic stem cell targeted gene therapy. Mol. Genet. Metabol. *130*, 262–273. https://doi.org/10.1016/J.YMGME.2020.06.007.

22. Smith, M.C., Belur, L.R., Karlen, A.D., Erlanson, O., Podetz-Pedersen, K.M., McKenzie, J., Detellis, J., Gagnidze, K., Parsons, G., Robinson, N., et al. (2022). Phenotypic Correction of Murine Mucopolysaccharidosis Type II by Engraftment of Ex Vivo Lentiviral Vector-Transduced Hematopoietic Stem and Progenitor Cells. Hum. Gene Ther. *33*, 1279–1292. https://doi.org/10.1089/HUM.2022.141.

23. Gleitz, H.F., Liao, A.Y., Cook, J.R., Rowlston, S.F., Forte, G.M., D'Souza, Z., O'Leary, C., Holley, R.J., and Bigger, B.W. (2018). Brain-targeted stem cell gene therapy corrects mucopolysaccharidosis type II via multiple mechanisms. EMBO Mol. Med. *10*, e8730. https://doi.org/10.15252/EMMM.201708730.

24. Cartier, N., Hacein-Bey-Abina, S., Bartholomae, C.C., Veres, G., Schmidt, M., Kutschera, I., Vidaud, M., Abel, U., Dal-Cortivo, L., Caccavelli, L., et al. (2009). Hematopoietic stem cell gene therapy with a lentiviral vector in X-linked adrenoleukodystrophy. Science *326*, 818–823. https://doi.org/10.1126/SCIENCE.1171242.

25. Liang, Q., Catalano, F., Vlaar, E.C., Pijnenburg, J.M., Stok, M., van Helsdingen, Y., Vulto, A.G., van der Ploeg, A.T., van Til, N.P., and Pijnappel, W.W.M.P. (2022). IGF2-tagging of GAA promotes full correction of murine Pompe disease at a clinically relevant dosage of lentiviral gene therapy. Mol. Ther. Methods Clin. Dev. *27*, 109–130. https://doi.org/10.1016/J.OMTM.2022.09.010.

26. Ruan, H., Chai, Z., Shen, Q., Chen, X., Su, B., Xie, C., Zhan, C., Yao, S., Wang, H., Zhang, M., et al. (2018). A novel peptide ligand RAP12 of LRP1 for glioma targeted drug delivery. J. Contr. Release *279*, 306–315. https://doi.org/10.1016/J.JCONREL.2018.04.035.

27. Böckenhoff, A., Cramer, S., Wölte, P., Knieling, S., Wohlenberg, C., Gieselmann, V., Galla, H.-J., and Matzner, U. (2014). Comparison of Five Peptide Vectors for Improved Brain Delivery of the Lysosomal Enzyme Arylsulfatase A. J. Neurosci. *34*, 3122–3129. https://doi.org/10.1523/JNEUROSCI.4785-13.2014.

28. Robbins, P.B., Skelton, D.C., Yu, X.J., Halene, S., Leonard, E.H., and Kohn, D.B. (1998). Consistent, persistent expression from modified retroviral vectors in murine hematopoietic stem cells. Proc. Natl. Acad. Sci. USA *95*, 10182–10187. https://doi.org/10.1073/pnas.95.17.10182.

29. Froissart, R., Millat, G., Mathieu, M., Bozon, D., and Maire, I. (1995). Processing of iduronate 2-sulphatase in human fibroblasts. Biochem. J. *309*, 425–430. https://doi.org/10.1042/BJ3090425.

30. Chen, B.H., Marioni, R.E., Colicino, E., Peters, M.J., Ward-Caviness, C.K., Tsai, P.-C., Roetker, N.S., Just, A.C., Demerath, E.W., Guan, W., et al. (1999). Targeted disruption of the mouse iduronate sulfatase gene. Am. J. Hum. Genet. *65*, A427. https://doi.org/10.1016/j.atherosclerosis.2011.04.015.

31. Garcia, A.R., Pan, J., Lamsa, J.C., and Muenzer, J. (2007). The characterization of a murine model of mucopolysaccharidosis II (Hunter syndrome). J. Inherit. Metab. Dis. *30*, 924–934. https://doi.org/10.1007/S10545-007-0641-8.

32. Tanaka, N., Kida, S., Kinoshita, M., Morimoto, H., Shibasaki, T., Tachibana, K., and Yamamoto, R. (2018). Evaluation of cerebrospinal fluid heparan sulfate as a biomarker of neuropathology in a murine model of mucopolysaccharidosis type II using high-sensitivity LC/MS/MS. Mol. Genet. Metabol. *125*, 53–58. https://doi.org/10.1016/J.YMGME.2018.07.013.

33. Jurga, A.M., Paleczna, M., and Kuter, K.Z. (2020). Overview of General and Discriminating Markers of Differential Microglia Phenotypes. Front. Cell. Neurosci. *14*, 198. https://doi.org/10.3389/FNCEL.2020.00198.

34. Lier, J., Streit, W.J., and Bechmann, I. (2021). Beyond Activation: Characterizing Microglial Functional Phenotypes. Cells *10*, 2236. https://doi.org/10.3390/CELLS10092236.

35. Biffi, A., Montini, E., Lorioli, L., Cesani, M., Fumagalli, F., Plati, T., Baldoli, C., Martino, S., Calabria, A., Canale, S., et al. (2013). Lentiviral Hematopoietic Stem Cell Gene Therapy Benefi ts Metachromatic Leukodystrophy. Science *341*, 1233158. https://doi.org/10.1126/science.1233158.

36. Cartier, N., Lewis, C.-A., Zhang, R., and Rossi, F.M. (2014). The role of microglia in human disease: therapeutic tool or target? Acta Neuropathol. *128*, 363–380. https://doi.org/10.1007/s00401-014-1330-y.

37. Neumann, H. (2006). Microglia: a cellular vehicle for CNS gene therapy. J. Clin. Invest. *116*, 2857–2860. https://doi.org/10.1172/JCI30230.

38. Shibuya, Y., Kumar, K.K., Mader, M.M.D., Yoo, Y., Ayala, L.A., Zhou, M., Mohr, M.A., Neumayer, G., Kumar, I., Yamamoto, R., et al. (2022). Treatment of a genetic brain disease by CNS-wide microglia replacement. Sci. Transl. Med. *14*, 9945.

39. Cuadros, M.A., Sepulveda, M.R., Martin-Oliva, D., Marín-Teva, J.L., and Neubrand, V.E. (2022). Microglia and Microglia-Like Cells: Similar but Different. Front. Cell. Neurosci. *16*, 33. https://doi.org/10.3389/FNCEL.2022.816439.

40. Capotondo, A., Milazzo, R., Politi, L.S., Quattrini, A., Palini, A., Plati, T., Merella, S., Nonis, A., Di Serio, C., Montini, E., et al. (2012). Brain conditioning is instrumental for successful microglia reconstitution following hematopoietic stem cell transplantation. Proc. Natl. Acad. Sci. USA *109*, 15018–15023. https://doi.org/10.1073/pnas.1205858109.

41. Maga, J.A., Zhou, J., Kambampati, R., Peng, S., Wang, X., Bohnsack, R.N., Thomm, A., Golata, S., Tom, P., Dahms, N.M., et al. (2013). Glycosylation-independent lysosomal targeting of acid α-glucosidase enhances muscle glycogen clearance in pompe mice. J. Biol. Chem. *288*, 1428–1438. https://doi.org/10.1074/JBC.M112.438663.

42. Prill, H., Luu, A., Yip, B., Holtzinger, J., Lo, M.J., Christianson, T.M., Yogalingam, G., Aoyagi-Scharber, M., LeBowitz, J.H., Crawford, B.E., and Lawrence, R. (2019). Differential Uptake of NAGLU-IGF2 and Unmodified NAGLU in Cellular Models of Sanfilippo Syndrome Type B. Mol. Ther. Methods Clin. Dev. *14*, 56–63. https://doi.org/10.1016/J.OMTM.2019.05.008.

43. Yogalingam, G., Luu, A.R., Prill, H., Lo, M.J., Yip, B., Holtzinger, J., Christianson, T., Aoyagi-Scharber, M., Lawrence, R., Crawford, B.E., and LeBowitz, J.H. (2019). BMN 250, a fusion of lysosomal alpha-N-acetylglucosaminidase with IGF2, exhibits different patterns of cellular uptake into critical cell types of Sanfilippo syndrome B disease pathogenesis. PLoS One *14*, e0207836. https://doi.org/10.1371/JOURNAL.PONE.0207836.

44. Reinhardt, R.R., and Bondy, C.A. (1994). Insulin-like growth factors cross the blood-brain barrier. Endocrinology *135*, 1753–1761. https://doi.org/10.1210/ENDO.135.5.7525251.

45. Duffy, K.R., Pardridge, W.M., and Rosenfeld, R.G. (1988). Human blood-brain barrier insulin-like growth factor receptor. Metabolism *37*, 136–140. https://doi.org/10.1016/S0026-0495(98)90007-5.

46. Fernandez, A.M., and Torres-Alemán, I. (2012). The many faces of insulin-like peptide signalling in the brain. Nat. Rev. Neurosci. *13*, 225–239. https://doi.org/10.1038/NRN3209.

47. Benarroch, E.E. (2012). Insulin-like growth factors in the brain and their potential clinical implications. Neurology *79*, 2148–2153. https://doi.org/10.1212/WNL.0B013E3182752EEF.

48. Urayama, A., Grubb, J.H., Sly, W.S., and Banks, W.A. (2008). Mannose 6-Phosphate Receptor–mediated Transport of Sulfamidase Across the Blood–brain Barrier in the Newborn Mouse. Mol. Ther. *16*, 1261–1266. https://doi.org/10.1038/MT.2008.84.

49. Urayama, A., Grubb, J.H., Sly, W.S., and Banks, W.A. (2004). Developmentally regulated mannose 6-phosphate receptor-mediated transport of a lysosomal enzyme across the blood-brain barrier. Proc. Natl. Acad. Sci. USA *101*, 12658–12663. https://doi.org/10.1073/pnas.0405042101.

50. Urayama, A. (2013). Toward the successful delivery of lysosomal enzymes across the blood-brain barrier. Clin. Exp. Neuroimmunol. *4*, 228–238. https://doi.org/10.1111/CEN3.12037.

51. Macháčková, K., Chrudinová, M., Radosavljević, J., Potalitsyn, P., Křížková, K., Fábry, M., Selicharová, I., Collinsová, M., Brzozowski, A.M., Žáková, L., et al. (2018). Converting Insulin-like Growth Factors 1 and 2 into High-Affinity Ligands for Insulin Receptor Isoform A by the Introduction of an Evolutionarily Divergent Mutation. Biochemistry *57*, 2373–2382. https://doi.org/10.1021/ACS.BIOCHEM.7B01260.

52. Carro, E., Nuñez, A., Busiguina, S., and Torres-Aleman, I. (2000). Circulating insulin-like growth factor I mediates effects of exercise on the brain. J. Neurosci. *20*, 2926–2933. https://doi.org/10.1523/JNEUROSCI.20-08-02926.2000.

53. Blyth, A.J., Kirk, N.S., and Forbes, B.E. (2020). Understanding IGF-II Action through Insights into Receptor Binding and Activation. Cells *9*, 2276. https://doi.org/10.3390/CELLS9102276.

54. Xu, Y., Kirk, N.S., Venugopal, H., Margetts, M.B., Croll, T.I., Sandow, J.J., Webb, A.I., Delaine, C.A., Forbes, B.E., and Lawrence, M.C. (2020). How IGF-II Binds to the Human Type 1 Insulin-like Growth Factor Receptor. Structure *28*, 786–798.e6. https://doi.org/10.1016/J.STR.2020.05.002.

55. de Meyts, P. (2015). Insulin/receptor binding: The last piece of the puzzle? Bioessays *37*, 389–397. https://doi.org/10.1002/BIES.201400190.

56. Kan, S.H., Aoyagi-Scharber, M., Le, S.Q., Vincelette, J., Ohmi, K., Bullens, S., Wendt, D.J., Christianson, T.M., Tiger, P.M.N., Brown, J.R., et al. (2014). Delivery of an enzyme-IGFII fusion protein to the mouse brain is therapeutic for mucopolysaccharidosis type IIIB. Proc. Natl. Acad. Sci. USA *111*, 14870–14875. https://doi.org/10.1073/PNAS.1416660111.

57. Xie, H., Chung, J.K., Mascelli, M.A., and McCauley, T.G. (2015). Pharmacokinetics and Bioavailability of a Therapeutic Enzyme (Idursulfase) in Cynomolgus Monkeys after Intrathecal and Intravenous Administration. PLoS One *10*, e0122453. https://doi.org/10.1371/JOURNAL.PONE.0122453.

58. Croy, J.E., Brandon, T., and Komives, E.A. (2004). Two Apolipoprotein E Mimetic Peptides, ApoE(130−149) and ApoE(141−155)2, Bind to LRP1. Biochemistry *43*, 7328–7335. https://doi.org/10.1021/BI036208P.

59. Oller-Salvia, B., Sánchez-Navarro, M., Giralt, E., and Teixidó, M. (2016). Blood-brain barrier shuttle peptides: an emerging paradigm for brain delivery. Chem. Soc. Rev. *45*, 4690–4707. https://doi.org/10.1039/C6CS00076B.

60. Minami, S.S., Cordova, A., Cirrito, J.R., Tesoriero, J.A., Babus, L.W., Davis, G.C., Dakshanamurthy, S., Turner, R.S., Pak, D.T., Rebeck, G.W., et al. (2010). ApoE mimetic peptide decreases A production *in vitro* and *in vivo*. Mol. Neurodegener. *5*, 16. https://doi.org/10.1186/1750-1326-5-16.

61. Wang, D., El-Amouri, S.S., Dai, M., Kuan, C.Y., Hui, D.Y., Brady, R.O., and Pan, D. (2013). Engineering a lysosomal enzyme with a derivative of receptor-binding domain of apoE enables delivery across the blood-brain barrier. Proc. Natl. Acad. Sci. USA *110*, 2999–3004. https://doi.org/10.1073/PNAS.1222742110.

62. Ahmed, S., Pande, A.H., and Sharma, S.S. (2022). Therapeutic potential of ApoE-mimetic peptides in CNS disorders: Current perspective. Exp. Neurol. *353*, 114051. https://doi.org/10.1016/J.EXPNEUROL.2022.114051.

63. Khan, A.I., Lu, Q., Du, D., Lin, Y., and Dutta, P. (2018). Quantification of Kinetic Rate Constants for Transcytosis of Polymeric Nanoparticle through Blood-Brain Barrier. Biochim. Biophys. Acta Gen. Subj. *1862*, 2779–2787. https://doi.org/10.1016/J.BBAGEN.2018.08.020.

64. Fda (2022). Cellular, Tissue, and Gene Therapies Advisory Committee June 9-10, 2022 Meeting Presentation- Elivaldogene Autotemcel (Eli-Cel): BLA 125755 Clinical Considerations for Efficacy and Specific Safety in Early Cerebral Adrenoleukodystrophy.

65. Fda (2022). Cellular, Tissue, and Gene Therapies Advisory Committee June 9, 2022-June 10, 2022 Meeting Briefing Document- FDA- 125755.

66. (2022). Bluebird bio Reports Second Quarter 2022 Financial Results and Highlights Operational Progress. https://www.sec.gov/Archives/edgar/data/1293971/000129397122000047/exhibit99120220804.htm.

67. (2022). Cellular, Tissue, and Gene Therapies Advisory Committee June 9-10, 2022 Meeting Announcement (FDA). https://www.fda.gov/advisory-committees/advisory-committee-calendar/cellular-tissue-and-gene-therapies-advisory-committee-june-9-10-2022-meeting-announcement-06092022#event-materials.

68. Cronk, J.C., Filiano, A.J., Louveau, A., Marin, I., Marsh, R., Ji, E., Goldman, D.H., Smirnov, I., Geraci, N., Acton, S., et al. (2018). Peripherally derived macrophages can engraft the brain independent of irradiation and maintain an identity distinct from microglia. J. Exp. Med. *215*, 1627–1647. https://doi.org/10.1084/jem.20180247.

69. Paul Chadarevian, J., Lombroso, S.I., Peet, G.C., Hasselmann, J., Tu, C., Marzan, D.E., Capocchi, J., Purnell, F.S., Nemec, K.M., Lahian, A., et al. (2022). Engineering an inhibitor-resistant human CSF1R variant for microglia replacement. J. Exp. Med. *220*, e20220857. https://doi.org/10.1084/jem.20220857.

70. Hohsfield, L.A., Najafi, A.R., Ghorbanian, Y., Soni, N., Hingco, E.E., Kim, S.J., Jue, A.D., Swarup, V., Inlay, M.A., and Green, K.N. (2020). Effects of long-term and brain-wide colonization of peripheral bone marrow-derived myeloid cells in the CNS. J. Neuroinflammation *17*, 279. https://doi.org/10.1186/S12974-020-01931-0.

71. Wilkinson, F.L., Sergijenko, A., Langford-Smith, K.J., Malinowska, M., Wynn, R.F., and Bigger, B.W. (2013). Busulfan Conditioning Enhances Engraftment of Hematopoietic Donor-derived Cells in the Brain Compared With Irradiation. Mol. Ther. *21*, 868–876. https://doi.org/10.1038/mt.2013.29.

72. Araya, K., Sakai, N., Mohri, I., Kagitani-Shimono, K., Okinaga, T., Hashii, Y., Ohta, H., Nakamichi, I., Aozasa, K., Taniike, M., and Ozono, K. (2009). Localized donor cells in brain of a Hunter disease patient after cord blood stem cell transplantation. Mol. Genet. Metabol. *98*, 255–263. https://doi.org/10.1016/J.YMGME.2009.05.006.

73. Muenzer, J., Lamsa, J.C., Garcia, A., Dacosta, J., Garcia, J., and Treco, D.A. (2002). Enzyme replacement therapy in mucopolysaccharidosis type II (Hunter syndrome): a preliminary report. Acta Paediatr. Suppl. *91*, 71–78. https://doi.org/10.1111/J.1651-2227.2002.TB03115.X.

74. Garcia-Perez, L., van Eggermond, M., van Roon, L., Vloemans, S.A., Cordes, M., Schambach, A., Rothe, M., Berghuis, D., Lagresle-Peyrou, C., Cavazzana, M., et al. (2020). Successful Preclinical Development of Gene Therapy for Recombinase-Activating Gene-1-Deficient SCID. Mol. Ther. Methods Clin. Dev. *17*, 666–682. https://doi.org/10.1016/J.OMTM.2020.03.016.

75. Wang, C.q., Yang, C.s., Yang, Y., Pan, F., He, L.y., and Wang, A.m. (2013). An apolipoprotein E mimetic peptide with activities against multidrug-resistant bacteria and immunomodulatory effects. J. Pept. Sci. *19*, 745–750. https://doi.org/10.1002/psc.2570.

76. Dull, T., Zufferey, R., Kelly, M., Mandel, R.J., Nguyen, M., Trono, D., and Naldini, L. (1998). A Third-Generation Lentivirus Vector with a Conditional Packaging System. J. Virol. *72*, 8463–8471.

77. Bergsma, A.J., in 't Groen, S.L.M., Catalano, F., Yamanaka, M., Takahashi, S., Okumiya, T., van der Ploeg, A.T., and Pijnappel, W.W.M.P. (2021). A generic assay for the identification of splicing variants that induce nonsense-mediated decay in Pompe disease. Eur. J. Hum. Genet. *29*, 422–433. https://doi.org/10.1038/s41431-020-00751-3.

78. Liang, Q., Vlaar, E.C., Catalano, F., Pijnenburg, J.M., Stok, M., van Helsdingen, Y., Pijnappel, W.W.M.P., van Til, N.P., Vulto, A.G., Unger, W.W.J., et al. (2022). Lentiviral gene therapy prevents anti-human acid α-glucosidase antibody formation in murine Pompe disease. Mol. Ther. Methods Clin. Dev. *25*, 520–532. https://doi.org/10.1016/J.OMTM.2022.04.016.

79. Voznyi, Y.V., Keulemans, J.L., and van Diggelen, O.P. (2001). A fluorimetric enzyme assay for the diagnosis of MPS II (hunter disease) *24*, 675–680. https://doi.org/10.1023/A:1012763026526.

OMTM, Volume *31*

Supplemental information

Tagged IDS causes efficient and engraftment-independent prevention of brain pathology during lentiviral gene therapy for Mucopolysaccharidosis type II

Fabio Catalano, Eva C. Vlaar, Drosos Katsavelis, Zina Dammou, Tessa F. Huizer, Jeroen C. van den Bosch, Marianne Hoogeveen-Westerveld, Hannerieke J.M.P. van den Hout, Esmeralda Oussoren, George J.G. Ruijter, Gerben Schaaf, Karin Pike-Overzet, Frank J.T. Staal, Ans T. van der Ploeg, and W.W.M. Pim Pijnappel

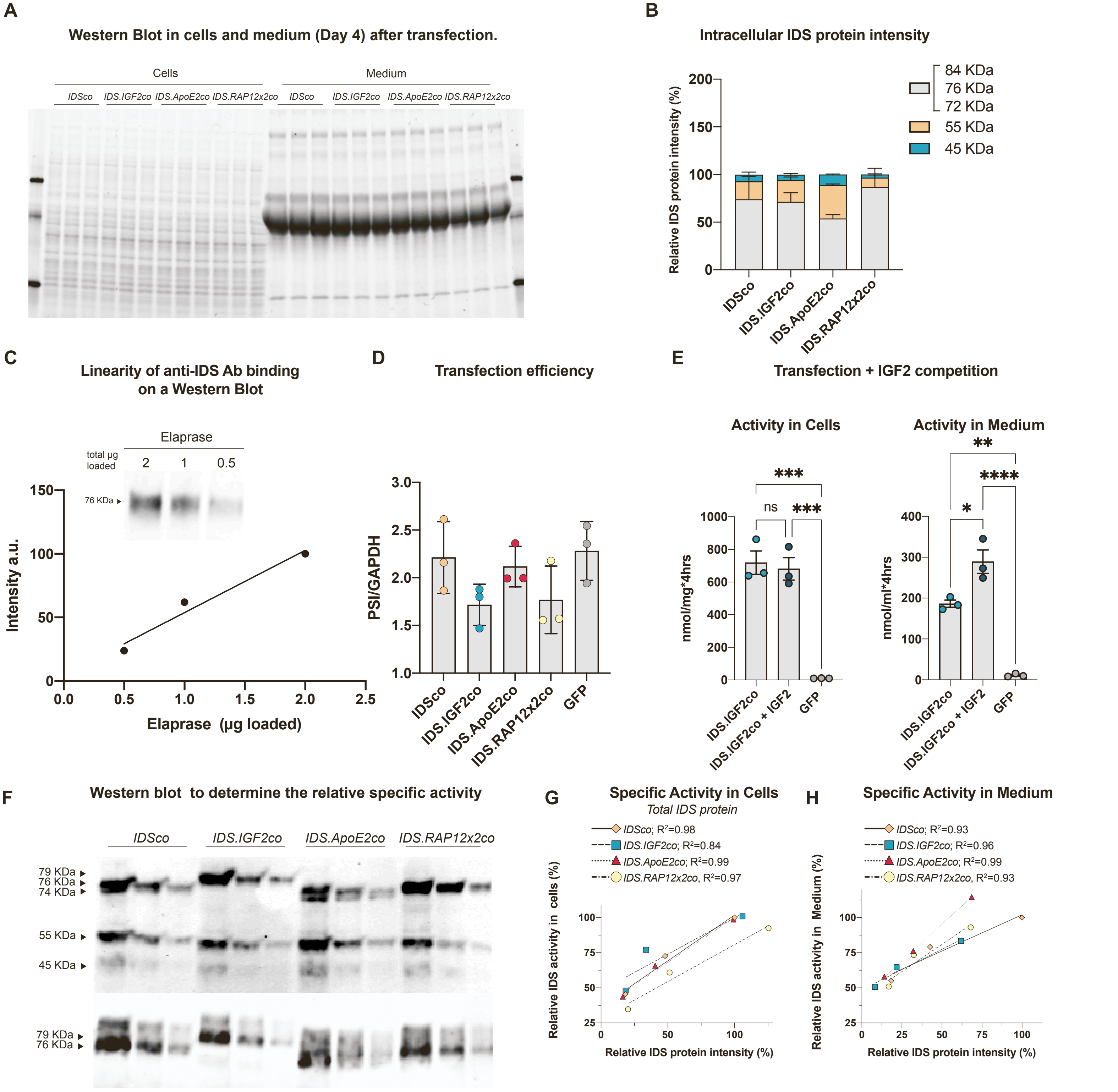

Figure S1. Control experiments for *in vitro* characterization of tagged IDS proteins.
(A) 20 µg total protein load for the western blot in Figure 1B. Equal loading was determined by quantification of the stain free signal of the same gel used for immunoblot analysis. Quantification of Figure 1B is shown in (B), where each processing form is expressed as relative of the total amount of protein. (C) Linearity of anti-IDS antibody binding on a western blot. (D) Transfection efficiency based on mRNA expression of *PSI* present in the lentiviral vector. (E) Intracellular and secreted IDS activity levels after transient transfection with *IDS.IGF2co* and addition or not of 1.5 µM IGF2 peptide in medium. (F) IDS protein levels measured in 3 2-fold dilutions of cell lysate and medium supernatant of transfected HEK 293T cells. Samples from Figure 1B were pooled and diluted to equal IDS activity levels before loading on gel. (G-H) IDS protein levels on western blot (Figure S1F) and IDS enzyme activity were measured in 3 2-fold dilutions of cell lysate and medium supernatant of transfected HEK 293T cells to determine the specific activity in cells (G) and medium (H). X-axes in G and H show the total IDS protein quantified from C. Slope and specific activity values are shown in Table S1. Data represent means ± SEM and were analyzed by one-way ANOVA followed by Bonferroni's multiple testing correction. *n = 3* biological replicates/condition. *p ≤ 0.05, **p ≤ 0.01, ***p ≤ 0.001, ****p ≤ 0.0001."

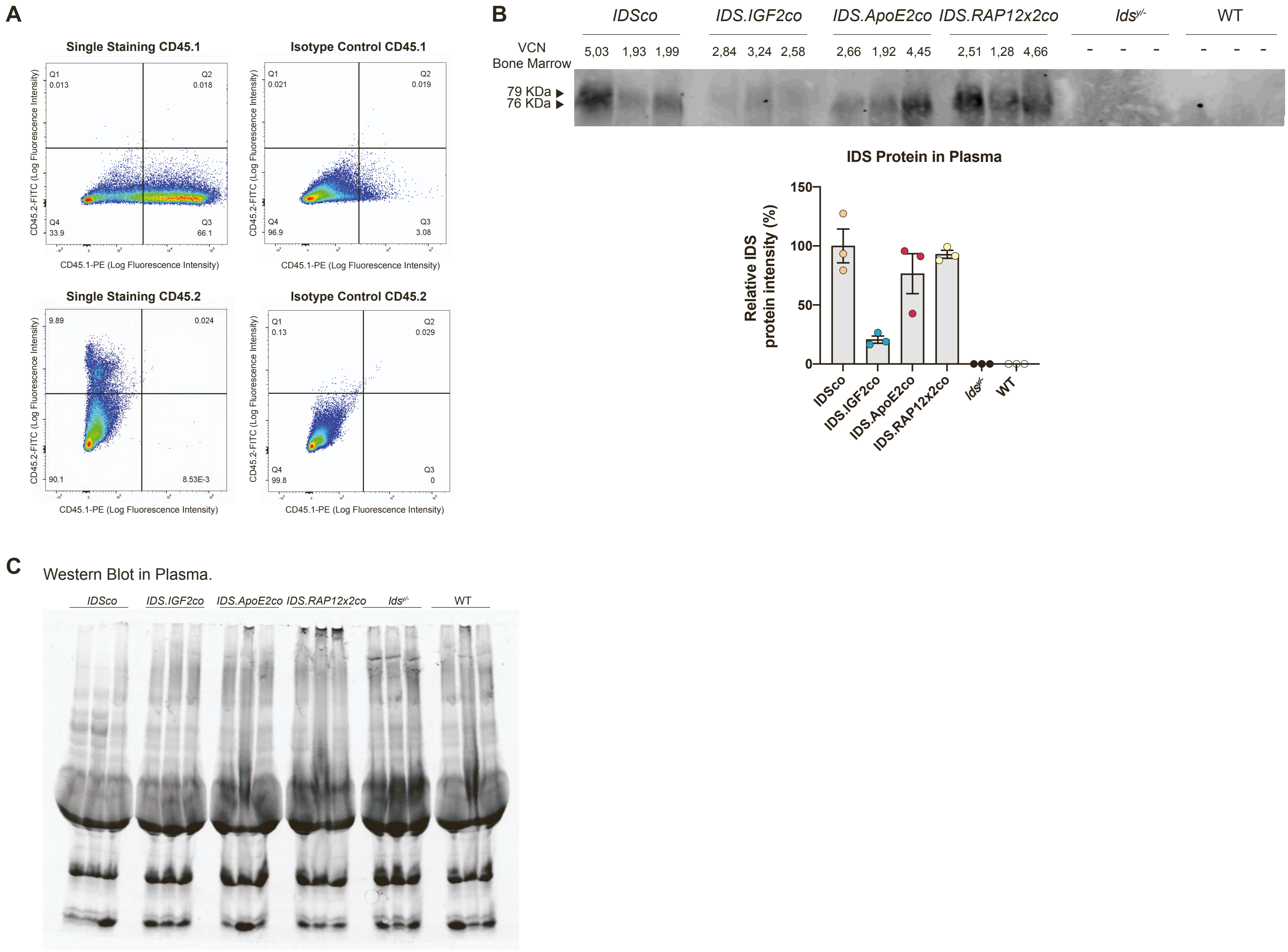

Figure S2. Chimerism control experiment and immunoblot of IDS protein in plasma.
(A) Single staining and isotype control staining of CD45.1 for measuring chimerism in bone marrow. (B) Immunoblot analysis of IDS protein in plasma after gene therapy. VCN in bone marrow for each sample is indicated. Quantification of IDS protein immunoblot in plasma is shown in the graph below. Equal loading was determined by quantification of the stain-free signal in (C), after loading of 276 µg of total protein. Data represent means ± SEM.

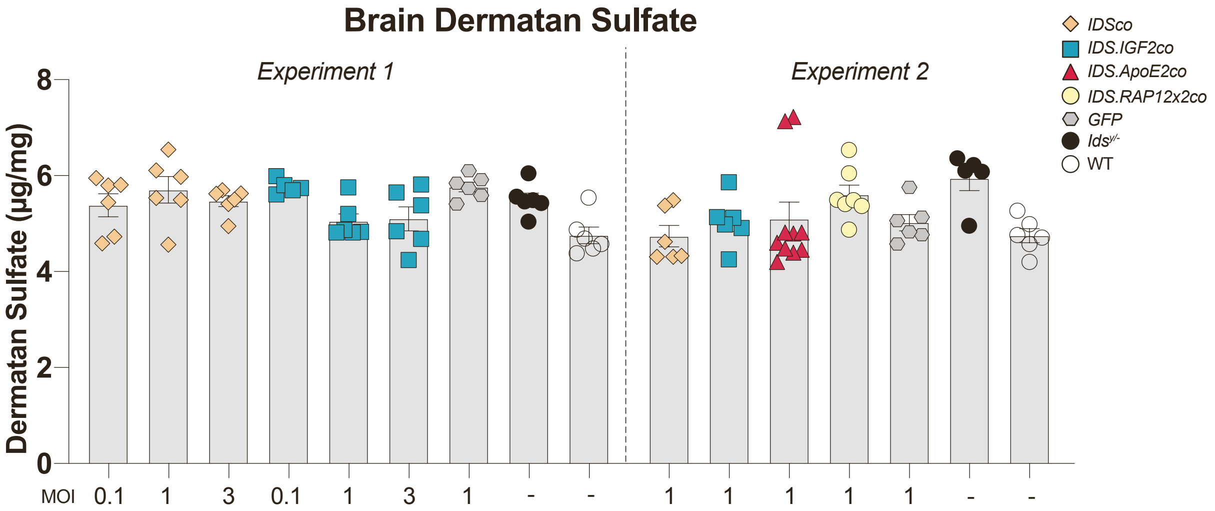

Figure S3. Quantification of brain dermatan Sulfate after gene therapy.
Mass spectrometry quantification of dermatan sulfate in brain homogenates of *Ids*$^{y/-}$ mice after gene therapy. Data are presented as means ± SEM and were analyzed by one-way ANOVA with Bonferroni's correction.

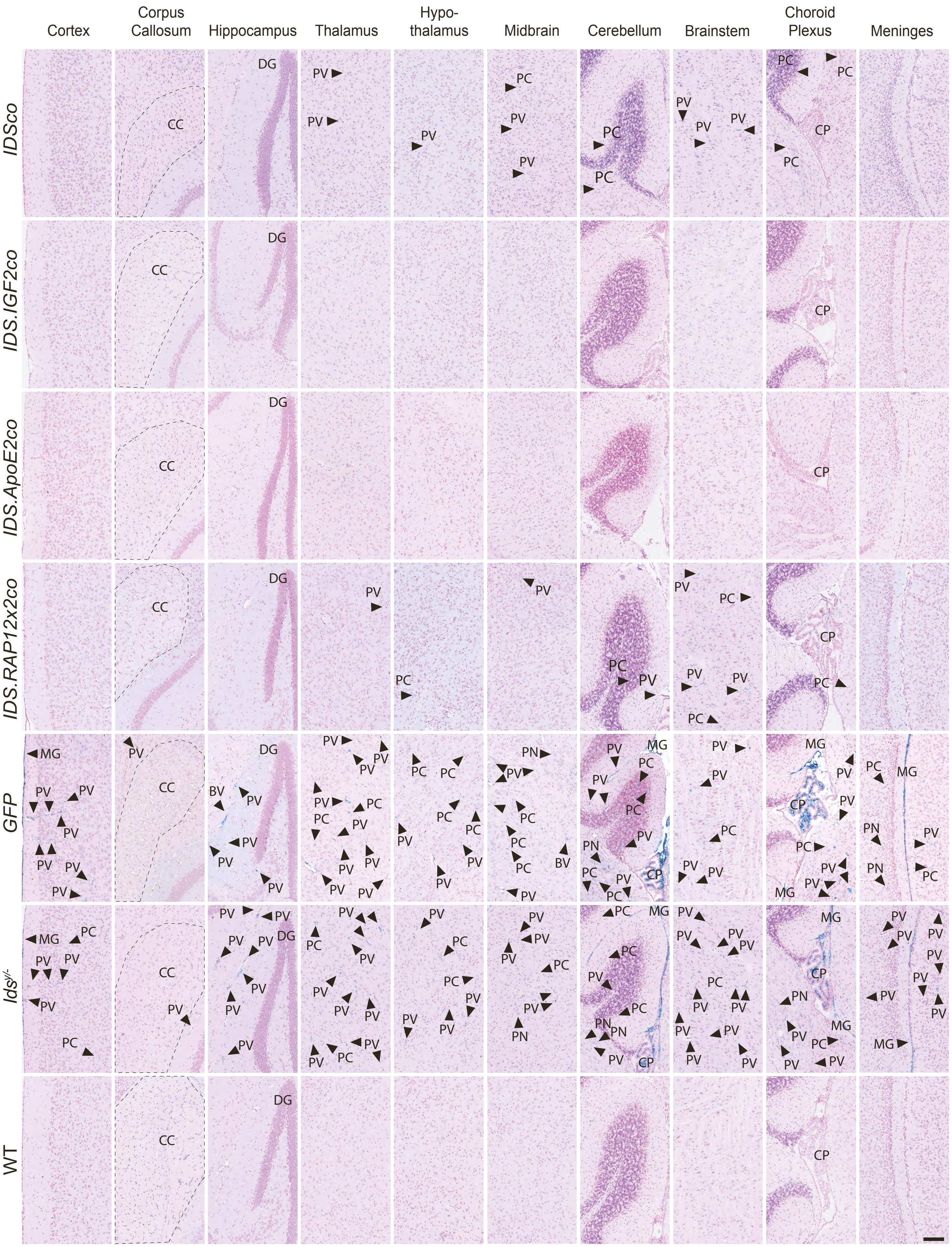

Figure S4. Alcian blue staining of *Ids*$^{y/-}$ brain after gene therapy

Alcian blue staining of sagittal sections of cortex, corpus callosum, hippocampus, thalamus, hypothalamus, midbrain, cerebellum, brainstem, choroid plexus and meninges of gene therapy treated *Ids*$^{y/-}$ mice. CC: corpus callosum; DG: dentate gyrus; BV: blood vessel; PC: parenchymal cell; CP: choroid plexus; Mg: meninges; PN: perineuronal net. *n = 3*. Scale bar = 100 µm.

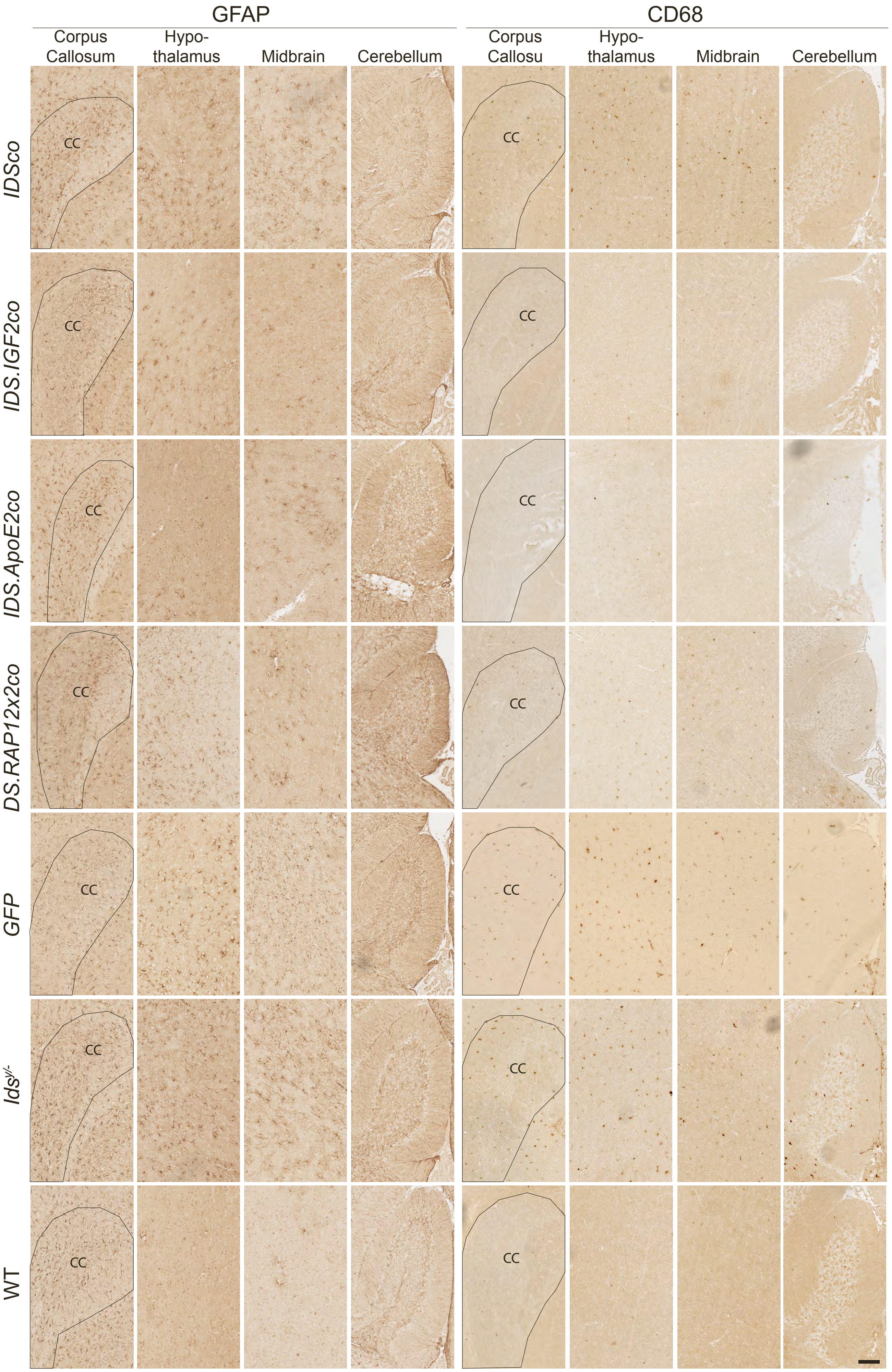

Figure S5. Gene therapy relieves neuroinflammation in corpus callosum, hypothalamus, midbrain and cerebellum. Sagittal sections of corpus callosum, hypothalamus, midbrain and cerebellum of gene therapy treated mice and controls stained for GFAP and CD68. CC: corpus callosum. *n = 3.* Scale bar = 100 µm.

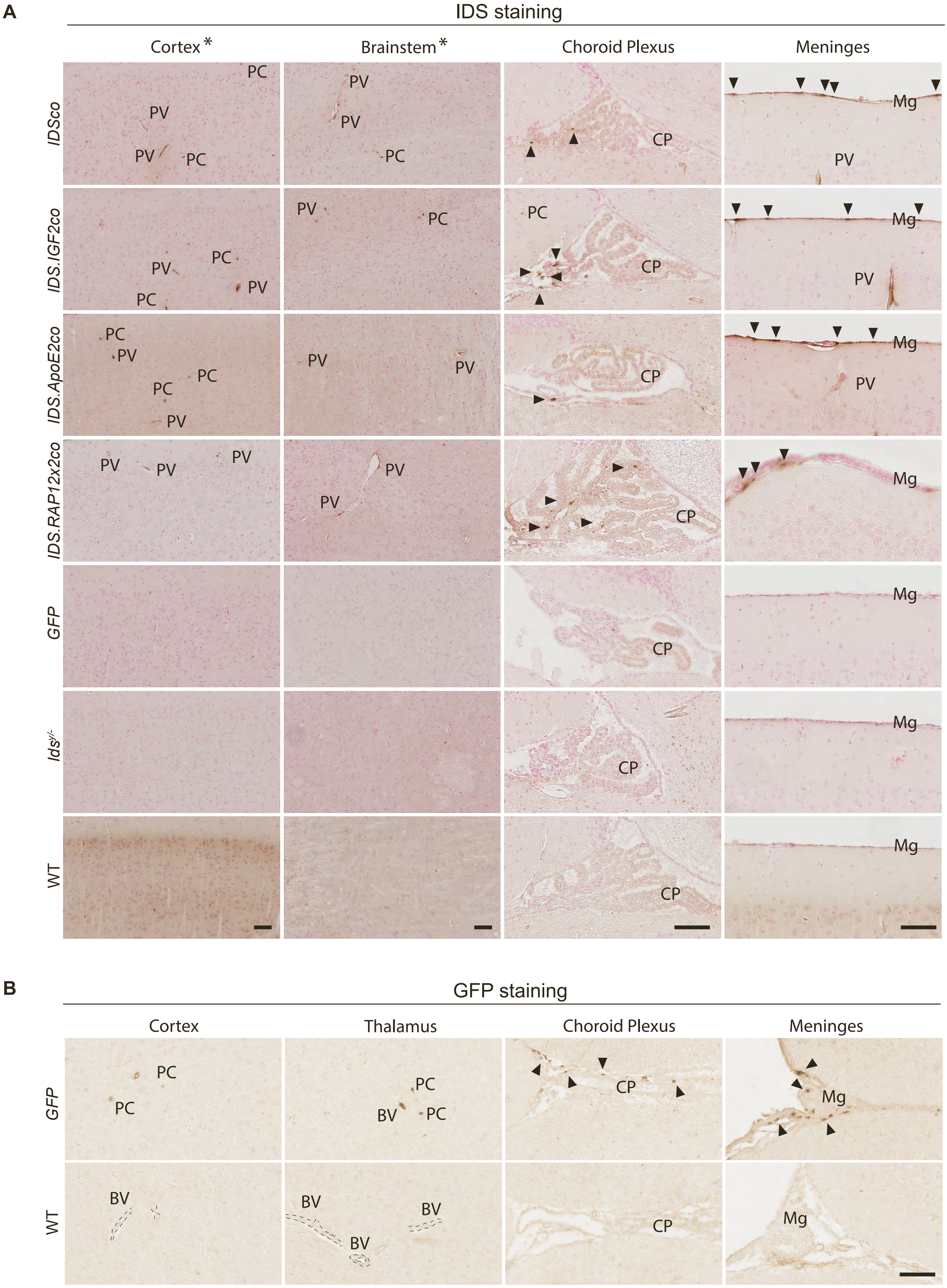

Figure S6. Engraftment of donor-derived cells in brain after gene therapy.
(A) Sagittal sections of cortex, brainstem, choroid plexus and meninges of gene therapy-treated and untreated *Ids*$^{y/-}$ mice and WT controls stained for IDS. Scale bar = 50 µm. (B) Sagittal sections of cortex, thalamus, choroid plexus and meninges stained for GFP. *n = 3*. Scale bar = 50 µm. BV: blood vessel; PC: parenchymal cell; PV: perivascular cells CP: choroid plexus; Mg: meninges. *Contrast was enhanced within the linear range in cortex and brainstem to highlight positive staining.

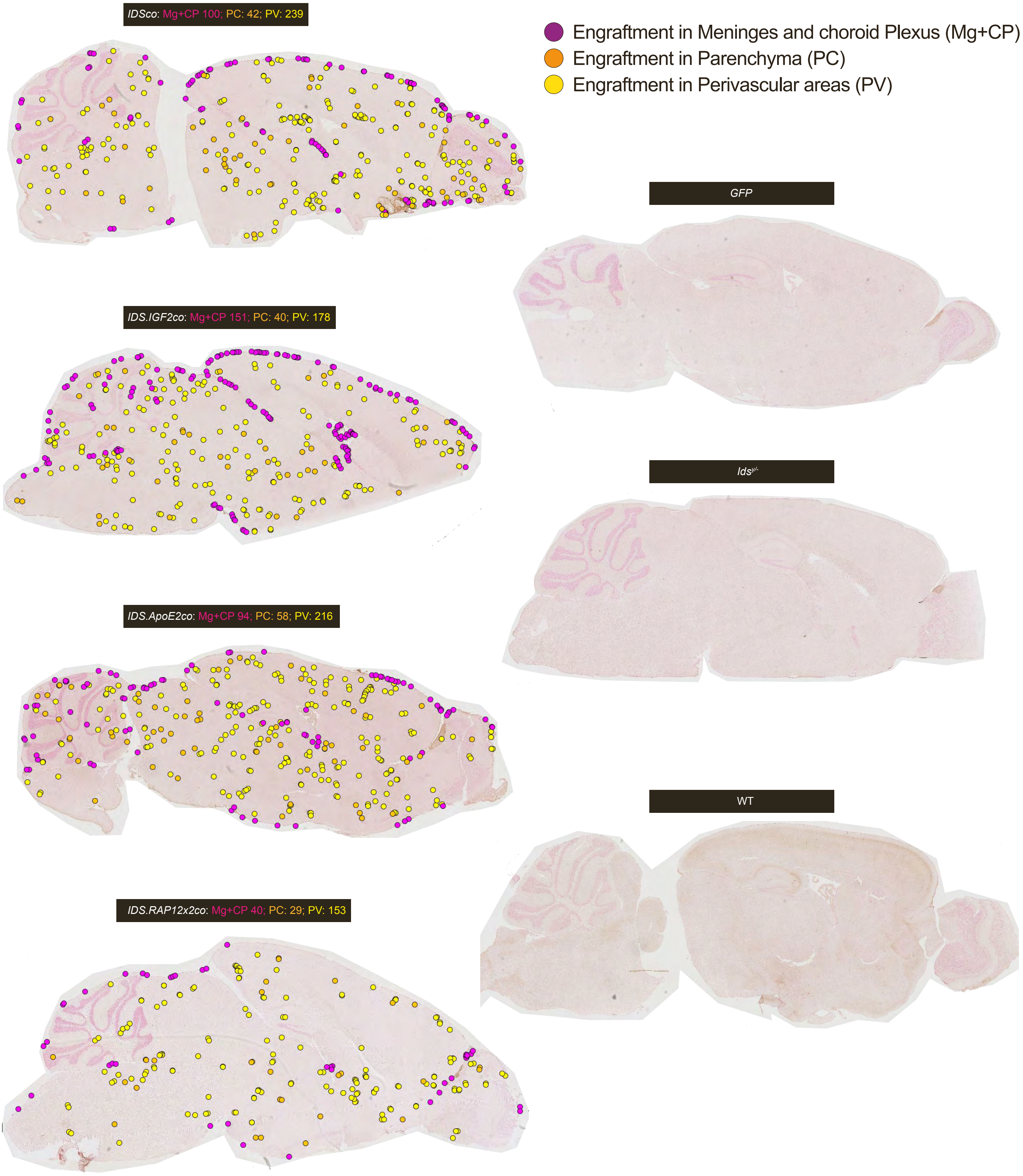

Figure S7. Distribution of IDS-positive cells in brain after gene therapy.
Examples of sagittal sections of gene therapy-treated and untreated *Ids*$^{y/-}$ mice and WT controls stained for IDS. IDS-positive cells are indicated by coloured dots based on the area of engraftment. Number of IDS-positive cells per brain area is indicated above each section.

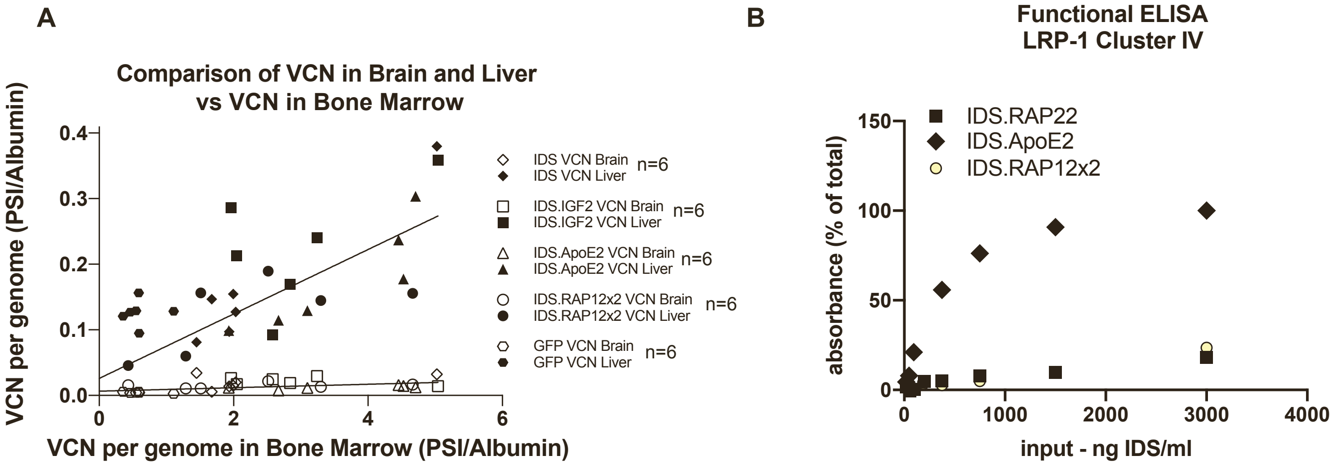

Figure S8. Comparison of VCN in liver and brain after gene therapy and LRP-1 ELISA for IDS.RAP22.
(A) Linear regression analysis between VCN in brain, VCN in liver and VCN in bone marrow. (B) Functional ELISA analysis of IDS.RAP22, IDS.ApoE2 and IDS.RAP12x2 proteins using the LRP-1 receptor (cluster IV).

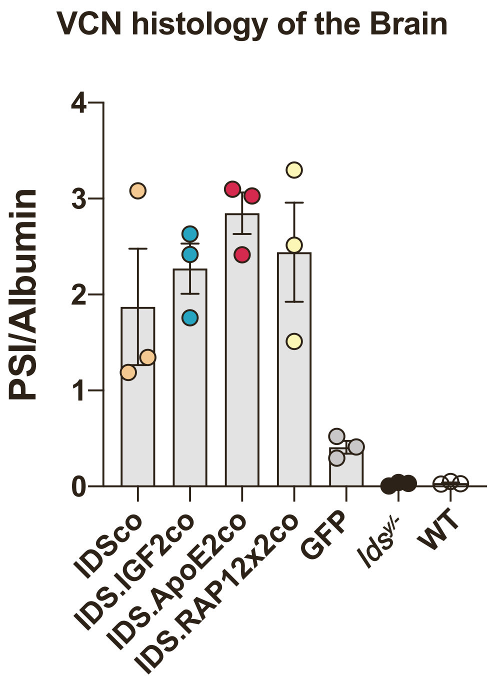

Figure S9. VCN in bone marrow of mice used for histology of the brain.
VCN in bone marrow of gene therapy-treated *Ids*$^{y/-}$ mice and control mice used for histology of the brain (Alcian Blue, LAMP1, GFAP, CD68 stainings). VCN per genome was measured by qPCR on *PSI* and *Albumin* loci. Data are presented as means ± SEM and were analyzed by one-way ANOVA with Bonferroni's correction.

A

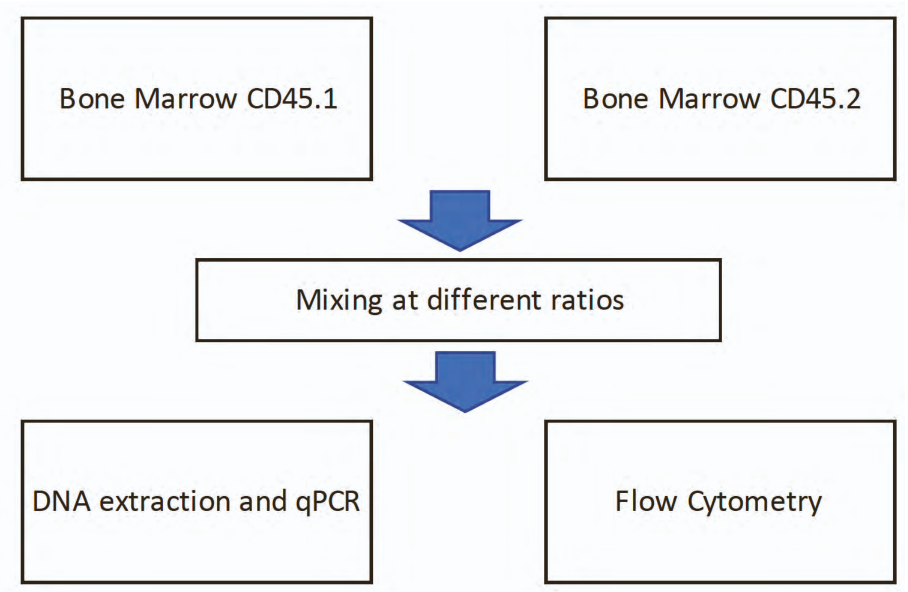

B

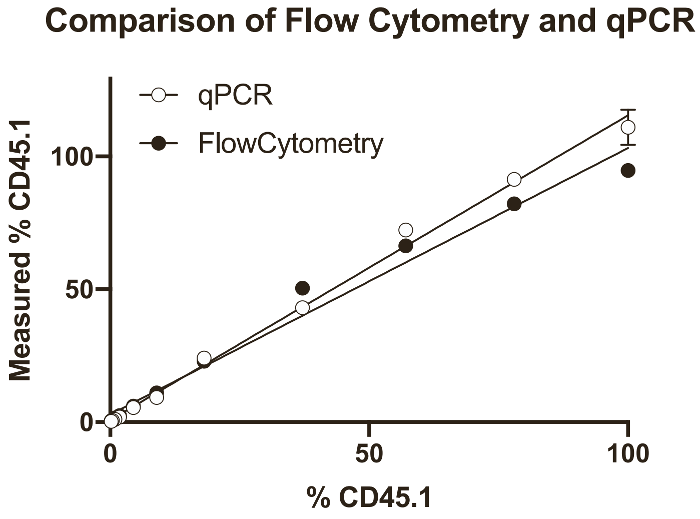

	qPCR	FlowCytometry
Goodness of Fit		
R squared	0.9958	0.9810

	qPCR	FlowCytometry
95% Confidence Intervals		
Slope	1.091 to 1.204	0.8984 to 1.109
Y-intercept	-1.744 to 3.201	-1.824 to 7.415
X-intercept	-2.882 to 1.475	-7.959 to 1.705

Figure S10. Validation of qPCR for the measurement of chimerism in brain and liver
(A) Whole bone marrows from CD45.1 and CD45.2 mice were resuspended at the same cellular density and mixed at the following ratios: 100 % (CD45.1 only); 80 % CD45.1 + 20 % CD45.2; 60 % CD45.1 + 40 % CD45.2; 40 % CD45.1 + 60 % CD45.2; 20 % CD45.1 + 80 % CD45.2; 10 % CD45.1 + 90 % CD45.2; 5 % CD45.1 + 95 % CD45.2; 2 % CD45.1 + 98 % CD45.2; 1 % CD45.1 + 99 % CD45.2; 0.5 % CD45.1 + 99.5 % CD45.2; 0.25 % CD45.1 + 99.75 % CD45.2. Each dilution was divided in 2 for flow cytometry and qPCR analysis. (B) Chimerism analysis of different CD45.1/CD45.2 bone marrow ratios samples measured by flow cytometry or allele specific qPCR on the Cd45.1 locus. 20 000 events were collected for flow cytometry analysis, while qPCR analysis was performed in technical triplicates. Data are presented as means ± SEM and were analyzed by one-way ANOVA with Bonferroni's correction.

Table S1 - Best fit & Confidence Intervals Values

Figure	Description of the experiment	Parameter	Description of the Parameter	Condition				
S1 G	Intracellular Specific Activity	Slope (nmol/(mg*a.u.)*4hrs)		***IDSco***	***IDS.IGF2co***	***IDS.ApoE2co***	***IDS.RAP12x2co***	***GFP***
			Best-fit values	0.6508	0.5176	0.6439	0.5255	N/A
			95% Confidence Intervals	-0.6291 to 1.931	-2.376 to 3.412	-0.3614 to 1.649	-0.6748 to 1.726	N/A
S1 H	Specific Activity in Medium	Slope (nmol/(ml*a.u.)*4hrs)		***IDSco***	***IDS.IGF2co***	***IDS.ApoE2***	***IDS.RAP12x2***	***GFP***
			Best-fit values	0.5165	0.5758	1.04	0.7757	N/A
			95% Confidence Intervals	-1.303 to 2.336	-0.8851 to 2.037	0.9373 to 1.143	-1.825 to 3.376	N/A
1 G	uptake into bEND.3 cells	EC50 Ratio IDS/Tag (a.u.)		***IDSco***	***IDS.IGF2co***	***IDS.ApoE2co***	***IDS.RAP12x2co***	***GFP***
			Best-fit values	1	0.2225	0.6666	0.983	5.168
			95% Confidence Intervals	N/A	0.1914 to 0.2537	0.5807 to 0.7545	0.8430 to 1.145	2.722 to ???
1 H	uptake into MPS II fibroblasts	EC50 Ratio IDS/Tag (a.u.)		***IDSco***	***IDS.IGF2co***	***IDS.ApoE2***	***IDS.RAP12x2***	***GFP***
			Best-fit values	1	0.2037	0.675	1.355	Unstable
			95% Confidence Intervals	N/A	0.1809 to 0.2269	0.6124 to 0.7384	1.205 to 1.537	(Very wide)
2 D	Activity in Bone Marrow vs VCN in Bone Marrow	Max IDS Activity (nmol/mg*4hrs)		***IDSco***	***IDS.IGF2co***	***IDS.ApoE2co***	***IDS.RAP12x2co***	***GFP***
			Best-fit values	4779	5064	6505	7090	N/A
			95% Confidence Intervals	3739 to 6420	4058 to 6977	4232 to 19028	4363 to 29549	N/A
		VCN 50% Max IDS Activity (VCN)		***IDSco***	***IDS.IGF2co***	***IDS.ApoE2co***	***IDS.RAP12x2co***	***GFP***
			Best-fit values	0.2772	0.8033	1.524	1.363	N/A
			95% Confidence Intervals	??? to 0.9174	0.2670 to 2.218	0.3340 to 11.42	0.2476 to 15.60	N/A
2 F	Activity in Plasma vs VCN in Bone Marrow	Slope (nmol/(mg*VCN)*4hrs)		***IDSco***	***IDS.IGF2co***	***IDS.ApoE2***	***IDS.RAP12x2***	***GFP***
			Best-fit values	29.15	11.88	29.75	30.08	N/A
			95% Confidence Intervals	13.51 to 44.80	8.127 to 15.63	19.60 to 39.90	13.87 to 46.29	N/A
3 C	Brain Heparan Sulfate vs VCN in Bone Marrow	Plateau (µg/mg HS)		***IDSco***	***IDS.IGF2co***	***IDS.ApoE2co***	***IDS.RAP12x2co***	***GFP***
			Best-fit values	1.161	0.4629	0.2153	1.253	N/A
			95% Confidence Intervals	1.060 to 1.257	0.3128 to 0.6098	0.1333 to 0.2971	0.8928 to 1.603	N/A
		λ, Exponential decay constant (1/VCN)		***IDSco***	***IDS.IGF2co***	***IDS.ApoE2co***	***IDS.RAP12x2co***	***GFP***
			Best-fit values	4.387	6.264	11.61	6.432	N/A
			95% Confidence Intervals	3.018 to ???	4.013 to 11.36	8.286 to ???	3.054 to 25.43	N/A
3 D	Brain Heparan Sulfate vs IDS activity in brain	Plateau (µg/mg HS)		***IDSco***	***IDS.IGF2co***	***IDS.ApoE2***	***IDS.RAP12x2***	***GFP***
			Best-fit values	1.078	0.4228	0.211	1.106	N/A
			95% Confidence Intervals	0.9645 to 1.177	0.3190 to 0.5262	0.1225 to 0.2949	0.5507 to 1.476	N/A
		λ, Exponential decay constant (1/(nmol/mg*4hrs))		***IDSco***	***IDS.IGF2co***	***IDS.ApoE2***	***IDS.RAP12x2***	***GFP***
			Best-fit values	0.3263	0.5828	1.157	0.6017	N/A
			95% Confidence Intervals	0.2601 to 0.4380	0.4762 to 0.7351	0.8118 to ???	0.3183 to 1.430	N/A
7 I	Functional ELISA Cluster IV LRP-1	Calculated Vmax (a.u)		***IDSco***	***IDS.IGF2co***	***IDS.ApoE2co***	***IDS.RAP12x2co***	***GFP***
			Best-fit values	-11.08	N/A	115.6	185.2	N/A
			95% Confidence Intervals	-infinity to ???	N/A	100.4 to 133.9	-45.25 to +infinity	N/A
		Calculated kd (ng/ml)		***IDSco***	***IDS.IGF2co***	***IDS.ApoE2co***	***IDS.RAP12x2co***	***GFP***
			Best-fit values	18155	N/A	319.2	23930	N/A
			95% Confidence Intervals	-infinity to +infinity	N/A	196.7 to 509.2	-9236 to +infinity	N/A
7 J	Functional ELISA Domain 11 CI-M6P/IGF2R	Calculated Vmax (a.u)		***IDSco***	***IDS.IGF2co***	***IDS.ApoE2***	***IDS.RAP12x2***	***GFP***
			Best-fit values	9.075	122	N/A	N/A	N/A
			95% Confidence Intervals	??? to 12.14	104.8 to 144.5	N/A	N/A	N/A
		Calculated kd (ng/ml)		***IDSco***	***IDS.IGF2co***	***IDS.ApoE2***	***IDS.RAP12x2***	***GFP***
			Best-fit values	1.879	225.7	N/A	N/A	N/A
			95% Confidence Intervals	??? to 9.096	145.0 to 376.4	N/A	N/A	N/A
7 K	Brain Heparan Sulfate vs IDS activity in plasma	Plateau (µg/mg HS)		***IDSco***	***IDS.IGF2co***	***IDS.ApoE2co***	***IDS.RAP12x2co***	***GFP***
			Best-fit values	1.073	0.3452	0.1975	1.149	N/A
			95% Confidence Intervals	0.9062 to 1.203	0.3052 to 0.3849	0.1095 to 0.2829	0.4975 to 1.549	N/A
		λ, Exponential decay constant (1/(nmol/mg*4hrs))		***IDSco***	***IDS.IGF2co***	***IDS.ApoE2co***	***IDS.RAP12x2co***	***GFP***
			Best-fit values	0.03096	0.0869	0.0525	0.03132	N/A
			95% Confidence Intervals	0.02293 to 0.04954	0.08168 to 0.09262	0.03942 to ???	0.01457 to 0.09530	N/A
S8 A	VCN in bone marrow vs VCN in Brain/VCN in Liver	Slope (a.u.)		**Brain**		**Liver**		
			Best-fit values	0.00264		0.04911		
			95% Confidence Intervals	0.0008020 to 0.004478		0.03599 to 0.06223		

Table S2 - Alcian Blue Scoring Rules for Meninges and Choroid Plexus

Scoring of alcian blue staining in choroid plexus and meninges	
Score	Rules
1	Weak alcian blue staining
2	Mild alcian blue staining
3	Strong alcian blue staining
4	Very strong alcian blue staining
